# Deep Sequencing Analysis of Small Noncoding RNA and mRNA Targets of the Global Post-Transcriptional Regulator, Hfq

**DOI:** 10.1371/journal.pgen.1000163

**Published:** 2008-08-22

**Authors:** Alexandra Sittka, Sacha Lucchini, Kai Papenfort, Cynthia M. Sharma, Katarzyna Rolle, Tim T. Binnewies, Jay C. D. Hinton, Jörg Vogel

**Affiliations:** 1 Max Planck Institute for Infection Biology, RNA Biology, Berlin, Germany; 2 Institute of Food Research, Norwich Research Park, Norwich, United Kingdom; 3 Center for Biological Sequence Analysis, Technical University of Denmark, Lyngby, Denmark; Stanford University, United States of America

## Abstract

Recent advances in high-throughput pyrosequencing (HTPS) technology now allow a thorough analysis of RNA bound to cellular proteins, and, therefore, of post-transcriptional regulons. We used HTPS to discover the *Salmonella* RNAs that are targeted by the common bacterial Sm-like protein, Hfq. Initial transcriptomic analysis revealed that Hfq controls the expression of almost a fifth of all *Salmonella* genes, including several horizontally acquired pathogenicity islands (SPI-1, -2, -4, -5), two sigma factor regulons, and the flagellar gene cascade. Subsequent HTPS analysis of 350,000 cDNAs, derived from RNA co-immunoprecipitation (coIP) with epitope-tagged Hfq or control coIP, identified 727 mRNAs that are Hfq-bound *in vivo*. The cDNA analysis discovered new, small noncoding RNAs (sRNAs) and more than doubled the number of sRNAs known to be expressed in *Salmonella* to 64; about half of these are associated with Hfq. Our analysis explained aspects of the pleiotropic effects of Hfq loss-of-function. Specifically, we found that the mRNAs of *hilD* (master regulator of the SPI-1 invasion genes) and *flhDC* (flagellar master regulator) were bound by Hfq. We predicted that defective SPI-1 secretion and flagellar phenotypes of the *hfq* mutant would be rescued by overexpression of HilD and FlhDC, and we proved this to be correct. The combination of epitope-tagging and HTPS of immunoprecipitated RNA detected the expression of many intergenic chromosomal regions of *Salmonella*. Our approach overcomes the limited availability of high-density microarrays that have impeded expression-based sRNA discovery in microorganisms. We present a generic strategy that is ideal for the systems-level analysis of the post-transcriptional regulons of RNA-binding proteins and for sRNA discovery in a wide range of bacteria.

## Author Summary

The past decade has seen small regulatory RNA become an important new mediator of bacterial mRNA regulation. This study describes a rapid way to identify novel sRNAs that are expressed, and should prove relevant to a variety of bacteria. We purified the epitope-tagged RNA-binding protein, Hfq, and its bound RNA by immunoprecipitation from the model pathogen, *Salmonella enterica* serovar Typhimurium. This new strategy used Next Generation pyrosequencing to identify 727 Hfq-bound mRNAs. The numbers of sRNAs expressed in *Salmonella* was doubled to 64; half are associated with Hfq. We defined the exact coordinates of sRNAs, and confirmed that they are expressed at significant levels. We also determined the Hfq regulon in *Salmonella*, and reported the role of Hfq in controlling transcription of major pathogenicity islands, horizontally acquired regions, and the flagellar cascade. Hfq is reported to be a global regulator that affects the expression of almost a fifth of all *Salmonella* genes. Our new approach will allow sRNAs and mRNAs to be characterized from different genetic backgrounds, or from bacteria grown under particular environmental conditions. It will be valuable to scientists working on genetically tractable bacteria who are interested in the function of RNA-binding proteins and the identification of sRNAs.

## Introduction

Until now, global gene expression control studies have generally focussed on the transcriptional regulation exerted by the specific action of DNA binding proteins, and on the post-translational regulation governed by specific protein–protein interactions. In comparison, little is known about how RNA binding proteins facilitate the global control of gene expression at the post-transcriptional level. However, the latest discoveries of many small noncoding RNAs (sRNAs) in both pro- and eukaryotes have shown that the interaction of RNA with proteins plays a prominent role in the regulation of cellular processes. In bacteria, the majority of the sRNAs basepair with target mRNAs to regulate their translation and/or decay [1],[2],[3], and these regulatory events commonly require the bacterial Sm-like protein, Hfq [4],[5].

Hfq is one of the most abundant RNA-binding proteins in bacteria [6],[7],[8]. First identified in *Escherichia coli* as a host factor required for phage Qβ RNA replication ∼40 years ago [9], Hfq is now known to have an important physiological role in numerous model bacteria [5]. Almost half of all sequenced Gram-negative and Gram-positive species, and at least one archaeon, encode an Hfq homologue [10],[11]. Hfq interacts with regulatory sRNAs and mRNAs, and much of its post-transcriptional function is caused by the facilitation of the generally short and imperfect antisense interactions of sRNAs and their targets [12],[13],[14],[15],[16],[17]. However, Hfq can also act alone as a translational repressor of mRNA [18],[19], and can modulate mRNA decay by stimulating polyadenylation [20],[21]. In addition, roles of Hfq in tRNA biogenesis have recently been described [22],[23].

The pleiotropy of an *hfq* deletion mutation was first apparent from the multiple stress response-related phenotypes in *E. coli* [24], and partly reflects the reduced efficiency of translation of *rpoS* mRNA, encoding the major stress sigma factor, σS [25],[26]. However, Hfq clearly impacts on bacterial physiology in a much broader fashion, including the σS-independent control of virulence factors in pathogenic bacteria (e.g., [27],[28],[29],[30],[31],[32],[33]). Specifically, deletion of *hfq* attenuates the ability of the model pathogen *Salmonella enterica* serovar Typhimurium (*S.* Typhimurium) to infect mice, to invade epithelial cells, to secrete virulence factors and to survive inside cultured macrophages [32]. Loss of Hfq function also results in a non-motile phenotype for *Salmonella* and the deregulation of >70 abundant proteins, including the accumulation of outer membrane proteins (OMPs); the latter is accompanied by a chronic activation of the σE (σ^24^)-mediated envelope stress response [32],[34]. Hfq has also been implicated in the control of *Salmonella* gene expression changes induced by the low gravity condition experienced during spaceflight [35].

Understanding how Hfq controls *Salmonella* gene expression at the post-transcriptional level requires the identification of its sRNA and mRNA ligands. In a pioneering global study in *E. coli*, Zhang *et al.* (2003) used co-immunoprecipitation (coIP) with Hfq-specific antisera and direct detection of the bound RNAs on genomic high-density oligonucleotide microarrays. Although this method proved highly effective for detecting diverse sRNAs and mRNAs in *E. coli*, the requirement for high-density microarrays and specialized antibodies has hampered similar studies in other bacteria. An alternate approach identified individual abundant Hfq-associated RNAs by cDNA cloning or direct sequencing [29],[36]; however, these methods are not appropriate for large-scale analyses. 

**Figure 1:**
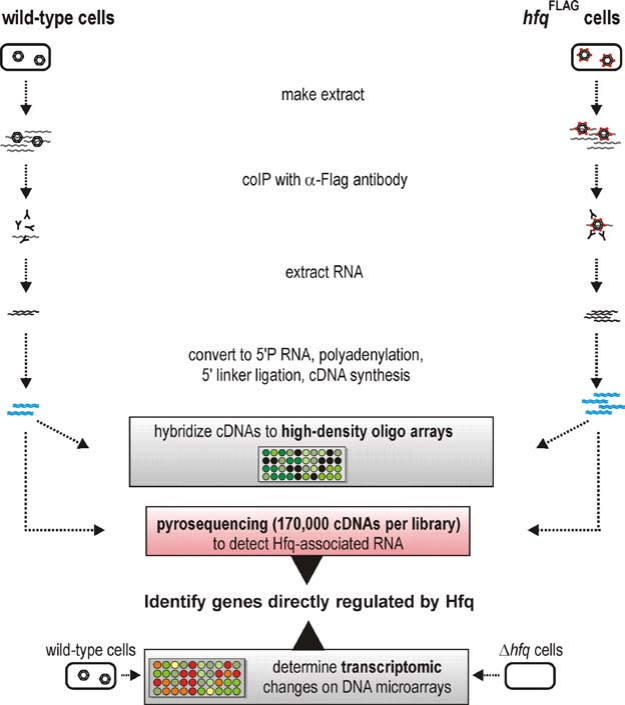
Strategy to identify Hfq targets. RNA was co-immunoprecipitated with Hfq in extracts from ESP-grown *Salmonella* cells (wild-type and chromosomal *hfq* ^FLAG^ strain) using an anti-FLAG antibody. The extracted RNA was converted to 5′ monophosphate RNA, and subsequently into cDNA, followed by direct pyrosequencing. Our approach was validated by hybridization of cDNA to high density oligo microarrays. In addition, total RNA of the wild-type strain and its *hfq* deletion mutant was used for transcriptomic analysis using *Salmonella* SALSA microarrays.

To overcome these limitations for the global identification of Hfq targets in *Salmonella*, we have now used high-throughput pyrosequencing (HTPS, a.k.a. deep sequencing) of RNA associated with an epitope-tagged Hfq protein (Figure 1). We show that this approach recovers Hfq-binding sRNAs with high specificity, and identifies their boundaries with unprecedented resolution. We report the discovery of novel *Salmonella* sRNA genes, detect the expression of many conserved enterobacterial sRNA genes, and provide a set of potential mRNA targets in this model pathogen. Comparison with the transcriptomic profile of an *hfq* mutant showed that Hfq mediates its pleiotropic effects by regulating the master transcription factors of complex regulons, and explained how Hfq is required for *Salmonella* virulence. In microbiology, deep sequencing has been used extensively for genome sequencing, either of individual microbial species [37] or of bacterial communities [38]. This study is the first report that describes the use of deep sequencing to study protein-bound mRNA from bacteria, and to discover bacterial noncoding RNAs.

## Results

### Transcriptomic Profiling Reveals a Large Hfq Regulon in *Salmonella*

**Figure 2:**
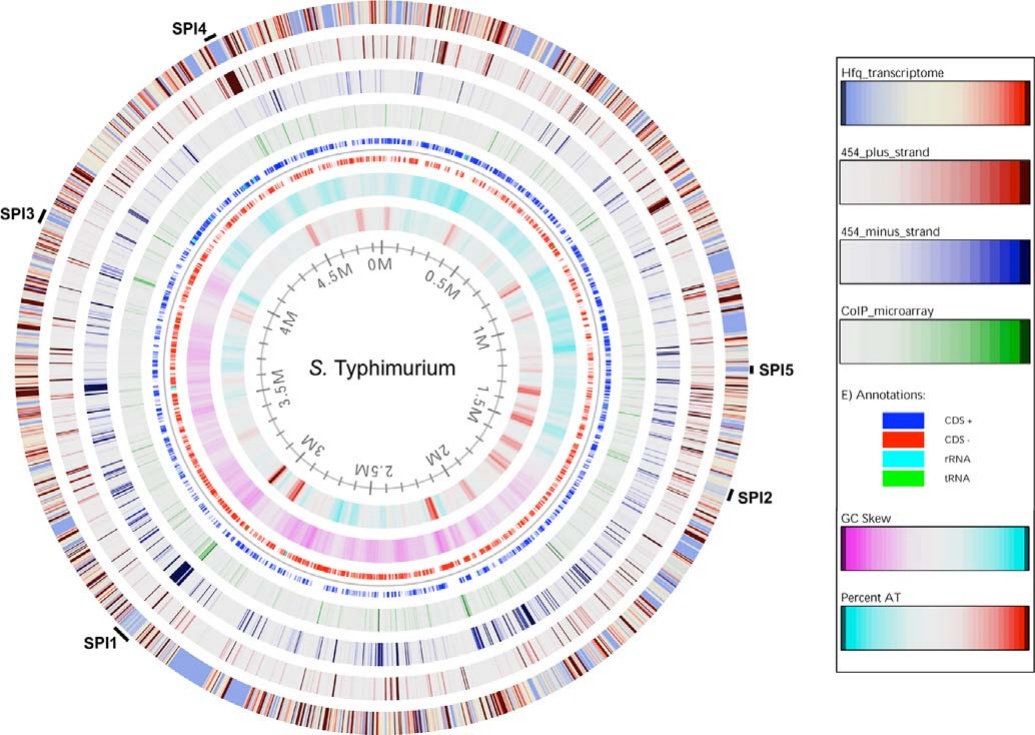
Correlation between HTPS, coIP-on-chip and transcriptomic data upon the *S.* Typhimurium chromosome. The data obtained from transcriptomic, cDNA sequencing and coIP-on-chip analyses of ESP-grown bacteria were mapped onto the *Salmonella* chromosome for direct comparison. The outer (1st) ring displays changes in gene expression in the Δ*hfq* strain compared to the parental SL1344 strain. Genes that are down-regulated in the Δ*hfq* strain are shown as blue; genes that are up-regulated are shown as red. The next three circles show regions coding for Hfq-associated RNA identified by deep sequencing (2^nd^ ring shows positive strand, and 3^rd^ ring shows negative strand) or coIP-on-chip (4^th^ ring). Ring 5 shows the location of coding sequences on the positive strand (CDS+), on the negative strand (CDS−), and the tRNA and rRNA genes. GC-skew [110] is shown in ring 6; purple and blue regions have a GC skew that is below or above the genomic average, respectively. AT-content is shown in ring 7; blue and red regions have an AT-content that is below or above the genomic average, respectively. Numbers on the inside of the innermost circle are the location relative to position zero measured in millions of base-pairs (Mbp) of the *Salmonella* LT2 genome. The location of the SPI-1 to SPI-5 is indicated. An invaluable zoomable version of this atlas is available online at http://www.cbs.dtu.dk/services/GenomeAtlas/suppl/zoomatlas/zpidStyphimurium_LT2_Atlas ; click on the region of interest to accurately visualize the data at the level of individual genes.

To detect genes that are, directly or indirectly, regulated by Hfq the transcriptomic mRNA profile of the *Salmonella* wild-type and of mutant strain JVS-0255 (Δ*hfq*) was determined. We used two different conditions for the comparison; aerobic growth in L-broth to early stationary phase (ESP; OD_600_ of 2) was chosen because the *hfq* mutation causes drastic protein pattern changes in ESP *Salmonella* [32], and overnight growth in high-salt medium under oxygen limitation (SPI-1-inducing conditions) to specifically activate the *Salmonella* virulence genes required for host cell invasion [39]. Hfq-dependent mRNAs that showed statistically significant changes (≥2-fold) were identified, and we discovered that 734 genes were differentially expressed in the Δ*hfq* strain grown to ESP (279 up-regulated genes, 455 down-regulated genes, Figure 2 and Table S1). Of the 71 proteins known to be Hfq-dependent (as determined by protein levels on 2D gels; [32]), 50% were regulated by Hfq at the transcriptional level (Table S1). Consequently, Hfq controls the expression of 17% of all *Salmonella* genes at ESP (based on the 4425 annotated ORFs; [40]). Growth under SPI-1 inducing conditions revealed 164 differentially expressed genes in Δ*hfq* (91 up-, 73 down-regulated; Table S2). 69% of these genes overlapped with the changes seen in ESP. Taken together, Hfq affects at least 785 genes, or 18% of the *Salmonella* genome.

Classification of the genes deregulated at ESP (Table 1) showed that Hfq impacted upon 26 of the 107 functional groups annotated for *Salmonella* [41]; in seven groups ≥50% of all genes were misregulated. In four of the five major *Salmonella* pathogenicity islands (i.e., SPI-1, -2, -4, -5), and in the flagellar and chemotaxis pathways, >60% of genes were down-regulated, which explains the previously observed invasion and motility phenotypes of Δ*hfq* [32]. Because Hfq affects the mRNAs of σS (RpoS) and σE (RpoE) [25],[26],[34],[42], two major alternative stress σ factors of enterobacteria, we quantified the expression of these sigma factors in *Salmonella* at the mRNA level (ESP) and at the protein level (ESP and SPI-1 inducing conditions). σE mRNA and protein levels were strongly elevated in Δ*hfq* under both conditions tested (Figure S1), confirming the previously observed chronic induction of the envelope stress response. Levels of *rpoS* mRNA were slightly increased, yet RpoS protein levels were strongly decreased. This reflects the poor translation of *rpoS* mRNA in the absence of Hfq (Figure S1 and [25],[26]). We used published lists of σE- and σS-dependent genes of *Salmonella* [43],[44] to determine how the Hfq-dependent changes we observed were related to the σE and/or σS regulons. We discovered that 55% (41/75) and 73% (54/74) of σE- and σS-dependent genes were also Hfq-dependent. Therefore, a proportion of the Hfq-dependent gene expression changes observed at ESP and under SPI-1 inducing conditions were indirect effects caused by modulation of σS and σE levels by Hfq.

**Table 1:** Pathway clustering of Hfq-dependent genes at ESP.

pathway^a^	genes in pathway^b^	% up^c^	% down^d^	% genes regulated
**Flagellar system**	**53**	**0**	**87**	**87**
**Chemotaxis**	**19**	**0**	**84**	**84**
Fimbrial proteins	24	0	20	20
**SPI1**	**39**	**0**	**90**	**90**
**SPI2**	**40**	**0**	**72.5**	**72.5**
SPI3	29	0	14	14
**SPI4**	**6**	**0**	**100**	**100**
**SPI5**	**8**	**0**	**62.5**	**62.5**
ABC transporter	188	11	7	**28**
Cyanoamino acid metabolism	10	20	10	**30**
Cystein metabolism	15	20	0	**20**
Fatty acid metabolism & biosynthesis	20	15	15	**30**
Fructose & mannose metabolism	64	2	11	**13**
Glutamate metabolism	29	7	7	**14**
Lipopolysaccharidee biosynthesis	28	3.5	3.5	**7**
Glycerophospholipid metabolism	24	17	12.5	**29.5**
Glycine, serine & threonine metabolism	35	31.5	3	**34.5**
Glycolysis/Gluconeogenesis	28	3	21	**24**
Nitrogen metabolism	33	15	6	**21**
Pentose phosphate pathway	32	12.5	19	**31.5**
Purine metabolism	73	11	4	**15**
Pyrimidine metabolism	49	10	0	**10**
Pyruvate metabolism	49	12	0	**12**
Ribosome	78	35	0	**35**
**Selenoamino acid, sulfur metabolism**	**18**	**33**	**17**	**50**
Starch & sucrose metabolism	31	3	26	**29**

Hfq-dependent genes in ESP-grown *Salmonella* are shown in Table S1.

a Pathway classification according to KEGG (http://www.genome.jp/kegg/; [21]). Pathways in which ≥50% of genes are Hfq-regulated are shadowed.

b Number of genes involved in pathway (acc. KEGG).

c,d Numbers in percent of genes that were up- or down-regulated in Δ*hfq* compared to wt, (Table S1).

The *S.* Typhimurium genome contains about 444 genes acquired by horizontal gene transfer (HGT; [45]). 122 or 17 of these HGT genes were Hfq-dependent under ESP or SPI-1 inducing conditions, respectively (16 genes being Hfq-dependent under both conditions; Tables S1, S2). In other words, Hfq regulates 28% of the HGT genes, significantly more than the 18% regulated when using the entire *Salmonella* genome for calculation. This may indicate a role of Hfq in the acquisition of DNA from foreign sources, by regulating expression of newly acquired genes at the RNA level.

### Deep Sequencing of Hfq-Associated RNAs

**Figure 3:**
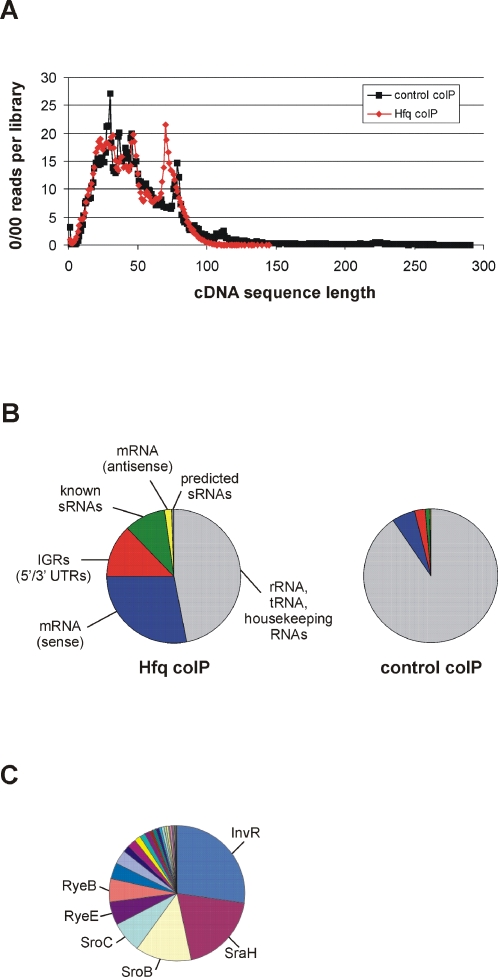
Statistical analysis of the cDNA sequencing results of Hfq-associated RNA. (A) The pyrosequencing results were analyzed by plotting the number of cDNA reads over the read length in bp. The length distribution of all resulting sequences is shown. (B) Pie diagram showing the relative proportions of the different RNA species contained in all sequences that mapped to the *Salmonella* genome. The rRNA, tRNA and housekeeping RNAs are shown in grey. Left panel: Hfq coIP, right panel: control coIP. (C) Pie diagram showing the relative proportions of all Hfq-associated sequences that unequivocally mapped to known sRNA sequences. The names of the six most frequently recovered sRNAs are given.

The variety of transcriptional regulons that showed Hfq-dependent expression patterns could either be mediated by the binding of certain regulatory sRNAs or of specific mRNAs by Hfq. To identify the direct Hfq targets we co-immunoprecipitated RNA with the chromosomally FLAG epitope-tagged Hfq protein expressed by a *Salmonella hfq* ^FLAG^ strain [46]. CoIP was performed in extracts prepared from ESP-grown bacteria. The Hfq-associated RNA was converted to cDNA, and a total of 176,907 cDNAs pooled from two independent biological experiments was then characterised by high-throughput pyrosequencing [37]. The resulting sequences, from here on referred to as “Hfq cDNAs”, ranged in length from 1 to 145 bp, and 92% were ≥18 bp (Figure 3A). Disregarding small cDNAs (<18 bp), 122,326 sequences were unequivocally mapped to the *Salmonella* genome by WU-BLAST searches (http://blast.wustl.edu/; Figure 2). About half of the mapped cDNAs (57,529) were derived from rRNA, tRNA, and housekeeping RNAs (tmRNA, M1 RNA, and SRP RNA; Figure 3B). Of the remaining 64,797 sequences, the majority corresponded to mRNA regions (53% matched the sense strand of protein-coding regions), followed by known/predicted conserved sRNAs (18%; [47]; for distribution see Figure 3C), predicted *Salmonella*-specific sRNAs (1%; [46]) and sequences that were antisense to ORF regions (3%). The remaining 25% of cDNAs mostly represented intergenic regions (IGRs) and 5′/3′ UTRs, with a few antisense transcripts to tRNAs, rRNAs, and sRNAs (0.1%; Figure 3B).

To confirm that our procedure did effectively enrich Hfq-associated RNAs, we analyzed 175,142 cDNAs from a control coIP using wild-type *Salmonella* (expressing untagged Hfq). Of these “Control cDNAs” which ranged in length from 1 to 290 bp (Figure 3A), 145,873 sequences were ≥18 bp in size and could be correlated to the *Salmonella* chromosome. Most of the inserts (91%) were abundant rRNA, tRNA, and housekeeping RNA transcripts (Fig 3B). The remaining 13,725 sequences were used to calculate the level of enrichment of Hfq-bound RNA (see below).

### Visualizing Hfq-Dependent RNAs at the Nucleotide Level

**Figure 4:**
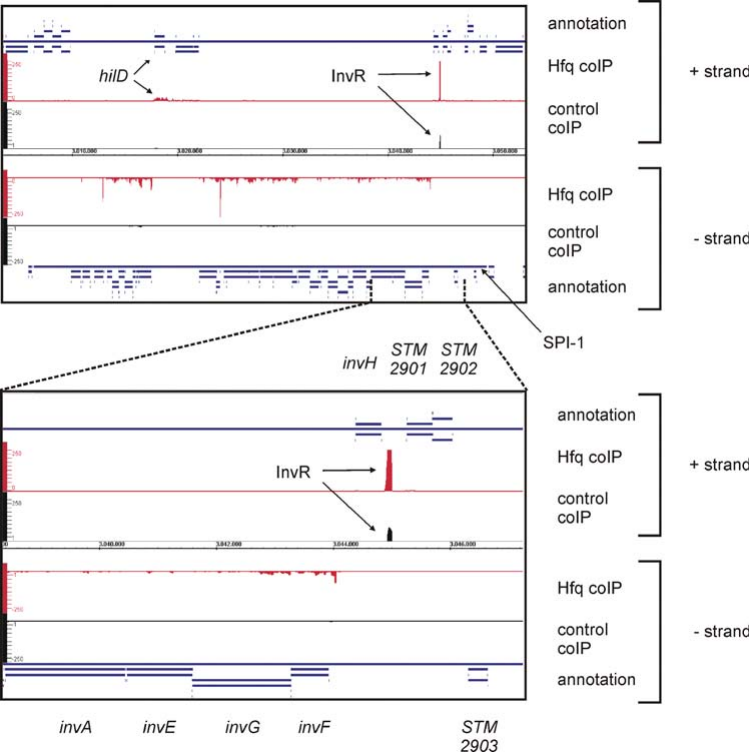
Visualization of pyrosequencing data for the *Salmonella* pathogenicity island 1 (SPI-1) with the Integrated genome Browser (Affymetrix). The upper panel shows an extraction of the screenshot of the Integrated Genome Browser, with the mapped Control and Hfq cDNAs of the SPI-1 region. Shown are the annotations for the “+” and “–” strand (blue), the cDNA sequence distribution from the Hfq coIP for the “+” and “–” strand (red), the cDNA-clone distribution for the control coIP for the “+” and “–” strand (black), and the genome coordinates in the center for the entire SPI-1. The annotation for SPI-1 and the Hfq coIP peaks for *hilD* and the sRNA InvR in the Hfq coIP are indicated. Note, that the clone numbers per nucleotide are scaled to a maximum of 250 for the Hfq and the control coIP, which truncates the high peak for InvR in the Hfq coIP library (>3000 cDNAs). The lower panel shows a close up of the *invR* locus and its adjacent genes.

Upon WU-BLAST matching, the number of cDNA hits for each nucleotide position for either strand of the *Salmonella* chromosome was calculated, and visualized using the *Integrated Genome Browser (IGB, Affymetrix)*. This browser allows the visualization of both whole genomes and individual genomic regions. Figure 4 shows the distribution of cDNA sequences over a subsection of the genome, i.e. the ∼40 kb SPI-1 virulence region, for which we observed strong enrichment of Hfq cDNAs over the Control cDNAs. As well as the 35 mRNAs of protein-coding genes, SPI-1 encodes the Hfq-dependent InvR sRNA [46]. Enrichment of InvR by coIP with FLAG-tagged Hfq was previously demonstrated by Northern blot analysis [46], and this result is confirmed by the strong cDNA peak seen at the *invR* locus located at the right-hand SPI-1 border (Figure 4).

### Hfq-Dependent sRNAs Are Highly Associated with Hfq

Inspection of the cDNA libraries revealed that a major class were derived from sRNA regions. These sRNAs, as well as their enrichment by Hfq coIP, are listed in Tables 2 and S3. The three most abundant sRNAs, according to the numbers of Hfq cDNA sequences are InvR, SraH (a.k.a. RyhA) and SroB (RybC), and are known to be strongly bound by Hfq [17],[46]; coIP of Hfq enriched these three sRNAs by 30- to 57-fold, in comparison to the control reaction. For example, InvR, which binds Hfq with a *k*_D_ of 10 nM [46], was represented by 3,236 Hfq cDNAs and 113 Control cDNAs (Table 2). In contrast, other sRNAs not expected to be Hfq-dependent were found in equal numbers in the two samples. For example, the CsrB or CsrC sRNAs which target the conserved RNA-binding protein, CsrA [48], were represented by almost equal numbers in the Hfq and Control cDNAs (CsrB, 67/69; CsrC, 63/64; Table 2). Moreover, cDNAs of the abundant yet Hfq-independent 6S RNA [49] were found in smaller numbers in the Hfq than in the control library (451 versus 836; Table 2).

**Table 2:** Compilation of expressed *Salmonella* sRNAs and their enrichment by Hfq coIP.

sRNA^a^	Alternative IDs b	Identification c	Adjacent genes d	Orientation e	5′ end f	3′ end f	cDNA reads control coIP g	cDNA reads Hfq coIP h	Enrichment i	Northern j
***sgrS***	*ryaA*	**I**	*yabN/leuD*	← → ←	128574	128812	3	61	20.3	
***isrA***	-	**II**	STM0294.ln/STM0295	→ → →	339338	339760	0	0		
***sroB***	*rybC*	**I**	*ybaK/ybaP*	← → ←	556005	556085	27	1530	56.7	
***sroC***	-	**I**	*gltJ/gltI*	← ← ←	728913	728761	26	898	34.5	
***rybB***	p25	**III**	STM0869/STM0870	→ ← ←	942632	942554	3	103	34.3	
**STnc490** k	-	**IV**	*clpA/tnpA_1*	→ ← →	1024975	1025165	75	385	5.1	∼85 nt
***isrB-1***	-	**II**	*sbcA*/STM1010	← → ←	1104179	1104266	2	4	2.0	
**STnc500**	-	**IV**	STM1127/STM1128	← ← ←	1216157	1216440	7	84	12.0	∼65 nt
**STnc150**	-	**V**	*icdA*/STM1239	→ ← →	1325914	1325649	0	1	≥1.0	∼90 nt
***isrC***	-	**II**	*envF/msgA*	← → ←	1329145	1329432	0	1	≥1.0	
**STnc520**	-	**IV**	STM1248/STM1249	→ ← ←	1332809	1334044	12	100	8.3	∼80 nt
***isrD***	-	**II**	STM1261/STM1263	→ ← →	1345788	1345738	0	0		
***ryhB-2***	*isrE*	**II**	STM1273/*yeaQ*	→ ← →	1352987	1352875	0	0		
**STnc540**	-	**IV**	*himA/btuC*	→ → →	1419369	1419570	7	23	3.3	∼85 nt
***rprA***	IS083	**I**	*ydik/ydil*	← ← ←	1444938	1444832	37	286	7.7	
***rydB***	tpe7, IS082	**I**	*ydiH*/STM1368	→ → ←	1450415	1450519	4	10	2.5	
**STnc570** l	*yneM* ORF	**IV**	*ydeI/ydeE*	→ ← ←	1593723	1594413	2	21	10.5	∼190 nt
**STnc560**		**IV**	*ydeI/ydeE*	→ → ←	1593723	1594413	10	290	29.0	∼90 nt
***isrF***	-	**II**	STM1552/STM1554	→ ← ←	1630160	1629871	1	0		
***rydC***	IS067	**I**	STM1638/*cybB*	→ → ←	1729673	1729738	5	245	49.0	
***micC***	IS063, tke8	**III**	*nifJ/ynaF*	→ ← →	1745786	1745678	0	15	≥15.0	
**STnc580**	-	**IV**	*dbpA*/STM1656	← ← ←	1749662	1750147	11	311	28.3	∼100 nt
***ryeB***	tpke79	**I**	STM1871/STM1872	→ ← ←	1968155	1968053	24	653	27.2	
***dsrA***	-	**I**	*yodD/yedP*	→ ← →	2068736	2068649	6	149	24.8	
***rseX***	-	**I**	STM1994/*ompS*	← → →	2077175	2077269	0	3	≥3.0	
***ryeC***	tp11	**I**	*yegD*/STM2126	→ → →	2213871	2214016	42	72	1.7	
***cyaR***	*ryeE*	**III**	*yegQ*/STM2137	→ → →	2231130	2231216	31	659	21.3	
***isrG***	-	**II**	STM2243/STM2244	← → →	2344732	2345013	0	0		
***micF***	-	**III**	*ompC/yojN*	← → →	2366913	2367005	0	11	≥11.0	
***isrH-2***	-	**II**	*glpC*/STM2287	→ ← →	2394582	2394303	0	0		
***isrH-1***	-	**II**	*glpC*/STM2287	→ ← →	2394753	2394303	0	0		
**STnc250** l	*ypfM* ORF	**V**	*acrD/yffB*	→ ← →	2596882	2596789	6	24	4.0	∼220 nt
***ryfA***	tp1	**I**	STM2534/*sseB*	→ → ←	2674934	2675228	3	6	2.0	
***glmY***	tke1, *sroF*	**I**	*yfhK/purG*	← ← ←	2707847	2707664	20	92	4.6	
***isrI***	-	**II**	STM2614/STM2616	→ ← ←	2761576	2761329	0	2	≥2.0	
***isrJ***	-	**II**	STM2614/STM2616	→ ← ←	2762031	2761957	1	0		
***isrK***	-	**II**	STM2616/STM2617	← ← ←	2762867	2762791	0	0		
***isrB-2***	-	**II**	STM2631/sbcA	→ ← →	2770965	2770872	0	0		
***isrL***	-	**II**	*smpB*/STM2690	→ ← →	2839399	2839055	0	0		
***isrM***	-	**II**	STM2762/STM2763	← → →	2905050	2905378	0	0		
***isrN***	-	**II**	STM2764/STM2765	← → ←	2906925	2907067	0	0		
***micA***	*sraD*	**I**	*luxS/gshA*	← → ←	2966853	2966926	1	128	128.0	
***invR***	STnc270	**III**	*invH*/STM 2901	→ → →	3044924	3045014	113	3236	28.6	
***csrB***	-	**III**	*yqcC/syd*	← ← ←	3117059	3116697	69	67		
***gcvB***	IS145	**III**	*gcvA/ygdI*	← → ←	3135317	3135522	12	402	33.5	
***omrA***	*rygB*	**III**	*aas/galR*	← ← →	3170208	3170122	0	51	≥51.0	
***omrB***	*t59, rygA, sraE*	**III**	*aas/galR*	← ← →	3170408	3170322	1	52	52.0	
**STnc290**	-	**V**	*tnpA_4*/STM3033	← ← ←	3194996	3194914	2	72	36.0	∼85 nt
***isrO***	-	**II**	STM3038/STM3039	← → →	3198380	3198580	0	0		
***ssrS***	-	**I**	*ygfE/ygfA*	→ → →	3222098	3222280	836	451		
***rygC***	t27	**I**	*ygfA/serA*	→ → ←	3222913	3223065	14	17	1.2	
***rygD***	tp8, C0730	**I**	*yqiK/rfaE*	→ ← ←	3362474	3362327	17	104	6.1	
***sraF***	tpk1, IS160	**I**	*ygjR/ygjT*	→ → →	3392069	3392261	0	25	≥25.0	
***sraH***	*ryhA*	**I**	*yhbL/arcB*	← → ←	3490383	3490500	55	2292	41.7	
***ryhB-1***	*sraI*, IS176	**I**	*yhhX/yhhY*	← ← →	3715495	3715401	0	2	≥2.0	
***istR-1***		**VI**	*ilvB/emrD*	← ← →	3998147	3998018	0	0		∼75 nt
***istR-2***		**VI**	*ilvB/emrD*	← ← →	3998147	3998018	0	0		∼140 nt
**STnc400**	-	**V**	STM3844/STM3845	→ → →	4051145	4051340	112	42		∼55 nt
***glmZ***	k19, *ryiA*, *sraJ*	**I**	*yifK/hemY*	→ → ←	4141650	4141854	20	196	9.8	
***Spf***	*spf*	**I**	*polA/yihA*	→ → ←	4209066	4209175	2	33	16.5	
***csrC***	*sraK*, *ryiB*, *tpk2*	**III**	*yihA/yihI*	← → →	4210157	4210400	63	64		
***isrP***	-	**II**	STM4097/STM4098	← → ←	4306719	4306866	0	2	≥2.0	
***oxyS***	-	**I**	*argH/oxyR*	→ ← →	4342986	4342866	0	10	≥10.0	
***sraL***	*ryjA*	**III**	*soxR*/STM4267	→ ← →	4505010	4504870	0	0		
**STnc440**	-	**V**	STM4310/*tnpA_6*	→ → →	4559193	4559277	9	456	50.7	∼85 nt
***isrQ***	-	**II**	STM4508/STM4509	← → →	4762997	4763158	0	0		

a Gene names of *Salmonella* sRNAs that have been experimentally proven here, and in previous studies. Method of identification is given in the third column. sRNA names follow *Salmonella* and/or *E. coli* nomenclature referenced in (Hershberg *et al.*, 2003; Padalon-Brauch *et al.*, 2008; Papenfort *et al.*, 2008), except STnc490, 500, 520, 540, 560, 570, 580, which have been newly predicted in this study (see Supplementary Table S3).

b Alternative sRNA IDs. References in (Hershberg *et al.*, 2003; Padalon-Brauch *et al.*, 2008; Papenfort *et al.*, 2008).

c Evidence for sRNAs in *Salmonella*.

(I) Conserved sRNA found in *Salmonella* cDNA libraries, and previously shown to be expressed in *E. coli* (relevant ref. in (Papenfort *et al.*, 2008); Table 1).

(II) sRNA previously predicted and validated on Northern blots in *Salmonella* by (Padalon-Brauch *et al.*, 2008).

(III) sRNA previously validated on Northern blots in *Salmonella* (Altier *et al.*, 2000; Figueroa-Bossi *et al.*, 2006; Fortune *et al.*, 2006; Papenfort *et al.*, 2006; Papenfort *et al.*, 2008; Pfeiffer *et al.*, 2007; Sharma *et al.*, 2007; Viegas *et al.*, 2007).

(IV) sRNA predicted through cDNA sequencing and validated by Northern blot analysis in this study.

(V) sRNA previously predicted by (Pfeiffer *et al.*, 2007) is recovered in cDNA sequences and validated by Northern blot analysis in this study.

(VI) IstR sRNAs (Vogel *et al.*, 2004) were not recovered in cDNA sequences but their expression in *Salmonella* validated by Northern blot analysis in this study (Figure S5).

d Flanking genes of the intergenic region in which the sRNA candidate is located.

e Orientation of sRNA candidate (middle) and flanking genes (→ and ← denote location of a gene on the clockwise or the counterclockwise strand of the *Salmonella* chromosome).

f Genomic location of sRNA candidate gene according to the *Salmonella typhimurium* LT2 genome. For STnc470 through STnc640 start and end of the entire intergenic region are given.

g Out of 145,873 sequences in total.

h Out of 122,326 sequences in total.

i Enrichment factor calculated by the number of blastable reads from Hfq coIP over control coIP.

j Denotes verification on Northern blot in this study for new RNA transcripts; the estimated size is given in nucleotides.

k The cDNA reads map antisense internally of the IS200 element. Based on sequence identity they map to all IS200 elements (*tnpA_1* to *tnpA_6*).

l STnc250 and STnc570 contain small ORFs annotated as *ypfM* or *yneM*, respectively, in *E. coli* (Wassarman *et al.*, 2001).

**Figure 5:**
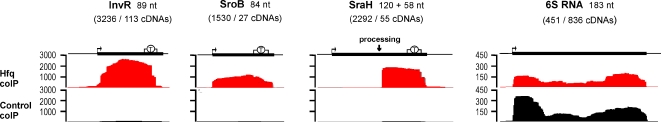
Visualization of the clone distribution of exemplar Hfq dependent and independent sRNAs in *Salmonella.* Clone distribution for sequences mapped to InvR, SroB, SraH, or 6S sRNAs (red: Hfq coIP, black: control coIP). The vertical axis indicates the number of cDNA sequences that were obtained. A bent arrow indicates each sRNA promoter, a circled “T” its transcriptional terminator.

Figure 5 illustrates the distribution of cDNAs of the three predominant Hfq-bound RNAs and of the Hfq-independent 6S RNA. cDNAs of both the InvR (89 nt; [46]) and SroB (84 nt; [50]) sRNAs mapped along the entire RNA coding sequence from the transcriptional start site to the Rho-independent terminator. SraH, which is transcribed as an unstable 120 nt precursor and processed into an abundant ∼58 nt RNA species (3′ part of SraH; [17],[51]), was almost exclusively recovered as the processed sRNA. Notably, the borders of the cDNA clusters were in perfect agreement with previous 5′ and/or 3′ end mapping data of the four sRNAs [46],[50],[51],[52]. In other words, our cDNA sequencing approach not only detects association with Hfq, but also identifies the termini of expressed sRNAs at nucleotide-level resolution.

### Identification of Expressed *Salmonella* sRNAs

To evaluate the sRNA expression profile of *Salmonella* more extensively, we analyzed three classes of sRNA candidate loci for coverage by the Hfq and Control cDNAs. First, cDNAs of *E. coli* sRNA candidate loci with predicted conservation in *Salmonella* were inspected [17],[47],[49],[50],[51],[53],[54]. Second, we counted cDNAs of *Salmonella*-specific sRNAs predicted in two recent global screens [46],[55]. Third, we manually inspected cDNAs from a third of the *Salmonella* chromosome (first 1.6 Mb) and all major five pathogenicity islands for expression patterns of IGRs indicative of new sRNA genes, and for possible enrichment by Hfq coIP. Using criteria similar to [49], our evaluation of these loci considered orphan promoter/terminator signals, and possible conservation in bacteria other than *E. coli*. Of the latter two classes of candidates (summarized in Table S3), those with an Hfq enrichment factor ≥10 and/or candidates showing strong promoter/terminator predictions were selected for Northern blot analysis. To assess sRNA expression under relevant environmental conditions, we probed RNA from five stages of growth in standard L-broth from exponential to stationary phases, and from two conditions known to strongly induce the expression of the major SPI-1 [39],[56] or SPI-2 [57] virulence regions. The results of this analysis are summarized in Table 2 (the whole set of candidates tested is shown in Table S3); including the 26 previously detected *Salmonella* sRNAs [34],[46],[55],[58],[59],[60],[61],[62],[63], a total of 64 *Salmonella* sRNAs can now be considered to be experimentally validated.

**Figure 6:**
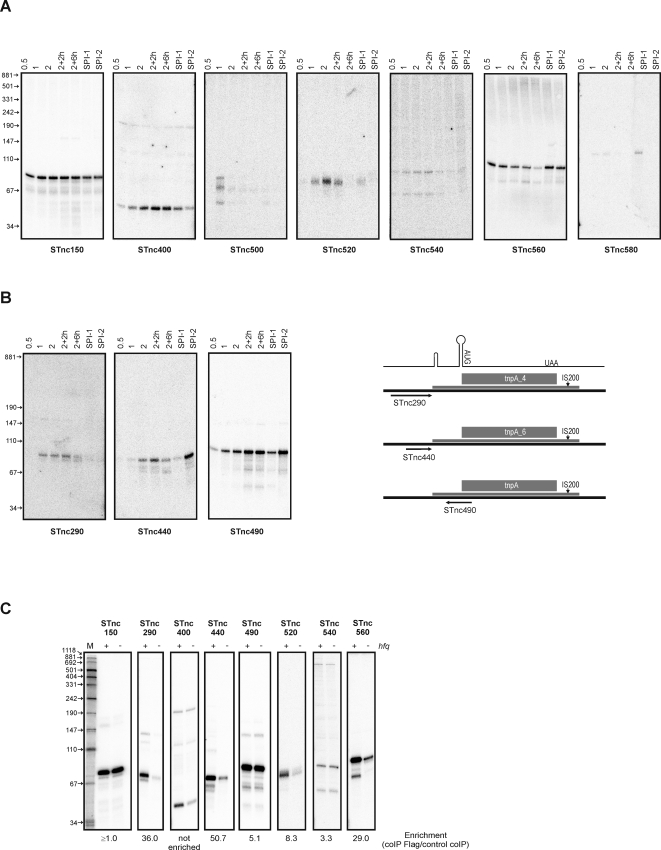
Expression of 10 new *Salmonella* sRNAs over growth. Total RNA was isolated from *Salmonella* at seven different growth stages and/or conditions and subjected to Northern blot analysis. (A) Blots showing the detection of stable transcripts for seven new sRNAs. The lanes refer to the following samples (from left to right): aerobic growth of the wild-type strain in L-broth to an OD600 of 0.5, 1 or 2; growth continued after the culture reached OD600 of 2 for 2 or 6 hours, respectively; SPI-1 inducing condition; SPI-2 inducing condition. (B) Northern blots of three sRNAs encoded in close proximity (STnc290, STnc440) or antisense (STnc490) to IS200 elements. A schematic presentation of the position of the sRNAs according to the IS200 element is shown to the right. The upper drawing indicates the two stem-loop structures, start codon, and stop codon of the transposase-encoding mRNA of the IS200 elements. The three detected sRNAs are indicated by black arrows. Growth conditions as Panel A. (C) RNA abundance of selected new sRNAs in wild-type (+) versus *hfq* mutant (−) *Salmonella* cells at ESP (OD600 of 2). The enrichment factor of each of these sRNAs in the coIP experiment is given below the blots for comparison.

We used Northern blots to detect 10 of the 31 newly identified *Salmonella* sRNAs under the environmental conditions that were tested (Figure 6, Tables 2 and S3). These sRNAs yielded stable transcripts, predominantly in the 50 to 100 nt range (Figure 6A and B). Faint bands of larger transcripts were observed for STnc150 (150 nt), and STnc400 (190 nt), resembling certain *E. coli* sRNAs such as SraH whose precursor is rapidly degraded whilst the processed form accumulates [51]. The STnc150, 400, and 560 sRNAs are almost constitutively expressed, whereas STnc500, 520 and 540 are only expressed in certain environmental conditions. Intriguingly, STnc580 can only be detected under SPI-1 inducing conditions that mimic the environment *Salmonella* encounters in the host intestine. Generally, only candidates represented by ≥20 cDNAs in a cDNA pool yielded a signal on Northern blots (Tables 2 and S3). While this suggests some correlation between intracellular abundance and cDNA frequency, we note the case of STnc150, for which a single cDNA was recovered yet a strong signal was obtained on Northern blots. In contrast, several candidates with >20 cDNAs failed the Northern blot validation (Table S3). We speculated that the corresponding cDNAs were derived from 5′ or 3′ UTRs of larger mRNA transcripts, and tested this on Northern blots of agarose gels. We tested 14 of such candidates which had the appropriate orientation to flanking mRNA genes to be UTR-derived; six of these showed signals ranging in size from 500 to 2000 nucleotides (STnc180, Stnc190, STnc330, STnc470, STnc610, and STnc640; Figure S2 and Table S3), and are likely to be processed mRNA species.

**Figure 7:**
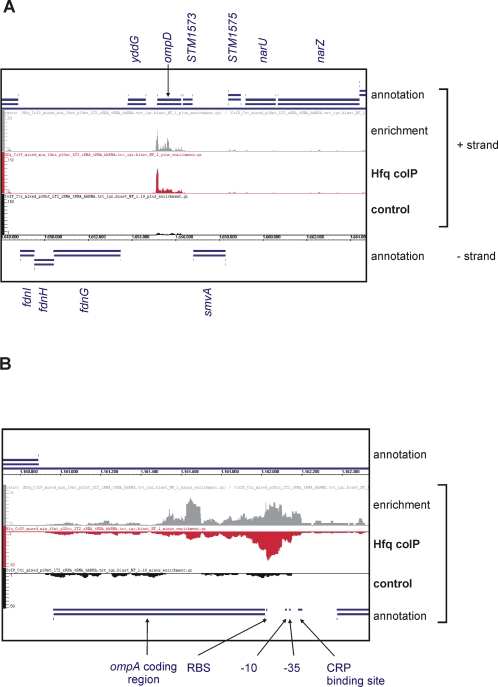
Comparison of Hfq and Control coIP cDNA distributions at the *ompD* and *ompA* loci. Extract of the screenshot of the Integrated Genome Browser, showing the mapped Hfq and Control cDNAs, and the enrichment curve (ratio of reads of Hfq coIP over control coIP) for (A) the *ompD* and (B) *ompA* transcripts. Shown are (from top to bottom) the annotations for the “+” strand (blue), the enrichment curve (grey), the cDNA distributions on the “+” strand for the Hfq coIP (red) and the control coIP (black), the genome coordinates, and the annotations for the “–” strand (blue). In panel A, the annotation of the *ompD* coding region and the flanking genes, *yddG* and STM1573, are indicated. For *ompA*, the CDS, -10 and -35 boxes, as well as the ribosome binding site (RBS) and a CRP binding site are indicated by black arrows.

**Figure 8:**
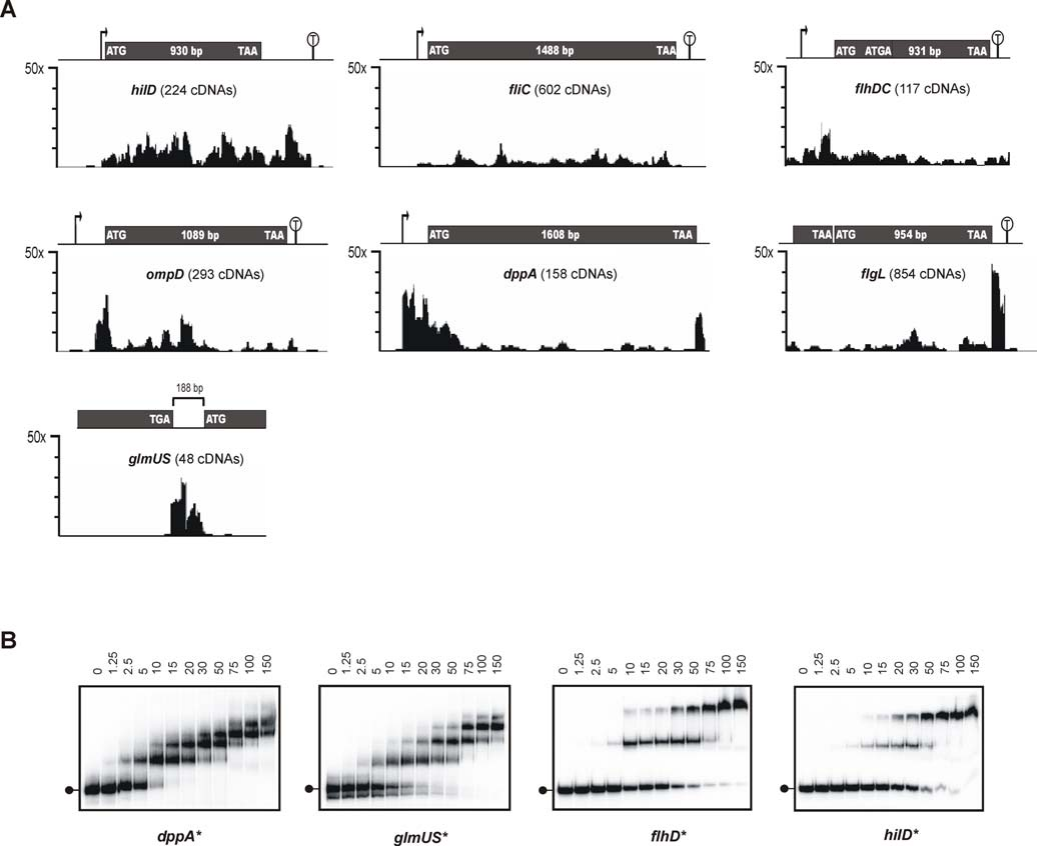
Distribution patterns of cDNAs of Hfq-associated mRNA species and confirmation of binding to Hfq. (A) Different mRNAs are shown with marked open reading frame, promoter and terminator (where known). Start and stop codons are indicated. The clone distribution is represented by a stairstep diagram of fold enrichment in Hfq coIP *vs* control coIP per nucleotide below each mRNA. The vertical axis indicates the enrichment factor in the Hfq coIP calculated over the control coIP. ORF length is given for each gene, for the overlapping ORFs of *flhDC*, or for the intergenic region in the case of *glmUS* mRNA. Numbers in parentheses below each gene name denote number of cDNA sequences obtained from Hfq coIP. Promoters and terminators are indicated as above. (B) The binding of Hfq to four mRNA fragments was confirmed by gel mobility shift assay. ^32^P-labeled RNA fragments of *dppA*, *glmUS*, *flhD*, or *hilD*, respectively, were incubated with increasing amounts of Hfq protein (concentrations of the hexamer are given in nM above the lanes). The lollipops on the left of the gel panels show the position of the unshifted mRNA fragment. Following 10 minutes incubation at 37°C, samples were resolved on native 6% polyacrylamide gels, autoradiographs of which are shown.

**Figure 9:**
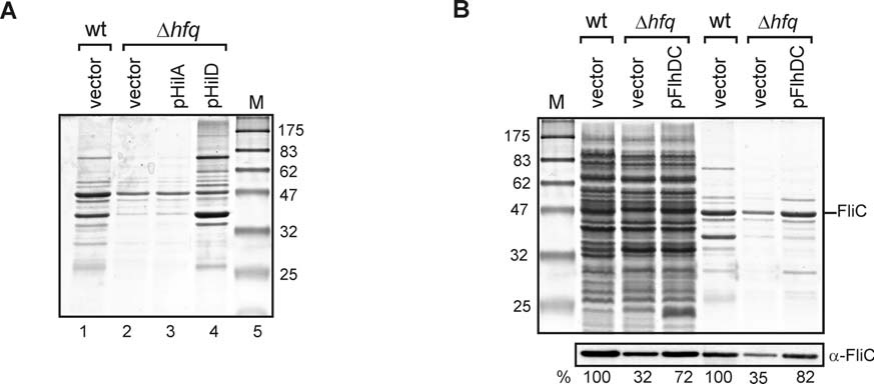
Rescue of complex Δ*hfq* phenotypes by overexpression of identified Hfq target mRNAs. SDS-PAGE analysis (12% gels stained with Coomassie) of (A) secreted proteins upon overexpression of the SPI-1 transcription factors, HilA and HilD from pCH-112 and pAS-0045 (lanes 3 and 4) in *Salmonella* Δ*hfq*. Lanes 1 and 2 show the secreted protein profile of *Salmonella* wild-type and Δ*hfq* bacteria carrying a control vector, pKP8-35. (B) Whole cell and secreted proteins upon overexpression of the flagellar transcription factor, FlhD_2_C_2_. The left hand three lanes show total protein samples, and the right hand three lanes show secreted proteins. Genetic background and plasmids are indicated above the lanes; FlhDC was expressed from plasmid pAS-0081. FliC was also analyzed on a Western blot using a specific antibody (lower panel). FliC protein levels are shown (in %), in comparison to wild-type *Salmonella*, which was set to 100% for either the total protein or secreted protein lanes.

Three sRNAs expressing stable transcripts of ∼85 to 90 nts originate from close to, or within, IS200 transposable elements (Figure 6B). STnc290 and STnc440 are expressed just upstream of *tnpA*_4 and *tnpA*_6, respectively, whereas STnc490 is antisense to the translational start site of the IS200 transposase ORF. IS200 elements generally posses two stem-loop structures, one of which is a Rho-independent transcription terminator that prevents read-through from genes located upstream of the integration site [64]. Given their location, the STnc290 sRNA could originate from processing of the STM3033 transcripts reading into the *tnpA*_4 terminator structure; by analogy, STnc440 would be derived from STM4310 transcripts. If so, this would constitute interesting cases in which transposon insertion has created stable sRNAs. The other IS200 stem-loop functions as a translational repressor by sequestering the start codon of the transposon ORF [64]; STnc490 overlaps with this structure on the opposite strand, and by acting as an antisense RNA may function as an additional repressor of IS200.

We determined whether 8 of the new *Salmonella* sRNAs showed an Hfq-dependent pattern of transcript abundance that correlated with Hfq binding (Figure 6C). The STnc290, 440, 490, 520, 540 and 560 sRNAs were all enriched by Hfq coIP (Table 2), by factors up to 51-fold (STnc440). The expression of the four sRNAs with the highest enrichment factors (STnc290, 440, 520, 560) was strongly reduced in Δ*hfq*, and so classified as Hfq-dependent; in contrast, the accumulation of STnc150, STnc490 and STnc540 (≥1.0-, 5.1-, and 3.3-fold enrichment, respectively) was unaffected in the absence of Hfq. STnc500, which is only detected in samples originating from cultures at OD_600_ of 1, and STnc580, which seems to be specifically expressed under the SPI-1 inducing condition, were not detected on these blots.

In addition to the sRNAs listed above, the cDNAs included two loci predicted to encode small peptides, i.e. shorter than the 34 amino acid cut-off used to define ORFs in the current *Salmonella* genome annotation [40]. These are referred to as STnc250 and STnc570 in Table 2, and correspond to the predicted small *ypfM* and *yneM* mRNA-encoding genes of *E. coli* [49]. Probing of the *Salmonella* loci yielded signals of stable short mRNAs which are expressed in a growth phase-dependent manner (Figure S3).

### Hfq-Associated mRNAs

To determine which of the 34,136 cDNAs were derived from Hfq-bound mRNAs and represented genuine mRNA targets, a stringent cutoff was used. An mRNA coding region (CDS) was required to be represented by ≥10 cDNAs to be considered significant, which identified 727 Hfq-bound mRNAs (cistrons) for further analysis. Table 3 lists the top 42 mRNAs with at least 100 cDNAs in the Hfq coIP library (Table S4 lists all 727 mRNAs). In the genome browser, many of these enriched mRNAs were readily visible as a distinct cDNA cluster, e.g., the *ompD* mRNA (encoding the major *Salmonella* outer membrane protein) shown in Figure 7A. A survey of the transcriptomic data revealed that 33% of the Hfq-bound mRNAs showed an Hfq-dependent pattern of gene expression (Table S1). The reciprocal analysis showed that 32% of the Hfq-dependent mRNAs were bound to Hfq (Table S1). We attribute the observed partial overlap of the Hfq coIP and the transcriptomic data (33%) to three major factors. First, Hfq regulates transcription factors, de-regulation of which alters the expression of downstream genes. In other words, not every gene deregulated in the Δ*hfq* strain is necessarily a “direct” Hfq target, i.e. its mRNA bound by Hfq. Second, there may be a considerable number of Hfq-associated mRNAs below our very stringent cut-off for Hfq-association; increasing sequencing depth will overcome this problem. Third, the precise borders of most 5′/3′ UTRs are unknown in *Salmonella* (and any other bacterial genome sequence); consequently, calculations of Hfq enrichment were limited to the CDS of an mRNA. As outlined further below (Figure 7B), this can skew the overall enrichment factor.

**Table 3:** mRNAs represented by ≥100 cDNAs in the pyrosequencing data.

STM number	Gene name^a^	Number of inserts in control coIP^b^	Number of inserts in Hfq coIP^c^	Enrichment^d^	Product^e^
STM4261		254	1042	4.1	putative inner membrane protein
STM2665	*yfiA*	72	648	9.0	ribosome stabilization factor
STM1377	*lpp*	168	608	3.6	murein lipoprotein
STM4087	*glpF*	40	570	14.3	glycerol diffusion
STM1959	*fliC*	248	547	2.2	flagellar biosynthesis protein
STM2874	*prgH*	73	415	5.7	needle complex inner membrane protein
STM2267	*ompC*	63	385	6.1	outer membrane protein C precursor
STM2882	*sipA*	36	354	9.8	secreted effector protein
STM2885	*sipB*	126	335	2.7	translocation machinery component
STM4326	*aspA*	79	328	4.2	aspartate ammonia-lyase
STM2925	*nlpD*	30	300	10.0	lipoprotein
STM4086	*glpK*	115	278	2.4	glycerol kinase
STM2883	*sipD*	34	269	7.9	translocation machinery component
STM0739	*sucD*	14	261	18.6	succinyl-CoA synthetase alpha subunit
STM1572	*ompD*	76	246	3.2	putative outer membrane porin precursor
STM2898	*invG*	16	226	14.1	outer membrane secretin precursor
STM2879	*sicP*	6	224	37.3	secretion chaparone
STM2283	*glpT*	30	221	7.4	sn-glycerol-3-phosphate transport protein
STM1091	*sopB*	23	216	9.4	secreted effector protein
STM1732	*ompW*	28	206	7.4	outer membrane protein W precursor
STM0451	*hupB*	14	198	14.1	DNA-binding protein HU-beta
STM2871	*prgK*	46	198	4.3	needle complex inner membrane lipoprotein
STM2884	*sipC*	96	192	2.0	translocation machinery component
STM4406.S	*ytfK*	6	191	31.8	putative cytoplasmic protein
STM2867	*hilC*	3	187	62.3	invasion regulatory protein
STM2869	*orgB*	8	182	22.8	needle complex export protein
STM2878	*sptP*	20	177	8.9	protein tyrosine phosphatase/GTPase activating protein
STM2894	*invC*	14	175	12.5	type III secretion system ATPase
STM2875	*hilD*	23	174	7.6	invasion protein regulatory protein
STM2284	*glpA*	57	149	2.6	sn-glycerol-3-phosphate dehydrogenase large subunit
STM3526	*glpD*	39	147	3.8	sn-glycerol-3-phosphate dehydrogenase
STM2886	*sicA*	23	146	6.3	secretion chaperone
STM3138		19	143	7.5	putative methyl-accepting chemotaxis protein
STM2896	*invA*	19	142	7.5	needle complex export protein
STM0833	*ompX*	6	137	22.8	outer membrane protein X
STM2899	*invF*	18	129	7.2	invasion regulatory protein
STM2924	*rpoS*	19	129	6.8	RNA polymerase sigma factor
STM0629	*cspE*	9	125	13.9	cold shock protein E
STM2285	*glpB*	33	119	3.6	anaerobic glycerol-3-phosphate dehydrogenase subunit B
STM0736	*sucA*	42	110	2.6	2-oxoglutarate dehydrogenase
STM2445	*ucpA*	5	105	21.0	short chain dehydrogenase
STM1070	*ompA*	77	102	1.3	putative hydrogenase membrane component precurosr

a Gene names according to ColiBase (Chaudhuri et al., 2004)

b Based on 145,873 sequences

c Based on 122,326 sequences

d Enrichment factor calculated by the number of blastable reads from Hfq coIP over control coIP.

e Product according to KEGG (http://www.genome.jp/kegg/; (Goto et al., 1997)).

To validate our cDNA sequencing approach for the detection of Hfq-bound mRNAs by the conventional approach, we hybridized the RNA obtained from Hfq and control coIP to a *S.* Typhimurium oligonucleotide microarray. Analysis of this coIP-on-Chip experiment with SAM-software (Statistical Analysis of Microarrays; FDR<0.01) identified 365 enriched mRNAs. Nearly half (45%) of these mRNAs corresponded to regions identified by the deep sequencing approach (Table S5; *P*<10e-10). The overlap increased to 67% when genes that showed enrichment values above 5 were taken into consideration. Although coIP-on-Chip displays a lower sensitivity than deep sequencing these two independent methods do generate comparable results for the identification of mRNA-protein interactions.

Genome annotations of protein-coding genes are generally limited to the mRNA coding regions (CDS). Whilst Tables 3 and S4 list absolute hit numbers in annotated CDS, the detailed analysis of cDNA distribution over a given mRNA gene often revealed a more complex picture. For example, the number of *ompA* cDNAs does not drastically differ in the two libraries (Hfq coIP, 102; control coIP, 77), which would question whether *ompA* is an Hfq-bound mRNA. However, up to 12-fold enrichment is seen in some sections of the *ompA* mRNA, e.g., around the AUG and in the central CDS (Figure 7B). Note that the availability of cDNA hit numbers for every single nucleotide of the *Salmonella* chromosome offers the possibility to also analyze 5′ and 3′ UTRs of mRNAs, which are not included in Tables 3 and S4, but could also be targeted by Hfq.

Figure 8A further illustrates the complex enrichment patterns of diverse mRNAs, some of which may be explained by previous data obtained for these transcripts, as discussed below. *i)* cDNAs of Hfq-bound mRNAs of *hilD* (encoding a key transcription factor of the *Salmonella* invasion gene island, SPI-1), *fliC* (which encodes a major flagellin), or *flhDC* (encoding the major transcription factor of the *Salmonella* flagellar genes) were distributed over the entire length of the relevant gene, including the ∼300 nt 3′ UTR in the case of *hilD*. Either Hfq does target such a large number of sites on these three mRNAs, or alternatively, given that Hfq is a ribosome-bound protein, these cDNAs may derive from polysome-bound mRNAs. *ii)* cDNAs of *ompD* were also distributed over the entire *ompD* locus, and abruptly ended 50 nt downstream of the *ompD* stop codon, at the predicted Rho-independent terminator; a major cDNA cluster was observed around the *ompD* AUG start codon, i.e. the −70 to +19 region (for separate display of control coIP, Hfq coIP, and enrichment curves see Figure 7A). Intriguingly, this particular region binds Hfq with high affinity *in vitro* (*k*_D_≤1 nM; [32]). Binding of Hfq to the *ompD* AUG region may control translation initiation analogous to the Hfq-mediated repression of the *E. coli ompA* mRNA [18]. Similarly, cDNAs representing *dppA* clustered at the 5′ end of this mRNA, from the transcriptional +1 site into the N-terminal (signal peptide) coding region. The Hfq-dependent sRNA, GcvB, is known to target the *dppA* 5′ UTR [58], and our data suggest that Hfq-binding to this *dppA* region could enhance GcvB action. *iii)* cDNA clones of the ∼10kb *flgBCDEFGHIJKL* mRNA (flagellar components) were almost exclusively derived from the terminal, 80 nt region downstream of the *flgL* stop codon which includes the terminator. It is possible that Hfq controls flagellar operon mRNA expression through modulation of mRNA decay initiating at the 3′ end. *iv)* Almost all of the 48 cDNAs of the dicistronic *glmUS* mRNA mapped in two clusters to the *glmUS* IGR (188 nt). cDNAs of the upstream cluster start with the adenosine of the *glmU* UGA stop codon and span the first 73 nt of the IGR. In *E. coli, glmUS* mRNA undergoes RNaseE-dependent cleavage within the *glmU* UGA to generate a monocistronic *glmS* mRNA [65],[66]; the *glmS* mRNA is activated by the GlmZ sRNA, which binds Hfq [49] and the *glmUS* IGR [19]. As mentioned for GcvB/*dppA*, Hfq is likely to aid the binding of GlmZ to the *glmUS* mRNA in the region of the two clusters of cDNAs.

It is worth noting that the extended steps of lysate preparation and antibody incubation involved in the Hfq coIP protocol do cause some mRNA degradation [17]. Our Northern blots did not detect full-length mRNA in the RNA samples from the *Salmonella* Hfq coIP (data not shown). We believe that the sequenced cDNAs were synthesized from a mixture of shorter cDNA fragments, rather than from intact transcripts of several kb in length. The short cDNAs that were prepared from Hfq coIP have the advantage of favoring the primary Hfq binding region.

To confirm that Hfq bound to enriched mRNA regions, corresponding fragments of the *dppA*, *glmUS, flhD* and *hilD* mRNAs were *in vitro-*synthesized, and analyzed in gel mobility shift assays (Figure 8B). These RNA fragments were fully shifted by addition of ≤50 nM Hfq hexamer, which suggested significantly stronger binding than to the previously tested, non-specific *metK* mRNA (*k*_D_≥250 nM; [32]) which is not regulated by Hfq and was not recovered by Hfq coIP (Tables S1 and S4). Thus, the cDNA sequences appear to represent high-affinity, primary binding sites of Hfq on mRNAs.

### Mechanisms of Pleiotropic Hfq Effects in Virulence and Flagellar Pathways

Our analyses revealed an intriguing relationship between the transcriptomic and deep sequencing data; the genes belonging to some regulons were consistently down-regulated in the Δ*hfq* mutant, yet Hfq only associated with a few of the relevant mRNAs. For example, the transcriptomic data showed that the entire SPI-1 pathogenicity island was down-regulated in the Δ*hfq* mutant, but the Hfq coIP only showed a strong enrichment for a small subset of SPI-1 genes (*hilC, hilD*, *invFGAC, sicA, sip operon, prgHK,* and *orgB*; Tables S1, S4 and Figures 4, S4). Of these, *hilD* encodes the primary transcriptional activator of the SPI-1 region [67]. We hypothesized that loss of Hfq-association with *hilD* mRNA in Δ*hfq* causes loss of HilD protein synthesis, and thereby one of the strongest *hfq* phenotypes, i.e. loss of SPI-1 activation and virulence factor (effector protein) secretion. If so, ectopic HilD overexpression should restore SPI-1 effector secretion to Δ*hfq*. As predicted, overproduction of HilD from a P_BAD_ expression plasmid restored SPI-1 effector secretion to almost wild-type levels in the absence of Hfq (Figure 9A; compare lanes 1 and 4), and also rescued expression of the PrgI needle protein indicative of a functional SPI-1 secretion apparatus (data not shown). In contrast, ectopic production of HilA, the SPI-1 transcription factor that acts downstream of HilD, failed to influence the secretion defect of Δ*hfq*. Preliminary data from gentamicin protection assays that assess epithelial cell invasion of *Salmonella*, suggests that overexpression of HilD increased the invasion rate of the Δ*hfq* strain by a factor of ten (data not shown). Thus, by identifying the *hilD* mRNA as a direct Hfq target, we have revealed the mechanism of part of the pleiotropic virulence defect of the Δ*hfq* strain.

In an analogous situation, 87% of the flagellar genes were down-regulated in the Δ*hfq* mutant, yet Hfq primarily bound to the *fhlDC* (class I genes), *flgMN*, *flgKL, fliAZ*, *fliD*, *fliI* and *fliP* mRNAs (class II genes) and *fliC* mRNA (class III gene; Tables S1, S4 and Figure S4). *fhlDC* encodes the key transcription factor of the flagellar gene cascade, and we predicted that loss of this mRNA would account for much of the flagellar defect of Δ*hfq*, which is associated with strongly reduced levels of the major flagellin, FliC (Figure 9B). Ectopic expression of *flhDC* restored the levels of FliC to almost wild-type levels in the Δ*hfq* strain carrying the pBAD-*flhDC* plasmid (Figure 9B). We note, however, that the previously reported non-motile phenotype of Δ*hfq* on swim agar plates [32] was not rescued by *flhDC* overexpression (data not shown), presumably because the FlhD_2_C_2_-independent chemotaxis genes required for full motility are also down-regulated in the absence of Hfq (Table 1).

## Discussion

To understand how bacterial RNA binding proteins such as Hfq mediate the control of global gene expression at the post-transcriptional level, direct targets need to be identified. The first approach that was used to do this in a global fashion involved detection of RNA co-immunoprecipitated with Hfq-specific antibodies on high-density oligonucleotide microarrays, and identified new *E. coli* sRNAs and interesting properties of Hfq [17]. Similarly, microarray-based detection following co-immunoprecipitation of eukaryotic mRNA–protein complexes (mRNPs) identified endogenous organization patterns of mRNAs and cellular proteins [68]. Epitope-tagging of the yeast La homolog was successfully used for global coIP analysis [69]. However, the requirement for custom high-density microarrays and/or species-specific antibodies has impeded similar studies in other organisms. It is now apparent that the ideal sRNA discovery approach would not only detect sRNAs, but would also define their exact sequence. Given the typical genome size of model bacteria (∼5 Mb), a high-density oligonucleotide microarray with ∼10 million oligonucleotide probes would be required to achieve single basepair resolution. Such arrays do not exist for any organism, and even today's high-density arrays (with 0.5 million features) come with extraordinarily high set-up and printing costs, and are available for very few bacteria. Our strategy remedies these technical and financial limitations.

The identification of Hfq-associated RNAs in *Salmonella* is based upon a powerful chromosomal epitope-tagging approach [70], followed by coIP with a commercially-available antibody, and sequencing of hundreds of thousands cDNAs. The earlier shotgun-cloning studies in bacteria [50],[54],[71] and many other organisms (reviewed in [72],[73]) were limited by costly Sanger-type sequencing of individual cDNA inserts from plasmid vectors. The deep sequencing approach described here avoids a cloning step, and is able to detect small RNAs with unparalleled sensitivity by defining the 5′ and 3′ ends of transcripts at basepair resolution.

Deep sequencing of cDNAs has identified the small RNA component of eukaryotic transcriptomes (e.g., [74],[75]), and new classes of noncoding RNAs associated with eukaryotic RNA-binding proteins [76],[77]. These studies primarily focussed on the class of 20–30 nucleotide long microRNAs and siRNAs, and typically included size-fractionation steps. Bacterial riboregulators are considerably larger (80-250 nucleotides), and we show that even without prior size fractionation, deep sequencing can capture and define the termini of these large sRNAs.

Our analysis extends the tally of confidently identified sRNAs to 64 in the model pathogen, *S.* Typhimurium (Table 2). Thirty eight of these are conserved sRNAs that were initially identified in *E. coli*, but only a few of their homologues have previously been shown to be expressed in other enteric bacteria [58],[59],[60],[61],[62],[63],[78],[79]. A recent study of the widely conserved DsrA and RprA sRNAs [80] failed to validate their expression and/or function in *Salmonella* [81]. Our observation of 149 (DsrA) and 286 (RprA) cDNAs in the Hfq coIP libraries (versus 6/37 in the control library), unequivocally confirmed that these important stress response regulators are both expressed and Hfq-associated. The finding, from this and other studies, that highly-conserved sRNAs are commonly expressed at the transcriptional level should prove useful to researchers working in other bacterial systems.

A significant number of the Hfq-associated cDNAs correspond to sRNA loci that are absent from *E. coli* ([46],[55] and Table 2). Of these, *invR* exemplifies a sRNA gene that was likely horizontally acquired with the SPI-1 virulence region, early in *Salmonella* evolution [46]. Intriguingly, InvR is the most frequently recovered sRNA (>3,000 cDNAs in the Hfq coIP library), which shows that our approach is not only effective for detecting conserved, but also species-specific sRNAs of recently acquired pathogenicity regions. Horizontal transfer of virulence islands is a driving force in the evolution of bacterial pathogens [82], and knowledge of the functional elements of these islands is key to understanding pathogenesis. Whereas ORF identification in such islands has become routine, island-specific sRNAs are more difficult to recognize by bioinformatic-based approaches.

Besides confirming InvR, the present study found evidence for the expression of five of the 47 *Salmonella* sRNA candidate loci listed by Pfeiffer *et al.* [46], who predicted orphan promoter/terminator pairs in IGRs (Table S3 and Figure 2). One of these, i.e. STnc250, has turned out as a small mRNA gene (see above). While this study was in progress, others reported the discovery of 18 *Salmonella* expressed sRNA loci [55]. We recovered cDNAs of 8 of these sRNAs (*isrB*-1, *C*, *E*, *I-L*, and *P*; Table 2). The fact that 10 of these sRNAs were not recovered probably reflects their low-level expression under the growth condition used here [55]. This observation suggests an improvement that could be made to our method. RNomics- or microarray-based sRNA discovery methods require sRNAs to be expressed under the chosen assay condition, unlike bioinformatics-aided approaches that score for orphan transcription signals and primary sequence conservation [49],[51],[83],[84] or for conservation of RNA structure [53]. Thus, future studies combining several different growth conditions with increasing sequencing depth are likely to identify even more novel sRNAs.

Regarding the sensitivity of our approach, it is remarkable that RyeB sRNA was found in 653 Hfq cDNAs and 24 Control cDNAs (Table 2); RyeB is late stationary phase-specific [49],[50], and barely detectable by probing of *Salmonella* RNA from the coIP assay condition by Northern blot (unpublished results). Moreover, the 24 cDNAs recovered from the control library, i.e. without Hfq coIP, suggest the exciting possibility that deep sequencing of total RNA, without prior enrichment or size-fractionation, will prove to be a successful approach for sRNA discovery. Like any other global method for RNA identification [85],[86], our approach is likely to show certain biases, e.g., caused by cross-hybridization in the immunoprecipitation step, or from the limited ability of reverse transcriptase to deal with stable RNA structures in cDNA synthesis, and these will need to be studied in more detail. However, it is clear that deep sequencing resolved the termini of many expressed and/or Hfq-bound sRNAs at basepair resolution (Figure 5), which has not been achieved by other methods.

The combination of HTPS of co-immunoprecipitated sRNAs and mRNAs with transcriptomics partly explains how Hfq acts as a pleiotropic regulator of *Salmonella* gene expression. Transcriptome analysis under two different growth conditions suggests that Hfq regulates the expression of nearly a fifth of all *Salmonella* genes. This proportion of Hfq-dependent genes is similar to *Pseudomonas aeruginosa* (∼15% of all genes; [87]), but bigger than for *E. coli* (6.3%; [42]), or *Vibrio cholerae* (5.6%; [30]). However, the different growth conditions and scoring parameters used for these other organisms preclude a direct comparison with our *Salmonella* data. Nonetheless, the strong impact of Hfq on the σS and σE stress regulons that we observed is consistent with the findings in *E. coli* [42] and in part in *V. cholerae* (σE; [30]), and expands the previous work on *Salmonella* σS and σE regulated genes [34],[43],[44],[88],[89],[90],[91] to a global level. Importantly, our combined transcriptomic and coIP data revealed that Hfq exerts a direct role in gene expression through the control of specific check-points in other well-defined transcriptional regulons, such as HilD in the SPI-1 virulence regulon, and FlhD_2_C_2_ in the flagellar gene expression cascade.

Transcriptomic profiling by itself is clearly unable to differentiate between transcriptional and post-transcriptional effects of Hfq. In contrast, enrichment of a regulated mRNA in the Hfq library has successfully hinted at post-transcriptional regulation by sRNAs. For example, the observation of OmpX overproduction in *Salmonella* Δ*hfq,* combined with *ompX* mRNA enrichment by Hfq coIP in *E. coli* [17], led to the prediction that OmpX synthesis is repressed by an Hfq-dependent antisense sRNA; this sRNA was subsequently identified as CyaR in *Salmonella* [63]. Tables 2 and 3 confirm that both *ompX* mRNA and CyaR strongly associate with *Salmonella* Hfq (22.8-fold and 21.2-fold enrichment, respectively). Our current data set comprises several hundred such candidate mRNAs (Table S4); this catalogue contains many experimentally confirmed targets of *Salmonella* sRNAs, e.g., the *dppA, fadL*, *ompD,* or *oppA* mRNAs [34],[46],[58],[59]. Integrating the score for Hfq-association deduced from our experiments, and–where applicable–from the available *E. coli* data [17] into available algorithms such as TargetRNA [92] could significantly improve target predictions for the large class of Hfq-dependent sRNAs.

Such predictions bring new understanding to the pleiotropic phenotypes caused by the absence of Hfq in *Salmonella* [32]. The fact that the *Salmonella hfq* mutant is attenuated for virulence can now be explained by the requirement of Hfq for the expression of all but one key pathogenicity islands of *Salmonella* (SPI-3). In the SPI-1 invasion gene island, HilD acts at the top of a transcription factor cascade to activate SPI-1 genes, and to mediate secretion of effector proteins by the SPI-1 type III secretion system (reviewed in [67],[93]). The levels of *hilD* mRNA were sevenfold reduced in Δ*hfq*, but the unchanged activity of a *hilD* promoter fusion in this background (unpublished data) argues against direct transcriptional control by Hfq. Rather, the 7.5-fold enrichment of *hilD* cDNAs by Hfq coIP (Table S4) suggests that *hilD* is post-transcriptionally activated in a Hfq-dependent process, presumably involving an unknown sRNA. Our demonstration that SPI-1 virulence factor secretion is fully restored by HilD overproduction in Δ*hfq* raises the exciting possibility that post-transcriptional *hilD* activation could be key event in *Salmonella* invasion of epithelial cells.

We expect Hfq to have further roles in SPI-1 expression since the protein seems to bind to many mRNAs encoded by this pathogenicity island (Figures 4 and S4). Interestingly, SPI-1 has a significantly higher AT content than the rest of the *S.* Typhimurium chromosome [40], predicting that SPI-1 mRNAs are AU-rich. Coincidently, Hfq primarily recognizes AU-rich single-stranded regions in RNAs [12],[94],[95],[96]. This type of sequence is also recognized by the major endoribonuclease, RNase E, and Hfq has been shown to protect certain RNAs by competitive binding to RNase E sites [97],[98]. It is tempting to speculate that Hfq could reduce the impact of DNA from foreign sources by controlling expression of newly acquired AT-rich genes at the RNA level, similar to the role of the H-NS DNA-binding protein in controlling such genes at the DNA level [99],[100],[101].

Collectively, the present study provides the first picture of the impact of Hfq on *Salmonella* gene expression at both the transcriptional and post-transcriptional level. We believe that more detailed inspection of this freely available data set, in particular of the remaining ∼60% of the chromosome that remains to be fully analyzed, as well as sampling under different growth conditions, will expand the gamut of *Salmonella* small mRNA and noncoding RNA genes. In addition, the available data sets should help to discover whether Hfq controls the expression of *cis*-antisense sRNAs that overlap with mRNA coding regions [54], or whether certain *Salmonella* tRNAs are selectively associated with this protein [22],[23].

Bacterial genomes encode a large number of RNA binding proteins [102], including globally acting proteins such as the CsrA/RsmA [48] and Csp families [103]. Our generic method will identify the RNA targets of these proteins in any genetically tractable bacterium.

## Materials and Methods

### Bacterial Strains, Plasmids, and Oligodeoxynucleotides

The *Salmonella enterica* serovar Typhimurium strains used in this study were: JVS-0255 (Δ*hfq*::Cm^R^, [32]), JVS-1338 (*hfq* ^FLAG^, [46]), and the isogenic wild-type strain SL1344 [104]. Plasmid pKP8-35 [59] served as a pBAD control plasmid. The SPI-1 transcription factor, HilA, was expressed from pCH-112 [105], and HilD from plasmid pAS-0045 (which carries a *hilD* PCR fragment obtained with primer pair JVO-686/-687 amplified from *Salmonella* DNA, inserted into plasmid pLS-119 [106] by *Nco*I/*EcoR*I cloning). The FlhDC expression plasmid, pAS-0081, was constructed by inserting a PCR fragment obtained with primers JVS-2152/-2153 into plasmid pBAD/*Myc*-His A (Invitrogen) by *Nco*I/*Xho*I cloning. All cloning procedures where carried out in *E. coli* strain Top10 (Invitrogen). Table S6 lists the sequences of oligodeoxynucleotides used in this study for cloning and T7 transcript generation.

### Bacterial Growth and L-arabinose Induction

Growth in Lennox (L) broth (220 rpm, 37°C) or on L-plates at 37°C was used throughout this study. Antibiotics (where appropriate) were used at the following concentrations: 50 µg/ml ampicillin, 30 µg/ml chloramphenicol. For early stationary phase (ESP) cultures, 30 ml L-broth in 100 ml flasks were inoculated 1/100 from overnight cultures and incubated at 37°C, 220 rpm to an optical density of 2. For SPI-1 induced cultures, 5 ml L-broth containing NaCl (final concentration 0.3 M) was inoculated from single colonies; incubation was carried out for 12 hours at 37°C, 220 rpm in tightly closed 15 ml Falcon tubes. For SPI-2 induced cultures, 70 ml SPI-2 medium [107] in 250 ml flasks were inoculated 1/100 from overnight cultures grown in the same medium. Bacteria were grown at 37°C, 220 rpm until the culture reached an OD of 0.3. For HilA, HilD, and FlhDC expression from pBAD-derived plasmids, growth media were supplemented with 0.1% L-arabinose.

### Transcriptomic Experiments

Strain SL1344 and JVS-0255 (Δ*hfq*) were grown in L-broth either to an OD_600_ of 2 (ESP aerobic growth), or for 12 hours under SPI-1 inducing conditions. RNA extraction and data generation were carried out as described with SALSA microarrays [59]. The complete dataset is available at GEO under accession number GSE8985.

### SDS PAGE and Western Blot for Protein Quantification

Proteins were resolved by SDS PAGE (12% gels). For Coomassie stain or Western analysis, proteins equivalent to 0.1 OD or 0.05 OD, respectively, were loaded per lane. For FliC detection, strains SL1344 and JVS-0255 carrying the indicated plasmids were grown to an OD of 1, and induced with L-arabinose. Growth continued for one hour, and whole cell and secreted protein fractions were analyzed as described in [32]. FliC was detected using a monoclonal FliC antibody (BioLegend).

### RNA Isolation and Northern Blot Analysis

RNA was prepared by hot phenol extraction [108], followed by DNase I treatment. After separation on 5% polyacrylamide (PAA) gels containing 8.3 M Urea, or agarose gels, respectively, RNA was transferred onto Hybond-XL membrane (Amersham). 5 or 10 µg (PAA gels) or 20 µg (agarose gels) RNA was loaded per sample. For detection of new transcripts γ-ATP end-labeled oligodeoxyribonucleotides were used (see Table S7).

### Gel Mobility Shift Assay of *In Vitro* RNA

DNA templates carrying a T7 promoter sequence were generated by PCR using genomic DNA and primers as listed in Table S6. For *dppA* oligonucleotides JVO-1034/1035 (the fragment covers the *dppA* region from positions −163 to +73 relative to the start codon) were used. For the PCR of the intergenic region of *glmUS* primer JVO-2471/2472 were used, resulting in a product starting 38 nucleotides upstream of the *glmU* stop codon and extending to nucleotide 113 in the intergenic region. For *flhD*, oligonucleotides JVO-2284/-2285 were used, to yield a fragment that covers *flhD* from position −59 to +38 relative to the start codon. The *hilD* fragment (oligonucleotides JVO-2286/-2287) spans region +400 to +600 relative to the start codon.

*In vitro* transcription was performed using the MEGAscript High Yield Transcription Kit (Ambion, #1333), followed by DNase I digestion (1 unit, 15 min, 37°C). Following extraction with phenol:chloroform:isopropanol (25∶24∶1 v/v), the RNA was precipitated overnight at -20°C with 1 vol of isopropanol. RNA integrity was checked on a denaturing polyacrylamide gel. RNA was 5′ end-labeled and purified as described in [59].

Gel mobility shift assays were carried out as described in [32]. In brief, labeled RNA was used in 10 µl reactions at a final concentration of 4 nM. Hfq was added to a final concentration in the range of 1.25 to 150 nM of the hexamer. After incubation for 10 min at 37°C complexes were separated on 6% native PAA gels at 4°C. Signals were detected with a Fuji PhosphorImager.

### coIP and Sequence Analysis

Strains SL1344 and JVS-1338 (*hfq* ^FLAG^) were grown in L-broth under normal aeration at 37°C to ESP. Co-immunoprecipitation was carried out using the protocol published in [46]. For pyrosequencing and coIP-on-Chip experiments, samples of two independent pull down experiments were used. cDNA cloning and pyrosequencing was performed as described for the identification of eukaryotic microRNA [109] but omitting size-fractionation of RNA prior to cDNA synthesis. Microarrays used for the coIP-on-Chip experiments were designed and produced by Oxford Gene Technology (Kidlington, UK). They consist of 21,939 60-mer oligonucleotides tiled throughout the *S.* Typhimurium SL1344 NCTC13347 genome and 636 control oligonucleotides. The SL1344 sequence was obtained from the Sanger Institute (Hinxton, UK) website (http://www.sanger.ac.uk/Projects/Salmonella/). As this genome is not yet fully annotated, the oligonucleotides were associated with corresponding *S*. Typhimurium LT2 genes or intergenic regions, if conserved in both organisms. Full description of the microarray and protocols used for generating and analysing the data are associated with the dataset deposited in the GEO data repository (http://www.ncbi.nlm.nih.gov/geo/) under accession number GSE10149. For detailed description of data analysis using the Integrated Genome Browser see the Supplementary Text S1. In brief, cDNA reads ≥18 nt were mapped to the *Salmonella* chromosome and hits per nucleotide were calculated along the entire genome. To calculate *enrichment factors* for Hfq coIP, the Hfq cDNA number was divided by Control cDNA number at each position of the genome, following normalization to the total number of mapped reads. Upon upload of the *Salmonella* genome sequence and annotation from Genbank (NC_003197.fna and NC_003197.gff), the two graphs for each library were loaded into the Integrated Genome Browser (IGB) of Affymetrix (version IGB-4.56), which can be directly launched by Java Web Start at http://www.affymetrix.com/support/developer/tools/download_igb.affx or downloaded from http://genoviz.sourceforge.net/.

## Supporting Information

Figure S1 Expression levels of RpoE and RpoS in wild-type and Δ*hfq* cells. Samples were taken from wild-type and Δ*hfq* strains grown under standard conditions to early stationary phase (OD_600_ of 2) or for 12 hours under SPI-1 inducing conditions, respectively. (A) Analysis of mRNA level by real time PCR for *rpoE*, *degP*, and *rpoS* mRNA. (B) Whole cell proteins were separated by 12% SDS PAGE and sigma factors detected via Western blot. Expression levels of each protein were determined by densitometry and are given as a percentage of the wild-type level of expression below each gel.
(0.29 MB TIF)

Figure S2 Northern detection of Hfq bound mRNAs. Total RNA was isolated from *Salmonella* at OD_600_ of 2. Northern blots based on agarose gel for detection of long transcripts showing the detection of six mRNAs.
(1.29 MB TIF)

Figure S3 Expression levels of small peptide encoding mRNAs in *Salmonella*. RNA samples were either taken from wild-type or *hfq* mutant *Salmonella* at different growth stages (as in Figure 6 in the main manuscript), and probed for STnc250 and STnc570 over growth (A) or at early stationary phase (B).
(0.99 MB TIF)

Figure S4 Hfq binds significantly to a few but not all mRNAs of the SPI-1 and the flagellar regulon. Shown are all genes belonging to the SPI-1 and the flagellar regulon. The level of Hfq-dependent gene regulation is shown as fold-change below each gene (taken from the transcriptomic dataset; Table S1). Representation of cDNAs in pyrosequencing is indicated by different colours (green: 1–10 clones, turquoise: 11–100 clones, orange: 101–500 clones, magenta: ≥501 clones).
(0.41 MB TIF)

Figure S5 Expression of IstR-1 and IstR-2 in *Salmonella*. Northern analysis of istR transcripts. Total RNA was extracted from of *E. coli* K12 and *Salmonella* Typhimurium SL1344 cells grown to an OD_600_ of 2, exposed to Mitomycin C (0.5 µg/ml) for 30 min as described by [2]. Length is indicated according to marker sizes in nt. Full-length IstR-1 and IstR-2 are indicated by arrows.
(0.28 MB TIF)

Table S1 Deregulated genes in Δ*hfq* at ESP.
(0.95 MB DOC)

Table S2 Deregulated genes in Δ*hfq* after 12 hrs SPI-inducing conditions.
(0.21 MB DOC)

Table S3 Coverage of known and candidate *Salmonella* sRNA loci in pyrosequencing data.
(0.26 MB DOC)

Table S4 mRNAs in Hfq CoIP identified by ≥10 of 170,000 inserts in pyrosequencing data.
(0.81 MB DOC)

Table S5 Genes that were significantly enriched in coIP-on-Chip and were identified by pyrosequencing.
(0.32 MB DOC)

Table S6 Oligodeoxynucleotides used in this study.
(0.06 MB DOC)

Table S7 Oligodeoxynucleotides used for Northern detection.
(0.05 MB DOC)

Text S1 Supplementary material and methods.
(0.31 MB DOC)
