# Supplementary figures and images for: Deep Sequencing Analysis of Small Noncoding RNA and mRNA Targets of the Global Post-Transcriptional Regulator, Hfq
Source: PLoS Genet. 2008 Aug 22;4(8):e1000163. doi: 10.1371/journal.pgen.1000163 (PMC2515195; doi:10.1371/journal.pgen.1000163)

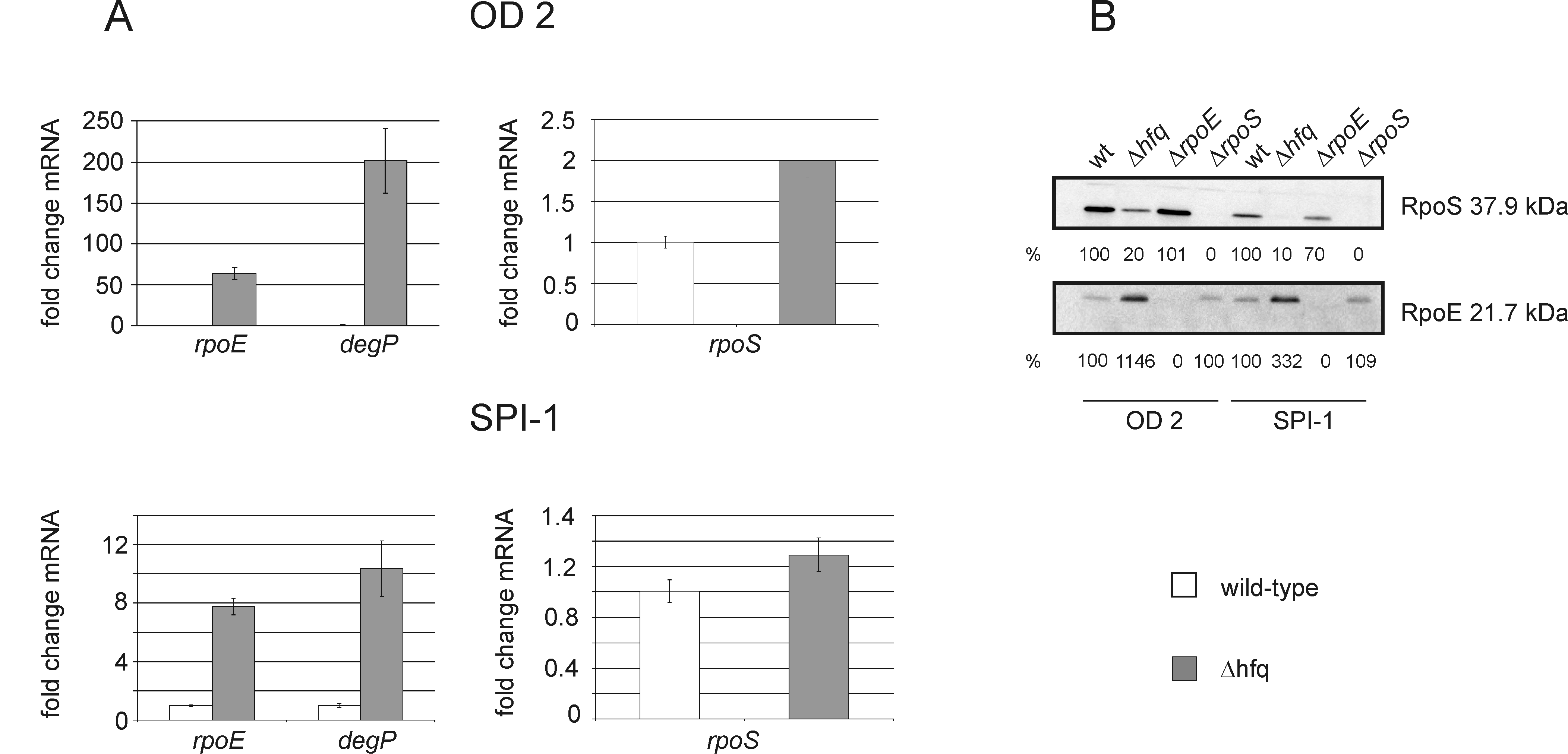

Supplement: Figure S1 — Expression levels of RpoE and RpoS in wild-type and Δhfq cells. Samples were taken from wild-type and Δhfq strains grown under standard conditions to early stationary phase (OD600 of 2) or for 12 hours under SPI-1 inducing conditions, respectively. (A) Analysis of mRNA level by real time PCR for rpoE, degP, and rpoS mRNA. (B) Whole cell proteins were separated by 12% SDS PAGE and sigma factors detected via Western blot. Expression levels of each protein were determined by densitometry and are given as a percentage of the wild-type level of expression below each gel. (0.29 MB TIF) [file pgen.1000163.s001.tif]

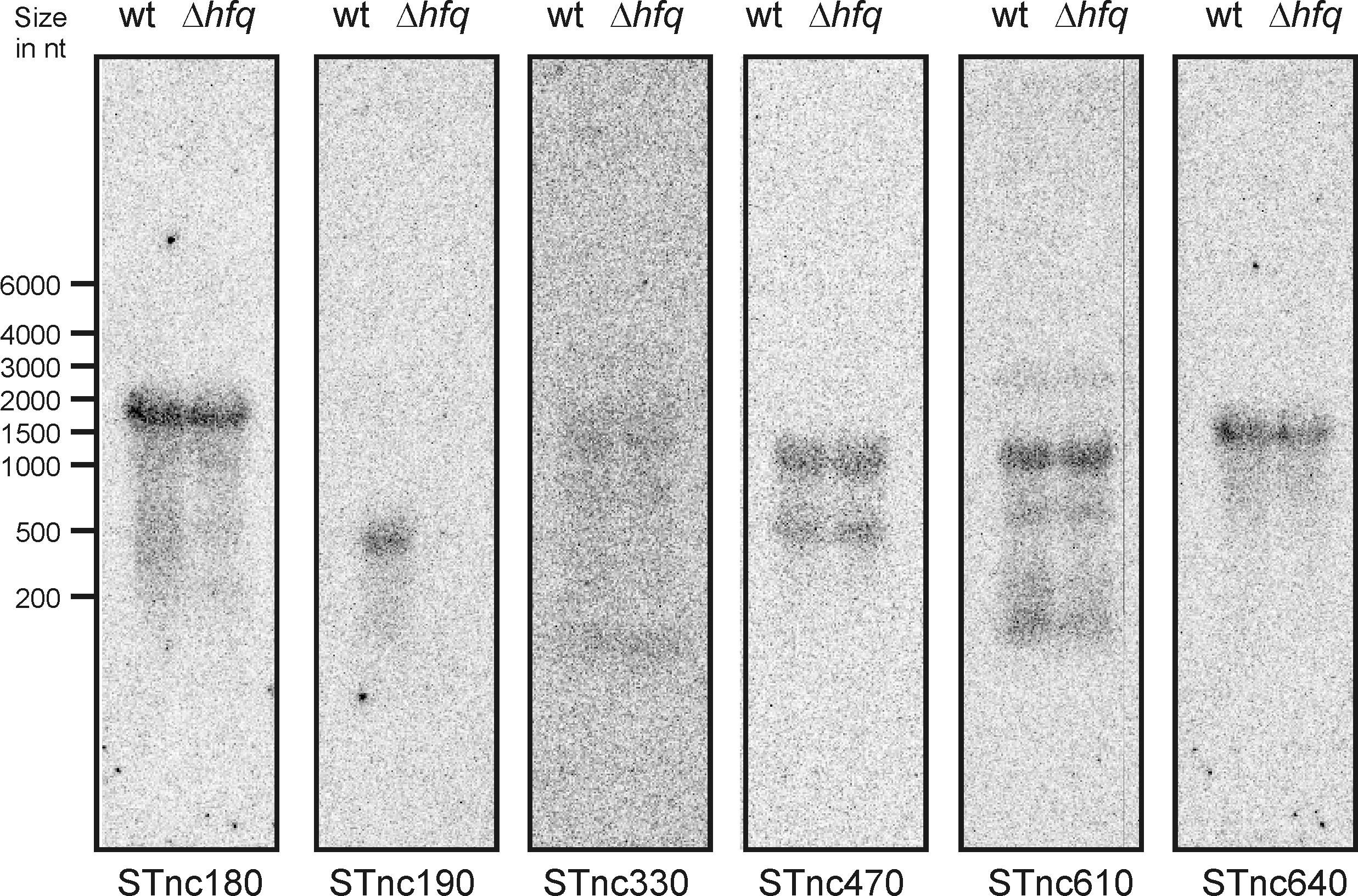

Supplement: Figure S2 — Northern detection of Hfq bound mRNAs. Total RNA was isolated from Salmonella at OD600 of 2. Northern blots based on agarose gel for detection of long transcripts showing the detection of six mRNAs. (1.29 MB TIF) [file pgen.1000163.s002.tif]

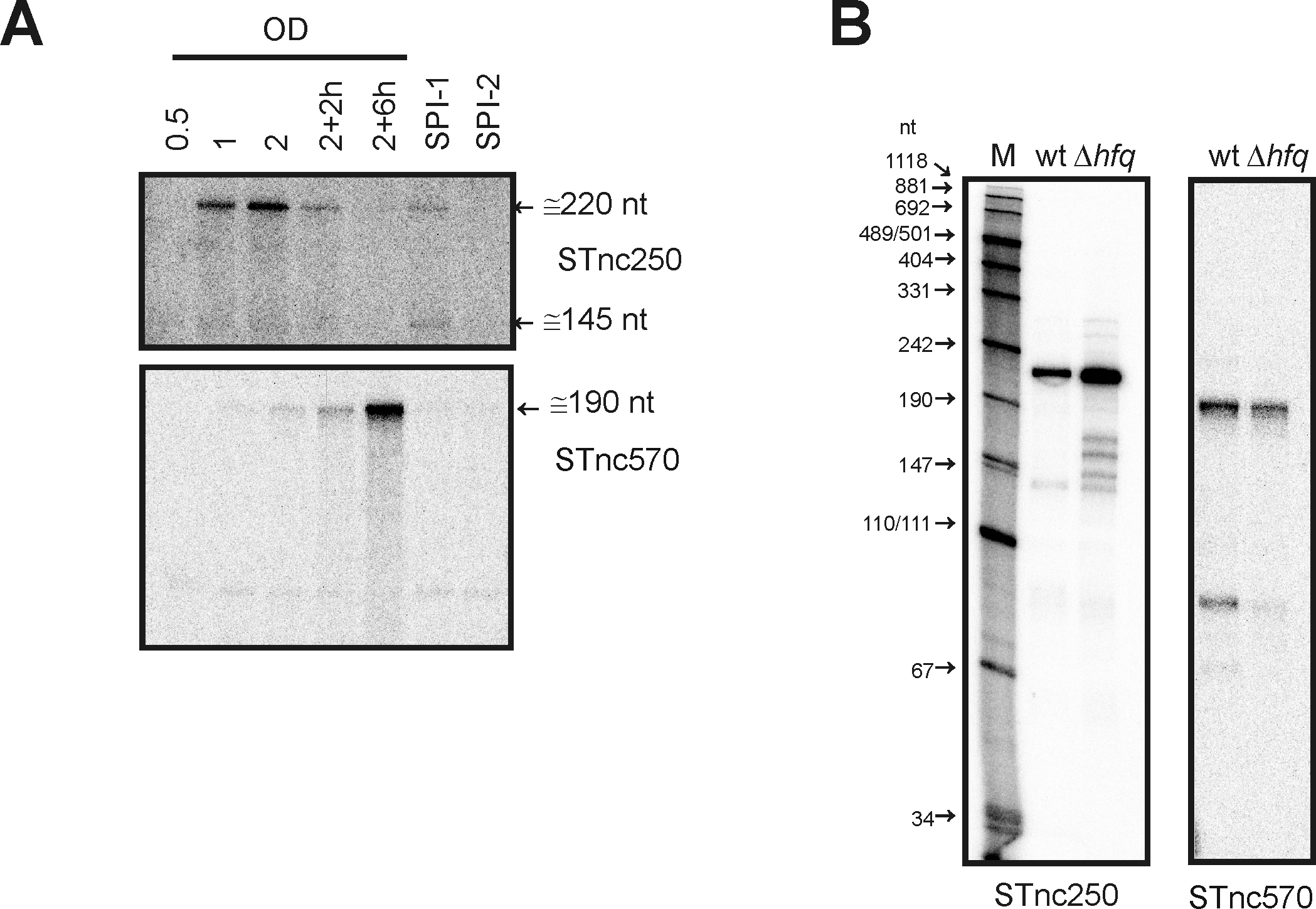

Supplement: Figure S3 — Expression levels of small peptide encoding mRNAs in Salmonella. RNA samples were either taken from wild-type or hfq mutant Salmonella at different growth stages (as in Figure 6 in the main manuscript), and probed for STnc250 and STnc570 over growth (A) or at early stationary phase (B). (0.99 MB TIF) [file pgen.1000163.s003.tif]

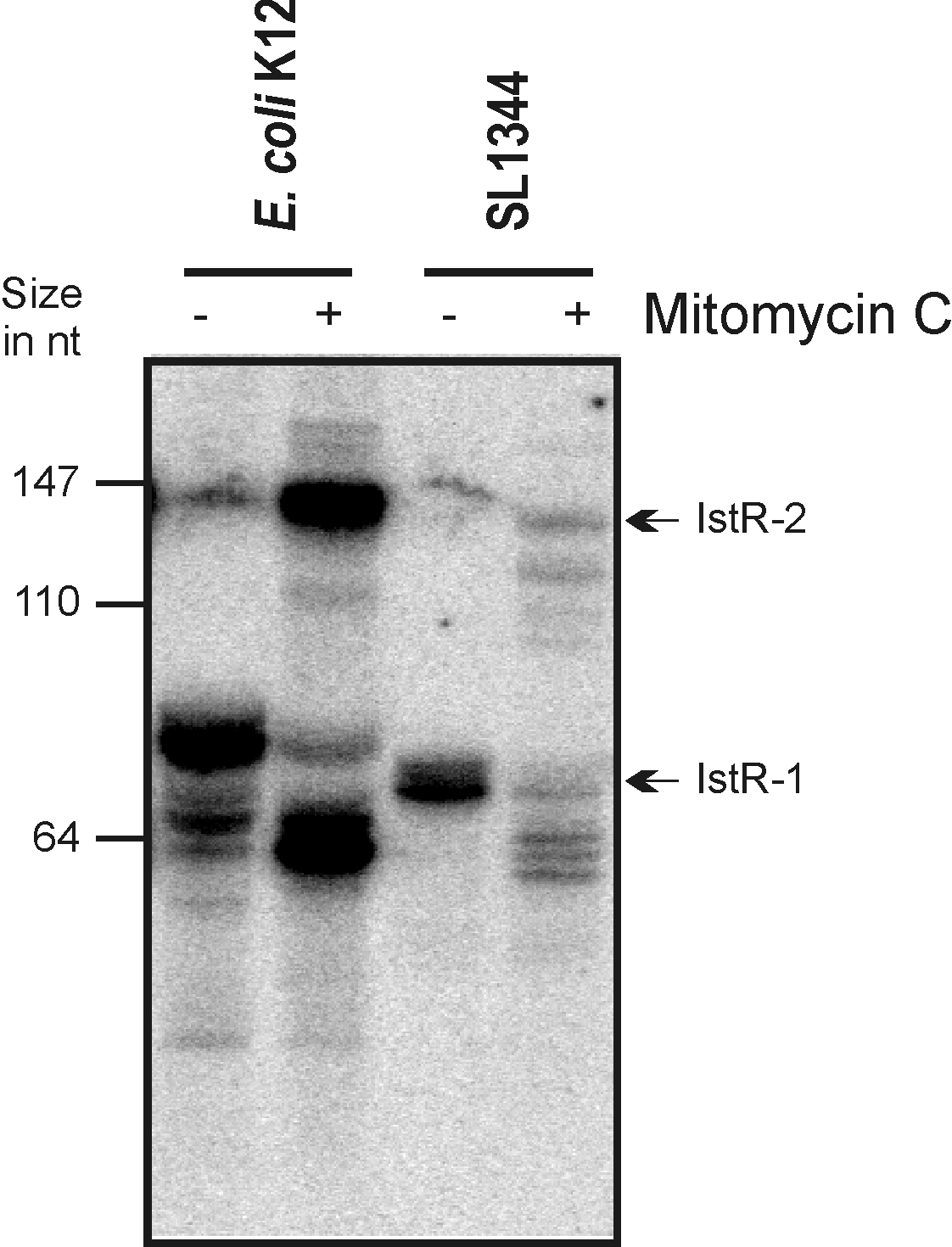

Supplement: Figure S5 — Expression of IstR-1 and IstR-2 in Salmonella. Northern analysis of istR transcripts. Total RNA was extracted from of E. coli K12 and Salmonella Typhimurium SL1344 cells grown to an OD600 of 2, exposed to Mitomycin C (0.5 µg/ml) for 30 min as described by [2]. Length is indicated according to marker sizes in nt. Full-length IstR-1 and IstR-2 are indicated by arrows. (0.28 MB TIF) [file pgen.1000163.s005.tif]
