# Supplementary material for: Deep Sequencing Analysis of Small Noncoding RNA and mRNA Targets of the Global Post-Transcriptional Regulator, Hfq
Source: PLoS Genet. 2008 Aug 22;4(8):e1000163. doi: 10.1371/journal.pgen.1000163 (PMC2515195; doi:10.1371/journal.pgen.1000163)
Supplement: Table S1 — Deregulated genes in Δhfq at ESP. (0.95 MB DOC) [file pgen.1000163.s006.doc]

**Table S1: Deregulated genes in Δ*hfq*** at ESP

| Gene name^a^ | Fold change | Product^b^ | 2D-analysis^c^ | HGT^d^ | coIP on Chip | cDNA coverage |
| --- | --- | --- | --- | --- | --- | --- |
| ybfM | 231.48 | putative outer membrane protein | X |  | X | X |
| ybfN | 179.86 | putative lipoprotein |  |  |  | X |
| napC | 48.08 | periplasmic nitrate reductase, cytochrome c-type protein |  |  |  |  |
| htrA | 41.15 | periplasmic serine protease Do, heat shock protein | X |  |  |  |
| napG | 39.68 | ferredoxin-type protein: electron transfer |  |  |  |  |
| napH | 35.84 | ferredoxin-type protein: electron transfer |  |  |  |  |
| napF | 29.5 | ferredoxin-type protein: electron transfer |  |  |  | X |
| rseA | 27.32 | anti sigma E (sigma 24) factor, negative regulator |  |  |  |  |
| yraP | 24.75 | paral putative periplasmic protein | X |  |  |  |
| napB | 19.12 | periplasmic nitrate reductase, small subunit, cytochrome C550, in complex with NapA |  |  |  |  |
| aphA | 18.55 | non-specific acid phosphatase/phosphotransferase, class B | X |  | X | X |
| narP | 18.42 | response regulator in two-component regulatory system with NarQ (or NarX) |  |  | X | X |
| yfaZ | 17.7 | putative inner membrane protein |  |  | X |  |
| yhjW | 17.18 | putative membrane-associated, metal-dependent hydrolase |  |  | X |  |
| napD | 17.04 | periplasmic nitrate reductase |  |  |  |  |
| cysA | 15.48 | ABC superfamily (atp_bind), sulfate permease A protein; chromate resistance |  |  |  |  |
| STM1253 | 15.41 | putative inner membrane protein |  | X |  |  |
| ycbK | 14.22 | putative outer membrane protein |  |  |  |  |
| nrfA | 13.64 | nitrite reductase periplasmic cytochrome c(552) |  |  |  |  |
| rpoE | 12.76 | sigma E (sigma 24 ) factor of RNA polymerase, response to periplasmic stress |  |  |  | X |
| tpx | 12.41 | thiol peroxidase | X |  |  | X |
| ygiM | 11.93 | putative SH3 domain protein |  |  |  |  |
| yraO | 11.78 | putative phosphoheptose isomerase |  |  |  |  |
| nrfD | 11.55 | putative nitrate reductase, formate dependent |  |  |  |  |
| yfiO | 10.88 | putative lipoprotein |  |  |  |  |
| cysD | 10.65 | ATP-sulfurylase, subunit 1 (ATP:sulfate adenylyltransferase) |  |  |  |  |
| yiaD | 10.55 | putative outer membrane lipoprotein | X |  |  |  |
| glnH | 10.27 | ABC superfamily (bind_prot), glutamine high-affinity transporter | X |  | X |  |
| rseB | 9.9 | anti sigma E (sigma 24) factor, negative regulator |  |  |  | X |
| nrfB | 9.26 | formate-dependent nitrite reductase; a penta-haeme cytochrome c |  |  |  |  |
| phnA | 8.93 | putative alkylphosphonate uptake protein in phosphonate metabolism |  |  | X |  |
| yggN | 8.93 | putative periplasmic protein |  |  | X | X |
| cysW | 8 | ABC superfamily (membrane), thiosulfate permease W protein |  |  |  |  |
| citA | 7.87 | citrate-proton symporter |  |  |  |  |
| cysP | 7.69 | ABC superfamily (bind_prot), thiosulfate transport protein | X |  |  |  |
| ydjN | 7.63 | part of a kinase, putative domain shared with transporter |  |  |  |  |
| ccmG | 7.58 | heme lyase disulfide oxidoreductase, cytocyhrome c-type biogenesis |  | X |  |  |
| ygjU | 7.58 | putative dicarboxylate permease |  |  | X | X |
| yaeT | 7.25 | putative outer membrane antigen | X |  |  | X |
| ansB | 7.09 | periplasmic L-asparaginase II |  |  | X | X |
| rfaK | 7.04 | putative hexose transferase, lipopolysaccharide core biosynthesis |  | X | X |  |
| dctA | 6.76 | DAACS family, C4-dicarboxylic acids transport protein |  |  |  | X |
| dppA | 6.49 | ABC superfamily (peri_perm), dipeptide transport protein | X |  | X | X |
| oppA | 6.45 | ABC superfamily (periplasm), oligopeptide transport protein with chaperone properties | X |  | X | X |
| STM2447 | 6.37 | putative outer membrane lipoprotein |  |  |  |  |
| lrhA | 6.33 | NADH dehydrogenase transcriptional repressor (LysR family) |  |  | X | X |
| ccmF | 6.21 | cytochrome c-type biogenesis protein |  | X |  |  |
| cysI | 6.21 | sulfite reductase, alpha subunit, NADPH dependent |  |  |  |  |
| gcvH | 6.06 | glycine cleavage complex protein H, carrier of aminomethyl moiety via covalently bound lipoyl cofactor |  |  |  | X |
| ycbL | 5.85 | putative Metallo-beta-lactamase |  |  |  |  |
| yhjG | 5.81 | putative inner membrane protein |  |  |  |  |
| stdA | 5.78 | putative fimbrial-like protein |  | X | X |  |
| STM1539 | 5.75 | putative hydrogenase-1 small subunit |  |  |  |  |
| rplD | 5.68 | 50S ribosomal subunit protein L4, regulates expression of S10 operon |  |  |  | X |
| sdaC | 5.65 | putative HAAAP family, serine transport protein |  |  | X |  |
| nlpB | 5.59 | lipoprotein-34 | X |  |  |  |
| hlpA | 5.52 | histone-like protein, located in outer membrane |  |  |  |  |
| STM1255 | 5.38 | putative ABC transporter periplasmic binding protein |  |  |  |  |
| rpsG | 5.35 | 30S ribosomal subunit protein S7, initiates assembly |  |  |  |  |
| yhjJ | 5.35 | putative Zn-dependent peptidase |  |  |  |  |
| rplW | 5.05 | 50S ribosomal subunit protein L23 |  |  |  |  |
| STM4351 | 5.05 | putative arginine-binding periplasmic protein |  |  |  |  |
| nrfC | 5 | putative nitrite reductase; formate-dependent, Fe-S centers |  |  |  |  |
| rplB | 4.83 | 50S ribosomal subunit protein L2 |  |  |  |  |
| STM4466 | 4.76 | putative carbamate kinase |  |  |  |  |
| cysK | 4.69 | subunit of cysteine synthase A and O-acetylserine sulfhydrolase A |  |  |  |  |
| yfgD | 4.61 | putative arsenate reductase |  |  |  |  |
| mglB | 4.52 | ABC superfamily (peri_perm), galactose transport protein | X |  |  | X |
| nrfE | 4.52 | formate-dependent nitrite reductase; involved in attachment of haem c to cytochrome c552 |  |  |  |  |
| STM4195 | 4.52 | putative Na+-dependent transporter |  |  |  |  |
| ushA | 4.52 | UDP-sugar hydrolase 5'-nucleotidase |  |  | X | X |
| yijD | 4.41 | putative inner membrane protein |  |  |  |  |
| rseC | 4.39 | regulator of sigma E (sigma 24) factor |  |  |  |  |
| STM4465 | 4.31 | putative ornithine carbamoyltransferase |  |  |  |  |
| cspD | 4.27 | similar to CspA but not cold shock induced | X |  |  | X |
| cutC | 4.24 | copper homeostasis protein |  |  | X | X |
| STM0509 | 4.24 | putative outer membrane protein |  |  |  |  |
| STM0719 | 4.18 | putative UDP-galactopyranose mutase |  | X |  |  |
| rbsB | 4.17 | ABC superfamily (peri_perm), D-ribose transport protein | X |  | X | X |
| gdhA | 4.1 | glutamate dehydrogenase, NADP-specific |  |  |  |  |
| yabJ | 4.07 | putative ABC-transport protein |  |  | X |  |
| gcvT | 4.03 | glycine cleavage complex protein T, aminomethyltransferase, tetrahydrofolate-dependent |  |  |  | X |
| cysN | 4 | ATP-sulfurylase, subunit 1 (ATP:sulfate adenylyltransferase) |  |  |  |  |
| hflK | 3.94 | with HflC, part of modulator for protease specific for FtsH phage lambda cII repressor |  |  |  |  |
| STM1747 | 3.94 | putative inner membrane protein |  |  | X |  |
| cycA | 3.89 | APC family, D-alanine/D-serine/glycine transport protein |  |  |  |  |
| pal | 3.88 | tol protein required for outer membrane integrity, uptake of group A colicins, and translocation of phage DNA to cytoplasm | X |  |  | X |
| ybhQ | 3.77 | putative inner membrane protein |  |  |  | X |
| glpT | 3.76 | MFS family, sn-glycerol-3-phosphate transport protein |  |  |  | X |
| purC | 3.75 | phosphoribosylaminoimidazole-succinocarboxamide synthetase (SAICAR synthetase) |  |  |  |  |
| STM1538 | 3.73 | putative hydrogenase-1 large subunit |  |  |  |  |
| rpsC | 3.72 | 30S ribosomal subunit protein S3 |  |  |  | X |
| rplV | 3.7 | 50S ribosomal subunit protein L22 |  |  |  |  |
| STM4276 | 3.65 | putative cytoplasmic protein |  |  |  |  |
| ddg | 3.64 | cold shock-induced palmitoleoyl transferase |  |  |  |  |
| glpQ | 3.64 | glycerophosphodiester phosphodiesterase, periplasmic | X |  |  | X |
| livK | 3.62 | ABC superfamily (bind_prot), branched-chain amino acid transporter, high-affinity |  |  |  |  |
| tsx | 3.62 | nucleoside channel; receptor of phage T6 and colicin K |  |  |  |  |
| rplC | 3.6 | 50S ribosomal subunit protein L3 | X |  |  | X |
| ndk | 3.58 | nucleoside diphosphate kinase |  |  |  | X |
| PSLT103 | 3.58 |  |  |  |  |  |
| gcvP | 3.57 | glycine cleavage complex protein P, glycine decarboxylase |  |  |  | X |
| hflC | 3.55 | with HflK, part of modulator for protease specific for FtsH phage lambda cII repressor |  |  |  |  |
| STM2494 | 3.55 | putative inner membrane or exported | X |  |  | X |
| rplP | 3.52 | 50S ribosomal subunit protein L16 |  |  |  |  |
| STM3169 | 3.5 | putative dicarboxylate-binding periplasmic protein |  |  |  |  |
| tig | 3.5 | peptidyl-prolyl cis/trans isomerase, trigger factor; a molecular chaperone involved in cell division |  |  |  |  |
| cysC | 3.48 | adenosine 5'-phosphosulfate kinase |  |  |  |  |
| cpxP | 3.45 | periplasmic repressor of cpx regulon by interaction with CpxA, rescue from transitory stresses |  |  | X | X |
| cca | 3.42 | tRNA nucleotidyl transferase |  |  |  |  |
| yfgL | 3.4 | putative serine/threonine protein kinase |  |  |  | X |
| rpsS | 3.39 | 30S ribosomal subunit protein S19 |  |  |  | X |
| STM2238 | 3.39 | putative phage protein |  |  | X |  |
| rfbP | 3.36 | LPS side chain defect: bifunctional enzyme: undecaprenol-phosphate galactosephosphotransferase, and O-antigen transfer |  | X | X | X |
| ibpB | 3.33 | small heat shock protein |  |  |  | X |
| ptr | 3.32 | protease III | X |  |  |  |
| ytfJ | 3.32 | putative transcriptional regulator |  |  | X |  |
| PSLT066 | 3.31 |  |  |  |  |  |
| ompF | 3.29 | outer membrane protein 1a (ia;b;f), porin | X |  |  | X |
| STM3170 | 3.28 | putative inner membrane protein |  |  |  |  |
| STM4467 | 3.26 | putative arginine deiminase |  |  |  |  |
| ycfS | 3.26 | putative periplasmic protein |  |  |  |  |
| mreB | 3.23 | rod shape-determining protein; HSP70 class molecular chaperones involved in cell morphogenesis |  |  |  |  |
| mod | 3.18 | DNA methylase; restriction system |  |  |  |  |
| mgtA | 3.16 | P-type ATPase, Mg2+ ATPase transporter |  |  |  |  |
| mglA | 3.15 | ABC superfamily (atp_bind), galactose (methyl-galactoside) transport protein |  |  | X | X |
| slp | 3.14 | putative outer membrane protein |  |  |  |  |
| rplN | 3.12 | 50S ribosomal subunit protein L14 |  |  |  | X |
| oppB | 3.05 | ABC superfamily (membrane), oligopeptide transport protein |  |  |  | X |
| sixA | 3.04 | phosphohistidine phosphatase |  |  |  |  |
| fadD | 2.94 | acyl-CoA synthetase (long-chain-fatty-acid--CoA ligase) |  |  | X |  |
| pyrI | 2.94 | aspartate carbamoyltransferase, regulatory subunit (allosteric regulation) |  |  |  |  |
| ycfR | 2.94 | putative outer membrane protein |  |  |  |  |
| lrp | 2.92 | regulator for lrp regulon and high-affinity branched-chain amino acid transport system; mediator of of leucine response (AsnC family) |  |  | X | X |
| rplF | 2.87 | 50S ribosomal subunit protein L6 |  |  |  |  |
| STM2747 | 2.87 | putative cytoplasmic protein |  | X | X |  |
| mglC | 2.86 | ABC superfamily (membrane), methyl-galactoside transport protein |  |  | X |  |
| ydgH | 2.86 | putative periplasmic protein |  |  |  |  |
| STM2746 | 2.85 | putative Excinuclease ATPase subunit |  | X | X |  |
| rplX | 2.83 | 50S ribosomal subunit protein L24 |  |  |  |  |
| cysM | 2.82 | cysteine synthase B (O-acetylserine sulfhydrolase B) |  |  |  |  |
| rpmC | 2.82 | 50S ribosomal subunit protein L29 |  |  |  |  |
| STM0906 | 2.82 | Fels-1 prophage |  | X |  |  |
| glpF | 2.81 | MIP channel, glycerol diffusion |  |  | X | X |
| STM4305 | 2.79 | putative anaerobic dimethyl sulfoxide reductase, subunit A |  |  |  | X |
| STM1368 | 2.78 | putative Na+-dicarboxylate symporter |  |  |  |  |
| yfgM | 2.76 | putative inner membrane protein |  |  |  |  |
| agp | 2.69 | glucose-1-phosphatase |  |  |  |  |
| priB | 2.69 | primosomal replication protein N |  |  |  | X |
| pyrB | 2.68 | aspartate carbamoyltransferase, catalytic subunit |  |  |  |  |
| yaeL | 2.67 | putative membrane-associated Zn-dependent protease |  |  |  |  |
| STM1256 | 2.65 | putative ABC transporter |  |  |  |  |
| hflX | 2.64 | putative GTP-ase, together with HflCK possibly involved in phage lambda cII repressor stability |  |  | X | X |
| STM1250 | 2.64 | putative cytoplasmic protein |  | X |  |  |
| fabI | 2.62 | enoyl-[acyl-carrier-protein] reductase (NADH) |  |  |  |  |
| stdB | 2.62 | putative outer membrane usher protein |  |  |  |  |
| ytgA | 2.62 | putative inner membrane protein |  |  | X |  |
| rplE | 2.61 | 50S ribosomal subunit protein L5 |  |  |  | X |
| rpsA | 2.6 | 30S ribosomal subunit protein S1 |  |  |  |  |
| ybhC | 2.6 | putative pectinesterase |  |  |  |  |
| greA | 2.59 | transcription elongation factor, cleaves 3' nucleotide of paused mRNA | X |  |  |  |
| nhaB | 2.59 | NhaB family of transport protein, Na+/H+ antiporter, regulator of intracellular pH |  |  |  |  |
| lpxD | 2.58 | UDP-3-O-(3-hydroxymyristoyl)-glucosamine n-acyltransferase |  |  |  | X |
| potD | 2.56 | ABC superfamily (peri_perm), spermidine/putrescine transporter |  |  |  |  |
| tktA | 2.54 | transketolase 1 isozyme |  |  |  |  |
| fumA | 2.53 | fumarase A (fumarate hydratase class I), aerobic isozyme |  |  |  | X |
| serA | 2.52 | D-3-phosphoglycerate dehydrogenase |  |  |  |  |
| mdoH | 2.51 | membrane glycosyltransferase; synthesis of membrane-derived oligosaccharide (MDO)/synthesis of OPGs (osmoregulated periplasmic glucans) |  |  |  |  |
| hmpA | 2.48 | dihydropteridine reductase 2 and nitric oxide dioxygenase activity |  |  |  |  |
| tsf | 2.48 | protein chain elongation factor EF-Ts | X |  |  | X |
| serC | 2.46 | 3-phosphoserine aminotransferase / phosphohydroxythreonine transaminase |  |  |  | X |
| tolB | 2.46 | tol protein required for outer membrane integrity, uptake of group A colicins, and translocation of phage DNA to cytoplasm, may be part of multiprotein peptidoglycan recycling complex (Two domains) |  |  |  | X |
| rpmG | 2.44 | 50S ribosomal subunit protein L33 |  |  |  |  |
| sbp | 2.44 | ABC superfamily (bind_prot), sulfate transport protein |  |  |  |  |
| STM3127 | 2.44 | putative cytoplasmic protein |  |  |  |  |
| surA | 2.44 | peptidyl-prolyl cis-trans isomerase, survival protein | X |  |  |  |
| hemN | 2.43 | O2-independent coproporphyrinogen III oxidase |  |  |  |  |
| rpsB | 2.43 | 30S ribosomal subunit protein S2 |  |  |  | X |
| rpsH | 2.43 | 30S ribosomal subunit protein S8, and regulator |  |  |  | X |
| yfcB | 2.43 | putative methylase |  |  |  |  |
| STM4423 | 2.42 | putative AraC-type DNA-binding domain-containing protein |  |  |  |  |
| STM4424 | 2.42 | putative endonuclease |  |  |  |  |
| bacA | 2.4 | bacitracin resistance; possibly phosphorylates undecaprenol |  |  |  |  |
| fabB | 2.4 | 3-oxoacyl-[acyl-carrier-protein] synthase I |  |  |  | X |
| oppD | 2.4 | ABC superfamily (atp-binding), oligopeptide transport protein |  |  |  | X |
| ybhR | 2.4 | putative ABC superfamily (membrane) transport protein |  |  |  |  |
| btuB | 2.39 | outer membrane receptor for transport of vitamin B12, E colicins, and bacteriophage BF23 |  |  |  |  |
| kdgK | 2.39 | ketodeoxygluconokinase |  |  |  |  |
| rpsN | 2.39 | 30S ribosomal subunit protein S14 |  |  |  |  |
| htpX | 2.38 | heat shock protein, integral membrane protein |  |  |  |  |
| PSLT102 | 2.38 |  |  |  | X |  |
| plsB | 2.37 | glycerolphosphate acyltransferase activity |  |  |  |  |
| fusA | 2.36 | protein chain elongation factor EF-G, GTP-binding |  |  |  | X |
| engA | 2.35 | putative GTP-binding protein |  |  |  |  |
| pyrL | 2.34 | pyrBI operon leader peptide |  |  |  |  |
| rpoH | 2.33 | sigma H (sigma 32) factor of RNA polymerase; transcription of heat shock proteins induced by cytoplasmic stress |  |  |  | X |
| rpsL | 2.33 | 30S ribosomal subunit protein S12 |  |  |  |  |
| rpsD | 2.31 | 30S ribosomal subunit protein S4 | X |  |  | X |
| sbmA | 2.31 | putative ABC superfamily transporter |  |  | X | X |
| ttk | 2.3 | putative transcriptional regulator (TetR/ArcR family) |  |  | X |  |
| fepE | 2.29 | ferric enterobactin (enterochelin) transporter |  |  | X |  |
| STM2706 | 2.28 | Fels-2 prophage: similar to tail fiber protein in phage P2 |  |  |  |  |
| oafA | 2.26 | O-antigen five: acetylation of the O-antigen (LPS) |  |  | X |  |
| rpsQ | 2.26 | 30S ribosomal subunit protein S17 |  |  |  |  |
| sdaB | 2.26 | L-serine dehydratase (L-threonine deaminase 2) |  |  |  |  |
| rfbK | 2.25 | LPS side chain defect: phosphomannomutase |  | X | X | X |
| yijC | 2.25 | putative transcriptional repressor (TetR/AcrR family) |  |  |  | X |
| glyQ | 2.24 | glycine tRNA synthetase, alpha subunit |  |  |  |  |
| prsA | 2.24 | phosphoribosylpyrophosphate synthetase | X |  |  |  |
| psd | 2.24 | phosphatidylserine decarboxylase |  |  |  |  |
| dinI | 2.23 | DNA damage-inducible protein I, inhibits UmuD processing |  |  |  |  |
| rplR | 2.23 | 50S ribosomal subunit protein L18 |  |  |  |  |
| STM0908 | 2.23 | Fels-1 prophage |  |  |  |  |
| tgt | 2.23 | tRNA-guanine transglycosylase |  |  |  |  |
| trmA | 2.23 | tRNA (uracil-5-)-methyltransferase |  |  |  |  |
| yiiD | 2.23 | putative acetyltransferase |  |  |  |  |
| ydgR | 2.22 | putative POT family, peptide transport protein |  |  |  |  |
| res | 2.21 | DNA restriction (DNA helicase |  |  |  |  |
| tolQ | 2.21 | tol protein, membrane-spanning inner membrane proteins, required for outer membrane integrity, uptake of group A colicins, and translocation of phage DNA to cytoplasm |  |  |  |  |
| PSLT068 | 2.2 |  |  |  |  |  |
| STM2705 | 2.2 | Fels-2 prophage |  |  | X |  |
| rpsE | 2.19 | 30S ribosomal subunit protein S5 |  |  |  | X |
| STM1131 | 2.19 | putative outer membrane protein |  | X |  |  |
| yceH | 2.19 | putative cytoplasmic protein |  |  |  |  |
| yhgG | 2.19 | putative cytoplasmic protein |  |  |  |  |
| STM1530 | 2.18 | putative outer membrane protein |  |  | X |  |
| ushB | 2.17 | CDP-diacylglycerol phosphotidylhydrolase |  |  |  |  |
| glyA | 2.16 | serine hydroxymethyltransferase |  |  |  |  |
| glyS | 2.16 | glycine tRNA synthetase, beta subunit |  |  |  |  |
| mopB | 2.16 | chaperone Hsp10, affects cell division |  |  |  |  |
| rpmD | 2.16 | 50S ribosomal subunit protein L30 |  |  |  |  |
| trmD | 2.16 | tRNA (guanine-7-)-methyltransferase |  |  |  | X |
| STM4306 | 2.15 | putative anaerobic dimethyl sulfoxide reductase, subunit B |  |  |  |  |
| feoB | 2.14 | FeoB family, ferrous iron transport protein B |  |  |  |  |
| pyrE | 2.14 | orotate phosphoribosyltransferase |  |  |  |  |
| yidC | 2.14 | putative Preprotein translocase subunit YidC |  |  |  |  |
| hybC | 2.13 | hydrogenase-2, large subunit |  |  |  | X |
| STM1607 | 2.13 | putative outer membrane lipoprotein |  |  |  |  |
| cadB | 2.12 | APC family, lysine/cadaverine transport protein |  |  |  |  |
| gnd | 2.12 | gluconate-6-phosphate dehydrogenase, decarboxylating |  |  | X | X |
| rfbU | 2.12 | LPS side chain defect: mannosyl transferase |  | X | X | X |
| STM4307 | 2.12 | putative anaerobic dimethyl sulfoxide reductase, subunit C |  |  |  |  |
| nirC | 2.11 | FNT family, nitrite transport protein |  |  |  |  |
| yggT | 2.11 | putative integral membran resistance protein |  |  |  |  |
| yhcB | 2.11 | putative periplasmic protein |  |  |  | X |
| PSLT069 | 2.1 |  |  |  |  |  |
| STM1252 | 2.1 | putative cytoplasmic protein |  | X |  |  |
| STM2237 | 2.1 | putative inner membrane protein |  |  |  |  |
| STM2754 | 2.1 | putative hexulose 6 phosphate synthase |  |  | X |  |
| ydcG | 2.1 | paral putative periplasmic glucans biosynthesis protein |  |  |  |  |
| rplO | 2.09 | 50S ribosomal subunit protein L15 |  |  |  | X |
| yaiW | 2.09 | putative outer membrane lipoprotein |  |  |  |  |
| aroP | 2.08 | APC family, aromatic amino acid transporter |  |  |  |  |
| STM3604 | 2.08 | putative inner membrane protein |  |  |  | X |
| STM4464 | 2.07 | putative arginine repressor |  |  |  |  |
| yhcG | 2.07 | putative cytoplasmic protein |  |  |  |  |
| prpC | 2.06 | putative citrate synthase |  |  |  |  |
| rpmF | 2.06 | 50S ribosomal subunit protein L32 |  |  |  |  |
| yheO | 2.06 | putative regulator |  |  |  |  |
| asd | 2.05 | aspartate-semialdehyde dehydrogenase |  |  |  |  |
| kbl | 2.05 | 2-amino-3-ketobutyrate CoA ligase (glycine acetyltransferase) | X |  |  |  |
| STM0149 | 2.05 | putative permease of the Na+:galactoside symporter family |  |  |  |  |
| STM3259 | 2.05 | PTS family galactitol-specific enzyme IIB |  |  |  |  |
| STM4418 | 2.05 | sugar (and other) transporter |  |  |  |  |
| agsA | 2.04 | Molecular chaperone (small heat shock protein) Tomoyasu, T (2003) J. Bact. 185: 6331-9 |  | X |  |  |
| PSLT003 | 2.04 |  |  |  |  |  |
| STM1485 | 2.04 | acid shock protein |  |  |  |  |
| STM1540 | 2.04 | putative hydrolase |  |  |  |  |
| STM2236 | 2.04 | putative phage protein |  |  |  |  |
| STM2636 | 2.04 | Gifsy-1 prophage: similar to integrase in phage |  |  |  |  |
| STM2767 | 2.04 | putative Superfamily I DNA and RNA helicase |  | X | X | X |
| uraA | 2.04 | NCS2 family, uracil transport protein |  |  |  |  |
| sfbA | 2.03 | putative ABC-type transport system ATPase component/cell division protein |  |  | X |  |
| crp | 2.02 | catabolite activator protein (CAP), cyclic AMP receptor protein (CRP family) |  |  | X | X |
| maeB | 2.02 | paral putative transferase | X |  |  | X |
| ompD | 2.02 | new outer membrane protein; predicted bacterial porin |  |  |  | X |
| nirD | 2.01 | nitrite reductase, small subunit |  |  | X |  |
| mpl | 2 | UDP-N-acetylmuramate:L-alanyl-gamma-D-glutamyl- meso-diaminopimelate ligase |  |  |  | X |
| STM3633 | 2 | putative bacterial regulatory proteins, lacI family |  |  |  |  |
| rfaD | -2 | ADP-L-glycero-D-mannoheptose-6-epimerase |  |  |  | X |
| STM1324 | -2 | putative cytoplasmic protein |  |  |  | X |
| STM2905 | -2 | putative acetyltransferase |  | X |  |  |
| fhlA | -2.01 | formate hydrogen-lyase transcriptional activator for fdhF, hyc and hyp operons (EBP family) |  |  |  |  |
| fliQ | -2.01 | flagellar biosynthesis |  |  |  |  |
| gcd | -2.01 | glucose dehydrogenase |  |  |  |  |
| proW | -2.01 | ABC superfamily (membrane), glycine/betaine/proline transport protein |  |  |  |  |
| ptsG | -2.02 | Sugar Specific PTS family, glucose-specific IIBCcomponent |  |  | X | X |
| fliE | -2.03 | putative Flagellar hook-basal body protein |  |  |  |  |
| STM0856 | -2.03 | putative electron transfer flavoprotein alpha subunit |  | X |  |  |
| yneC | -2.03 | putative inner membrane protein |  | X |  |  |
| yjjV | -2.04 | putative hydrolase |  |  |  |  |
| PSLT023 | -2.05 |  |  |  |  |  |
| yhjD | -2.05 | putative tRNA-processing ribonuclease |  |  |  |  |
| yncD | -2.05 | paral putative outer membrane receptor |  |  |  |  |
| celA | -2.06 | PTS family, sugar specific enzyme IIB for cellobiose, arbutin, and salicin |  | X | X | X |
| ybhL | -2.06 | putative permease |  |  |  | X |
| celD | -2.07 | transcriptional repressor of cel operon (AraC/XylS family) |  |  |  |  |
| ddlB | -2.07 | D-alanine-D-alanine ligase B, affects cell division |  |  |  | X |
| pagD | -2.08 | PhoP regulated |  | X |  |  |
| rfaF | -2.08 | ADP-heptose; LPS heptosyltransferase 1 |  |  |  | X |
| STM1630 | -2.08 | putative inner membrane protein |  | X |  |  |
| STM2614 | -2.08 | Gifsy-1 prophage |  |  |  |  |
| ynbE | -2.08 | putative outer membrane lipoprotein |  |  |  |  |
| ynfD | -2.08 | putative outer membrane protein |  |  |  |  |
| ldhA | -2.09 | fermentative D-lactate dehydrogenase, NAD-dependent |  |  |  |  |
| ydfZ | -2.09 | putative cytoplasmic protein |  |  |  |  |
| yjjU | -2.09 | putative phosphoesterase |  |  |  | X |
| ynhG | -2.09 | putative LysM domain |  |  |  |  |
| qor | -2.1 | quinone oxidoreductase, NADPH dependent |  |  |  |  |
| csgA | -2.11 | curlin major subunit, coiled surface structures; cryptic |  | X |  |  |
| STM1859 | -2.11 | putative cytoplasmic protein |  |  |  |  |
| STM1939 | -2.11 | putative glucose-6-phosphate dehydrogenase |  | X | X | X |
| ugpC | -2.11 | ABC superfamily (atp_bind), sn-glycerol 3-phosphate transport protein |  |  |  |  |
| ycfH | -2.11 | putative metal-dependent hydrolase |  |  |  |  |
| yegH | -2.11 | putative inner membrane protein |  |  |  |  |
| ygdP | -2.11 | putative invasion protein; NTP pyrophosphohydrolase |  |  |  | X |
| deoC | -2.13 | 2-deoxyribose-5-phosphate aldolase |  |  |  |  |
| STM0081 | -2.13 | putative secreted protein |  |  |  |  |
| pfkB | -2.14 | 6-phosphofructokinase II |  |  |  |  |
| soxR | -2.14 | redox-sensing transcriptional activator SoxR, contains iron-sulfur center for redox-sensing (MerR family) |  |  |  |  |
| ugpA | -2.14 | ABC superfamily (membrane), sn-glycerol 3-phosphate transport protein |  |  |  |  |
| STM1123 | -2.15 | putative periplasmic protein |  |  |  |  |
| STM1809 | -2.15 | putative cytoplasmic protein |  |  |  |  |
| rhaS | -2.16 | positive regulator for rhaBAD operon (AraC/XylS family) |  |  | X |  |
| dmsA | -2.17 | anaerobic dimethyl sulfoxide reductase, subunit A |  |  |  | X |
| mscL | -2.17 | mechanosensitive channel |  |  |  | X |
| pgpB | -2.18 | phosphatidylglycerophosphate phosphatase B |  |  |  | X |
| ssaV | -2.19 | Secretion system apparatus: homology with the LcrD family of proteins |  | X |  |  |
| fidL | -2.2 | putative inner membrane protein |  | X |  | X |
| ybeL | -2.2 | putative cytoplasmic protein |  |  |  | X |
| yohI | -2.2 | putative nitrogen regulation protein |  |  |  |  |
| ssaT | -2.21 | Secretion system apparatus: homology with YscT of the secretion system of Yersinia |  | X | X |  |
| STM2208 | -2.21 | putative inner membrane protein |  |  |  |  |
| yohD | -2.21 | putative DedA family, membrane protein |  |  |  |  |
| modB | -2.22 | ABC superfamily (membrane), molybdate transporter |  |  |  |  |
| STM1562 | -2.22 | putative periplasmic transport protein |  | X |  |  |
| yhjQ | -2.22 | putative ATPase involved in chromosome partitioning |  |  |  |  |
| erfK | -2.23 | putative periplasmic protein |  |  |  |  |
| modC | -2.23 | ABC superfamily (atp_bind), molybdate transporter |  |  |  |  |
| STM1672 | -2.23 | putative cytoplasmic protein |  | X |  |  |
| pfkA | -2.24 | 6-phosphofructokinase I |  |  |  | X |
| ssaS | -2.24 | Secretion system apparatus: homology with YscS of the secretion system of Yersinia |  | X | X |  |
| ycgR | -2.24 | putative inner membrane protein |  |  |  |  |
| bcfA | -2.25 | fimbrial subunit |  |  |  |  |
| STM2126 | -2.25 | putative HlyD family secretion protein |  |  |  |  |
| caiF | -2.27 | transcriptional regulator of cai and fix operon |  |  |  | X |
| STM1858 | -2.27 | putative cytoplasmic protein |  | X |  |  |
| hopD | -2.28 | leader peptidase HopD |  |  |  |  |
| STM1633 | -2.28 | putative periplasmic binding protein |  | X |  |  |
| STM1988 | -2.28 | putative cytoplasmic protein |  |  |  |  |
| STM4310 | -2.28 | putative inner membrane protein |  | X | X |  |
| glgX | -2.29 | glycosyl hydrolase |  |  |  | X |
| STM1624 | -2.29 | putative cytoplasmic protein |  |  |  |  |
| ynhA | -2.29 | putative SufE protein probably involved in Fe-S center assembly |  |  |  |  |
| glgB | -2.3 | 1,4-alpha-glucan branching enzyme |  |  | X | X |
| STM1054 | -2.31 | Gifsy-2 prophage |  |  |  |  |
| ygiW | -2.31 | putative outer membrane protein |  |  |  |  |
| marT | -2.32 | putative transcriptional regulatory protein |  |  |  |  |
| pgtE | -2.32 | Phosphoglycerate transport: outer membrane protein E |  |  |  |  |
| yehV | -2.32 | putative transcriptional repressor (MerR family) |  |  |  |  |
| ymgE | -2.32 | putative transglycosylase-associated protein |  |  |  |  |
| flhB | -2.33 | putative part of export apparatus for flagellar proteins |  |  |  |  |
| yadI | -2.33 | putative PTS enzyme |  |  |  |  |
| astE | -2.35 | succinylglutamate desuccinylase |  |  |  |  |
| STM0810 | -2.35 | putative inner membrane protein |  |  |  |  |
| flhC | -2.36 | regulator of flagellar biosynthesis, acts on class 2 operons |  |  | X | X |
| yhjL | -2.36 | putative TPR-repeat-containing protein |  |  |  | X |
| ymdC | -2.36 | putative phospholipase |  |  |  | X |
| STM0381 | -2.37 | putative inner membrane protein |  |  |  |  |
| STM4206 | -2.37 | putative phage glucose translocase |  |  |  |  |
| glgC | -2.38 | glucose-1-phosphate adenylyltransferase |  |  |  | X |
| phnB | -2.38 | putative cytoplasmic protein |  |  |  |  |
| ycdC | -2.38 | putative transcriptional repressor (TetR/AcrR family) |  |  |  |  |
| acrR | -2.39 | acrAB operon repressor (TetR/AcrR family) |  |  | X |  |
| STM0053 | -2.39 | putative transcription regulator, histidine kinase for citrate |  |  | X |  |
| yaiA | -2.39 | putative cytoplasmic protein |  |  |  |  |
| pflA | -2.4 | pyruvate formate lyase activating enzyme 1 |  |  |  |  |
| srfA | -2.4 | ssrAB activated gene |  |  |  |  |
| STM0033 | -2.4 | putative 5'-nucleotidase |  | X |  |  |
| STM4257 | -2.4 | putative inner membrane or exported |  | X | X | X |
| gppA | -2.41 | guanosine pentaphosphatase and exopolyphosphatase |  |  |  |  |
| spaS | -2.41 | surface presentation of antigens; secretory proteins |  | X |  | X |
| STM2245 | -2.42 | putative outer membrane protein |  |  |  |  |
| tdcC | -2.43 | HAAAP family, L-threonine/ L-serine permease, anaerobically inducible |  |  |  |  |
| STM3681 | -2.44 | putative transcriptional regulator |  |  |  |  |
| STM4071 | -2.44 | putative Mannose-6-phosphate isomerase |  |  |  |  |
| fliR | -2.45 | putative flagellar biosynthetic protein |  |  |  |  |
| pykF | -2.45 | pyruvate kinase I (formerly F), fructose stimulated |  |  |  |  |
| ssaQ | -2.46 | Secretion system apparatus |  | X | X |  |
| STM2803 | -2.46 | putative regulatory protein, gntR family |  |  |  |  |
| PSLT040 | -2.47 |  |  |  |  |  |
| yeeY | -2.47 | putative transcriptional regulator, LysR family |  |  |  |  |
| ssaP | -2.5 | Secretion system apparatus |  | X |  |  |
| STM1055 | -2.51 | Gifsy-2 prophage |  |  |  |  |
| STM1147 | -2.51 | putative ACR related to the C-terminal domain of histone macroH2A1 |  |  |  | X |
| STM2585A | -2.51 | Gifsy-1 prophage: Homolog of pagK |  |  |  |  |
| sseF | -2.52 | Secretion system effector |  |  |  |  |
| ychM | -2.52 | putative SulP family transport protein |  |  |  |  |
| fliI | -2.53 | flagellum-specific ATP synthase |  |  |  | X |
| STM2137 | -2.54 | putative cytoplasmic protein |  |  |  |  |
| yhjO | -2.54 | glycosyltransferase, probably involved in cell wall biogenesis |  |  |  |  |
| ydeV | -2.56 | putative sugar kinase |  |  |  |  |
| STM0082 | -2.57 | putative secreted protein |  |  | X |  |
| STM1491 | -2.57 | ABC-type proline/glycine betaine transport systems, ATPase component |  |  |  |  |
| STM2904 | -2.57 | putative ABC-type transport system |  | X |  |  |
| glgA | -2.58 | glycogen synthase |  |  |  |  |
| otsB | -2.59 | trehalose-6-phosphate phophatase, biosynthetic |  |  |  |  |
| uspB | -2.61 | universal stress protein B, involved in stationary-phase resistance to ethanol |  |  |  | X |
| fliB | -2.62 | N-methylation of lysine residues in flagellin |  |  |  |  |
| fliF | -2.63 | flagellar biosynthesis; basal-body MS(membrane and supramembrane)-ring and collar protein |  |  |  |  |
| sscB | -2.63 | Secretion system chaparone |  | X |  |  |
| ydcX | -2.63 | putative inner membrane protein |  |  |  |  |
| ydiU | -2.63 | putative cytoplasmic protein |  |  |  | X |
| sanA | -2.64 | vancomycin sensitivity |  |  | X | X |
| sinR | -2.64 | transcriptional regulator |  |  | X |  |
| yeeZ | -2.64 | putative dehydratase |  |  |  |  |
| flgB | -2.65 | flagellar biosynthesis, cell-proximal portion of basal-body rod |  |  |  |  |
| yneB | -2.65 | putative fructose-1,6-bisphosphate aldolase |  |  |  | X |
| yjcC | -2.67 | putative diguanylate cyclase/phosphodiesterase |  |  |  |  |
| STM4575 | -2.68 | putative outer membrane protein |  |  |  |  |
| adiY | -2.7 | transcriptional activator of adiA (AraC/XylS family) |  |  | X |  |
| mtlR | -2.7 | repressor for mtl |  |  |  |  |
| STM0860 | -2.7 | putative inner membrane protein |  | X |  |  |
| mug | -2.72 | DNA glycosylase, G/U mismatch specific |  |  |  |  |
| suhB | -2.72 | inositol monophosphatase |  |  |  |  |
| yjfO | -2.72 | putative lipoprotein |  |  |  | X |
| ftnB | -2.73 | ferritin-like protein |  |  |  | X |
| STM1484 | -2.73 | putative protease |  |  |  |  |
| STM2475 | -2.73 | putative cytoplasmic protein |  |  |  |  |
| yqjG | -2.73 | putative glutathione S-transferase |  |  |  | X |
| malT | -2.74 | transcriptional activator of the mal genes, binds inducer (maltotriose) and ATP (LysR familiy) |  |  |  | X |
| orf319 | -2.75 | putative inner membrane protein |  |  |  | X |
| STM3152 | -2.75 | putative methyl-accepting chemotaxis protein |  |  |  |  |
| rnc | -2.76 | RNase III, ds RNA |  |  |  |  |
| ssaD | -2.78 | Secretion system apparatus |  | X |  |  |
| yhbP | -2.78 | putative cytoplasmic protein |  |  |  |  |
| zur | -2.78 | transcriptional repressor of znuABC operon (Fur family) |  |  |  | X |
| STM4219 | -2.79 | putative cytoplasmic protein |  |  |  |  |
| yneA | -2.79 | putative ABC superfamily (peri_perm), sugar transport protein |  |  |  |  |
| pagK | -2.8 | PhoPQ-activated gene |  | X |  | X |
| ybeQ | -2.8 | putative TPR repeat protein |  |  |  |  |
| yhhT | -2.8 | putative PerM family permease |  |  |  |  |
| aidB | -2.81 | putative acyl-CoA dehydrogenase; adaptive response (transcription activated by Ada) |  |  |  |  |
| exbD | -2.81 | uptake of enterochelin; tonB-dependent uptake of B colicins |  |  |  |  |
| cheA | -2.82 | sensory histitine protein kinase, transduces signal between chemo- signal receptors and CheB and CheY |  |  |  | X |
| pagC | -2.82 | PhoP regulated: reduced macrophage survival | X | X |  | X |
| proP | -2.83 | MFS family, low-affinity proline transporter (proline permease II) |  |  | X | X |
| yajO | -2.83 | putative oxidoreductase / K + channel protein |  |  |  |  |
| soxS | -2.84 | transcriptional activator of superoxide response regulon (AraC/XylS family) |  |  |  |  |
| ydcW | -2.84 | putative aldehyde dehydrogenase |  |  |  |  |
| bcsC | -2.85 | endo-1,4-D-glucanase |  |  |  |  |
| flgA | -2.86 | flagellar biosynthesis; assembly of basal-body periplasmic P ring |  |  |  |  |
| PSLT039 | -2.86 |  |  |  |  |  |
| dbpA | -2.87 | ATP-dependent RNA helicase, stimulated by 23S rRNA |  |  |  |  |
| fliD | -2.87 | flagellar biosynthesis; filament capping protein; enables filament assembly |  |  |  | X |
| sifB | -2.87 | Salmonella translocated effector: translocated by SPI-2 |  | X |  | X |
| gabT | -2.88 | 4-aminobutyrate aminotransferase |  |  |  |  |
| yehX | -2.88 | putative ABC-type proline/glycine betaine transport system, ATPase component |  |  |  |  |
| modA | -2.89 | ABC superfamily (peri_perm), molybdate transporter |  |  |  | X |
| STM1987 | -2.89 | putative inner membrane protein |  |  |  |  |
| ybaJ | -2.89 | putative cytoplasmic protein |  | X |  | X |
| yhjR | -2.89 | putative cytoplasmic protein |  |  |  |  |
| cheB | -2.9 | methyl esterase, response regulator for chemotaxis (cheA sensor) |  |  |  | X |
| srfC | -2.9 | ssrAB activated gene: predicted coiled-coil structure |  |  |  |  |
| ssaO | -2.9 | Secretion system apparatus |  | X |  |  |
| fruA | -2.91 | Sugar Specific PTS system, fructose-specific transport protein |  |  |  |  |
| phsA | -2.91 | Hydrogen sulfide production: membrane anchoring protein |  |  |  | X |
| yhjN | -2.91 | putative cellulose synthase |  |  |  |  |
| STM1330 | -2.92 | putative DNA/RNA non-specific endonuclease |  |  |  |  |
| astB | -2.93 | succinylarginine dihydrolase |  |  |  |  |
| spaQ | -2.93 | surface presentation of antigens; secretory proteins |  | X |  |  |
| galP | -2.96 | MFS family, galactose:proton symporter |  |  |  |  |
| STM0948 | -2.96 | putative cytoplasmic protein |  |  |  |  |
| cfa | -3 | cyclopropane fatty acyl phospholipid synthase |  |  |  |  |
| cigR | -3.02 | putative inner membrane protein |  |  |  |  |
| slrP | -3.02 | leucine-rich repeat protein |  |  | X | X |
| STM1698 | -3.02 | putative inner membrane protein |  |  |  |  |
| STM3155 | -3.02 | putative cytoplasmic protein |  |  | X |  |
| yjcB | -3.02 | putative inner membrane protein |  |  | X |  |
| fliP | -3.03 | flagellar biosynthesis |  |  |  | X |
| ycgB | -3.03 | putative cytoplasmic protein |  |  |  | X |
| yhjS | -3.03 | putative cytoplasmic protein |  |  |  |  |
| yjgB | -3.03 | putative alcohol dehydrogenase |  |  |  |  |
| STM1934 | -3.04 | putative outer membrane lipoprotein |  |  |  |  |
| flgC | -3.05 | flagellar biosynthesis, cell-proximal portion of basal-body rod |  |  |  |  |
| sufS | -3.05 | selenocysteine lyase |  |  |  |  |
| ssaC | -3.06 | Secretion system apparatus |  | X |  |  |
| STM2715 | -3.07 | Fels-2 prophage: probable prophage lysozyme |  | X |  |  |
| ychH | -3.08 | putative inner membrane protein |  |  |  | X |
| flgI | -3.09 | putative flagella basal body protein |  |  |  |  |
| yhjT | -3.09 | putative inner membrane protein |  |  |  |  |
| phsB | -3.12 | Hydrogen sulfide production: iron- sulfur subunit; electron transfer |  |  |  |  |
| ssaN | -3.12 | Secretion system apparatus: homology with the YscN family of proteins |  |  |  | X |
| fic | -3.14 | putative cell filamentation protein, stationary phase induced gene, affects cell division |  |  |  |  |
| ssaB | -3.14 | Secretion system apparatus |  | X |  |  |
| STM1056 | -3.15 | Gifsy-2 prophage; Homolog of msgA |  |  |  |  |
| yohC | -3.16 | paral putative transport protein |  |  |  |  |
| manY | -3.19 | Sugar Specific PTS family, mannose-specific enzyme IIC |  |  |  | X |
| STM1026 | -3.19 | Gifsy-2 prophage |  |  |  |  |
| flhD | -3.2 | regulator of flagellar biosynthesis, acts on class 2 operons |  |  | X | X |
| ybhK | -3.21 | putative cytoplasmic protein |  |  |  |  |
| yjfN | -3.22 | putative inner membrane protein |  |  |  | X |
| hilA | -3.24 | invasion genes transcription activator |  | X | X | X |
| ompA | -3.27 | putative hydrogenase, membrane component |  |  |  | X |
| STM4258 | -3.28 | putative methyl-accepting chemotaxis protein |  | X | X | X |
| manX | -3.31 | Sugar Specific PTS family, mannose-specific enzyme IIAB |  |  |  | X |
| STM4316 | -3.31 | putative cytoplasmic protein |  | X | X |  |
| grxB | -3.32 | glutaredoxin 2 |  |  |  | X |
| flhE | -3.33 | flagellar protein |  |  |  |  |
| blc | -3.34 | outer membrane lipoprotein (lipocalin) |  |  |  |  |
| pipB2 | -3.34 | Pathogenicity island encoded protein: SPI3 |  | X |  | X |
| yqhE | -3.37 | 2,5-diketo-D-gluconate reductase A |  |  |  |  |
| ratA | -3.41 | putative outer membrane protein |  |  |  |  |
| trg | -3.42 | methyl-accepting chemotaxis protein III, ribose and galactose sensor receptor |  |  |  | X |
| ydeJ | -3.42 | putative Competence-damaged protein |  |  |  |  |
| fliH | -3.43 | flagellar biosynthesis; possible export of flagellar proteins |  |  |  |  |
| sseJ | -3.44 | Salmonella translocated effector: regulated by SPI-2 |  | X |  |  |
| yhcN | -3.44 | putative outer membrane protein |  |  |  |  |
| ycfQ | -3.45 | putative transcriptional repressor (TetR/AcrR family) |  |  |  |  |
| yebW | -3.46 | putative inner membrane lipoprotein |  |  |  |  |
| sseG | -3.47 | Secretion system effector |  | X |  |  |
| ugpQ | -3.47 | glycerophosphodiester phosphodiesterase, cytosolic |  |  |  |  |
| csgC | -3.49 | putative curli production protein |  | X |  |  |
| narY | -3.49 | nitrate reductase 2, beta subunit |  |  |  |  |
| yobG | -3.5 | putative inner membrane protein |  |  |  | X |
| ssrA | -3.52 | Secretion system regulator:Sensor component |  | X |  |  |
| flgK | -3.53 | flagellar biosynthesis, hook-filament junction protein 1 |  |  |  | X |
| otsA | -3.54 | trehalose-6-phosphate synthase |  |  |  |  |
| STM1561 | -3.54 | putative outer membrane or secreted lipoprotein |  |  |  |  |
| celG | -3.55 | putative glucosidase |  |  | X | X |
| sscA | -3.55 | Secretion system chaparone |  |  |  | X |
| ybiI | -3.55 | putative DnaK suppressor protein |  |  |  |  |
| hilC | -3.56 | bacterial regulatory helix-turn-helix proteins, araC family |  | X | X | X |
| STM1851 | -3.57 | putative cytoplasmic protein |  |  |  |  |
| talA | -3.58 | transaldolase A |  |  |  |  |
| acnA | -3.61 | aconitate hydratase 1 |  |  |  | X |
| STM1398 | -3.61 |  |  | X |  |  |
| sptP | -3.62 | protein tyrosine phosphate |  | X | X | X |
| ssaR | -3.62 | Secretion system apparatus: homology with YscR of the secretion system of Yersinia |  | X |  |  |
| ugpB | -3.62 | ABC superfamily (peri_perm), sn-glycerol 3-phosphate transport protein |  |  |  |  |
| aer | -3.63 | aerotaxis sensor receptor, senses cellular redox state or proton motive force |  |  |  |  |
| STM1967 | -3.63 | putative 50S ribosomal protein |  |  |  |  |
| fljB | -3.64 | Flagellar synthesis: phase 2 flagellin (filament structural protein) |  | X |  |  |
| yedP | -3.65 | putative hydrolase of the HAD superfamily |  |  |  |  |
| STM4574 | -3.67 | putative outer membrane protein |  |  |  |  |
| ybhO | -3.67 | cardiolipin (CL) synthase |  |  |  |  |
| STM2405 | -3.69 | putative thiamine pyrophosphate enzymes |  |  |  |  |
| yibF | -3.69 | putative glutathione S-transferase |  |  |  |  |
| STM1261 | -3.7 | putative cytoplasmic protein |  |  |  |  |
| tsr | -3.71 | methyl-accepting chemotaxis protein I, serine sensor receptor |  |  |  | X |
| osmC | -3.74 | putative resistance protein, osmotically inducible |  |  |  |  |
| STM0972 | -3.74 | homologous to secreted protein sopD |  |  |  |  |
| yhfG | -3.74 | putative cytoplasmic protein |  |  |  |  |
| sufC | -3.75 | putative ABC superfamily (atp_bind) transport protein |  |  |  |  |
| ydiY | -3.76 | putative salt-induced outer membrane protein |  |  |  |  |
| narW | -3.8 | nitrate reductase 2, delta subunit, assembly function |  |  |  |  |
| fliL | -3.85 | flagellar biosynthesis |  |  |  |  |
| spaR | -3.85 | surface presentation of antigens; secretory proteins |  | X |  | X |
| STM3154 | -3.86 | putative ATP-dependent RNA helicase-like protein |  |  |  | X |
| STM1329 | -3.89 | putative inner membrane protein |  |  |  |  |
| sufD | -3.89 | required for stability of iron-sulfur component of FhuF |  |  |  |  |
| manZ | -3.91 | Sugar Specific PTS family, mannose-specific enzyme IID |  |  |  | X |
| sugE | -3.93 | putative DMT superfamily transport protein |  |  |  |  |
| rpsV | -3.95 | 30S ribosomal subunit protein S22 |  |  |  |  |
| yebF | -3.95 | putative periplasmic protein |  |  |  | X |
| STM1239 | -3.96 | putative cytoplasmic protein |  | X | X | X |
| fliO | -3.98 | flagellar biosynthesis |  | X |  |  |
| yncB | -3.98 | putative NADP-dependent oxidoreductase |  |  |  | X |
| yceK | -4.02 | putative outer membrane lipoprotein |  |  |  |  |
| yqjK | -4.04 | putative inner membrane protein |  |  |  |  |
| STM3774 | -4.05 | putative inner membrane protein |  |  |  |  |
| yehY | -4.11 | putative ABC-type proline/glycine betaine transport systems, permease component |  |  |  | X |
| yhjE | -4.12 | putative MFS family transport protein |  |  |  |  |
| sitD | -4.15 | Salmonella iron transporter: fur regulated |  |  |  |  |
| yahO | -4.16 | putative periplasmic protein |  |  |  | X |
| aldB | -4.25 | aldehyde dehydrogenase B (lactaldehyde dehydrogenase) |  |  |  | X |
| STM4259 | -4.25 | putative ABC exporter outer membrane component homolog |  | X |  | X |
| yhjH | -4.27 | putative Diguanylate cyclase/phosphodiesterase domain 3 |  |  |  | X |
| sicP | -4.29 | chaparone, related to virulence |  | X | X | X |
| chaB | -4.31 | cation transport regulator |  |  |  |  |
| sseD | -4.35 | Secretion system effector |  | X |  |  |
| yfdC | -4.35 | putative transport |  |  |  |  |
| sufA | -4.41 | putative HesB-like domain |  |  |  |  |
| ggt | -4.49 | gamma-glutamyltranspeptidase |  |  |  |  |
| ssaK | -4.49 | Secretion system apparatus |  | X |  |  |
| fliC | -4.5 | flagellar biosynthesis; flagellin, filament structural protein | X |  |  | X |
| sseE | -4.5 | Secretion system effector |  | X |  |  |
| phsC | -4.52 | Hydrogen sulfide production: membrane anchoring protein |  |  |  |  |
| STM1267 | -4.62 | putative cytoplasmic protein |  | X |  |  |
| STM3156 | -4.67 | putative cytoplasmic protein |  |  |  |  |
| yhhA | -4.69 | putative outer membrane protein |  |  |  |  |
| STM4262 | -4.7 | putative ABC-type bacteriocin/lantibiotic exporter, contain an N-terminal double-glycine peptidase domain |  | X |  | X |
| yeaH | -4.72 | putative cytoplasmic protein |  |  |  | X |
| tktB | -4.75 | transketolase 2, isozyme |  |  |  |  |
| sufB | -4.79 | putative ABC transporter |  |  |  |  |
| flgF | -4.81 | flagellar biosynthesis, cell-proximal portion of basal-body rod |  |  |  | X |
| flgG | -4.82 | flagellar biosynthesis, cell-distal portion of basal-body rod |  |  |  |  |
| cheZ | -4.84 | chemotactic response; CheY protein phophatase |  |  |  | X |
| STM3362 | -4.92 | putative periplasmic protein |  |  | X | X |
| STM1731 | -5.03 | putative catalase |  |  |  | X |
| STM3688 | -5.05 | putative cytoplasmic protein |  |  |  |  |
| flgD | -5.08 | flagellar biosynthesis, initiation of hook assembly |  |  |  | X |
| yohF | -5.09 | putative oxidoreductase |  |  |  |  |
| sopB | -5.11 | Salmonella outer protein: homologous to ipgD of Shigella |  | X |  | X |
| rtsA | -5.16 | putative AraC-type DNA-binding domain-containing protein |  | X | X |  |
| iagB | -5.2 | cell invasion protein |  | X |  | X |
| ssrB | -5.24 | Secretion system regulator: transcriptonal activator, homologous with degU/uvrY/bvgA |  | X |  | X |
| STM0080 | -5.25 | putative outer membrane lipoprotein |  |  |  |  |
| STM1397 | -5.26 |  |  | X |  |  |
| bfr | -5.29 | bacterioferrin, an iron storage homoprotein |  | X |  |  |
| STM4312 | -5.31 | putative phage protein |  | X | X |  |
| ugtL | -5.34 | putative membrane protein: homology with chitinase from Schizosaccharomyces |  |  |  | X |
| yegS | -5.36 | putative diacylglycerol kinase catalytic domain |  |  |  |  |
| STM1558 | -5.37 | putative glycosyl hydrolase |  |  |  | X |
| STM4313 | -5.42 | putative cytoplasmic protein |  | X | X |  |
| invA | -5.43 | invasion protein |  | X | X | X |
| adhE | -5.44 | iron-dependent alcohol dehydrogenase of the multifunctional alcohol dehydrogenase AdhE |  |  |  | X |
| yfbK | -5.46 | putative von Willebrand factor, vWF type A domain |  |  |  |  |
| STM4260 | -5.47 | membrane permease, predicted cation efflux pump |  | X | X | X |
| STM1301 | -5.5 | putative mutator MutT protein |  |  |  |  |
| fliN | -5.52 | flagellar biosynthesis, component of motor switch and energizing |  |  |  |  |
| flgE | -5.55 | flagellar biosynthesis, hook protein |  |  |  | X |
| fliK | -5.59 | flagellar hook-length control protein |  |  |  |  |
| rtsB | -5.59 | putative bacterial regulatory proteins, luxR family |  | X |  |  |
| sseC | -5.66 | Secretion system effector |  | X |  | X |
| sdiA | -5.69 | transcriptional regulator of ftsQAZ gene cluster (LuxR/UhpA family) |  |  |  | X |
| STM3132 | -5.72 | putative xylanase/chitin deacetylase |  |  |  | X |
| treA | -5.72 | trehalase, periplasmic |  |  |  | X |
| fliJ | -5.73 | flagellar fliJ protein |  |  |  |  |
| fliM | -5.81 | flagellar biosynthesis, component of motor switch and energizing |  |  |  | X |
| spy | -5.85 | periplasmic protein related to spheroblast formation |  |  |  |  |
| ssaL | -5.89 | Secretion system apparatus |  | X |  |  |
| yodD | -5.9 | putative cytoplasmic protein |  |  |  |  |
| motB | -5.94 | enables flagellar motor rotation, linking torque machinery to cell wall |  |  |  | X |
| cheY | -5.96 | chemotaxis regulator, transmits chemoreceptor signals to flagelllar motor components |  |  |  | X |
| prgH | -6.04 | cell invasion protein |  | X |  | X |
| STM2780 | -6.04 | Homolog of pipB, putative pentapeptide repeats (8 copies) |  | X |  |  |
| yehZ | -6.05 | putative ABC superfamily (bind_prot) transport protein (possibly glycine betaine choline transport for osmoprotection) |  |  |  | X |
| fliT | -6.06 | flagellar biosynthesis; possible export chaperone for FliD |  |  |  |  |
| STM0362 | -6.13 | putative cytoplasmic protein |  |  |  |  |
| psiF | -6.14 | induced by phosphate starvation |  |  |  |  |
| STM2404 | -6.14 | putative chloride channel permease |  |  |  |  |
| sprB | -6.2 | transcriptional regulator |  | X |  | X |
| ygaE | -6.2 | putative transcriptional repressor (GntR familiy) |  |  |  |  |
| fliZ | -6.22 | putative regulator of FliA |  |  |  | X |
| avrA | -6.28 | putative inner membrane protein |  | X | X | X |
| sipB | -6.29 | cell invasion protein |  |  |  | X |
| cheR | -6.31 | glutamate methyltransferase, response regulator for chemotaxis |  |  |  | X |
| motA | -6.34 | proton conductor component of motor, torque generator |  |  |  | X |
| invH | -6.35 | invasion protein |  | X | X |  |
| ssaI | -6.36 | Secretion system apparatus |  | X |  |  |
| STM1328 | -6.4 | putative outer membrane protein | X |  | X | X |
| poxB | -6.42 | pyruvate dehydrogenase/oxidase FAD and thiamine PPi cofactors, cytoplasmic in absence of cofactors |  |  |  |  |
| invC | -6.51 | surface presentation of antigens; secretory proteins |  |  | X | X |
| STM3133 | -6.52 | putative amidohydrolase |  |  |  | X |
| yhbO | -6.54 | putative intracellular proteinase |  |  |  |  |
| invB | -6.57 | surface presentation of antigens; secretory proteins |  | X |  | X |
| osmE | -6.58 | transcriptional activator of ntrL gene |  |  |  | X |
| yccJ | -6.6 | putative cytoplasmic protein |  |  |  |  |
| spaP | -6.62 | surface presentation of antigens; secretory proteins |  | X | X | X |
| ssaG | -6.73 | Secretion system apparatus |  | X |  |  |
| fliS | -6.83 | flagellar biosynthesis; repressor of class 3a and 3b operons (RflA activity) |  |  |  | X |
| ssaH | -6.86 | Secretion system apparatus |  | X |  |  |
| STM1089 | -6.91 | putative inner membrane protein |  | X |  |  |
| orgA | -6.92 | putative flagellar biosynthesis/type III secretory pathway protein |  | X | X | X |
| hilD | -6.93 | regulatory helix-turn-helix proteins, araC family |  | X | X | X |
| ecnR | -7.26 | putative bacterial regulatory protein, luxR family |  |  |  |  |
| cheW | -7.27 | purine-binding chemotaxis protein; regulation |  |  |  | X |
| msyB | -7.28 | acidic protein suppresses mutants lacking function of protein export |  |  |  |  |
| ssaJ | -7.28 | Secretion system apparatus: homology with the yscJ/mxiJ/prgK family of lipoproteins |  | X |  | X |
| yeaQ | -7.43 | putative inner membrane protein |  | X |  | X |
| invG | -7.56 | invasion protein; outer membrane |  | X | X | X |
| sipC | -7.6 | cell invasion protein | X | X |  | X |
| yqjE | -7.62 | putative inner membrane protein |  |  |  |  |
| invF | -7.69 | invasion protein |  | X | X | X |
| STM2870 | -7.75 | putative inner membrane protein |  | X | X |  |
| invI | -7.76 | surface presentation of antigens; secretory proteins |  | X |  |  |
| tcp | -7.91 | methyl-accepting transmembrane citrate/phenol chemoreceptor |  |  |  | X |
| sopA | -7.93 | Secreted effector protein of Salmonella dublin |  |  |  | X |
| iacP | -7.94 | putative acyl carrier protein |  | X | X | X |
| flgL | -8.06 | flagellar biosynthesis; hook-filament junction protein |  |  | X | X |
| prgK | -8.27 | cell invasion protein; lipoprotein, may link inner and outer membranes |  | X |  | X |
| sodC | -8.3 | copper/zinc superoxide dismutase |  |  |  |  |
| flgN | -8.33 | flagellar biosynthesis: belived to be export chaperone for FlgK and FlgL |  |  |  | X |
| prgJ | -8.36 | cell invasion protein; cytoplasmic |  | X |  | X |
| sopE2 | -8.64 | TypeIII-secreted protein effector: invasion-associated protein |  | X | X | X |
| prgI | -8.65 | cell invasion protein; cytoplasmic |  | X |  |  |
| adhP | -8.66 | alcohol dehydrogenase, propanol preferring |  |  |  |  |
| STM4261 | -8.67 | putative inner membrane protein |  | X | X | X |
| STM4519 | -8.71 | putative NAD-dependent aldehyde dehydrogenase |  |  |  |  |
| wraB | -8.77 | trp-repressor binding protein |  |  |  | X |
| STM1300 | -8.79 | putative periplasmic protein |  |  |  | X |
| STM2585 | -8.91 | Gifsy-1 prophage: similar to transpose |  |  |  |  |
| STM2139 | -9.13 | putative inner membrane protein |  | X |  |  |
| osmB | -9.36 | osmotically inducible lipoprotein |  |  |  |  |
| fliA | -9.49 | sigma F (sigma 28) factor of RNA polymerase, transcription of late flagellar genes (class 3a and 3b operons) |  |  |  | X |
| STM1560 | -9.54 | putative alpha amylase |  |  |  |  |
| invE | -9.9 | invasion protein |  | X |  | X |
| flgM | -10.09 | anti-FliA (anti-sigma) factor; also known as RflB protein |  |  |  | X |
| invJ | -10.12 | surface presentation of antigens; secretory proteins |  | X |  | X |
| yiaG | -10.17 | putative transcriptional regulator |  |  | X |  |
| yqjC | -10.17 | putative periplasmic protein |  |  |  | X |
| STM0359 | -10.31 | putative cytoplasmic protein |  |  |  |  |
| ybaY | -10.35 | glycoprotein/polysaccharide metabolism |  |  |  | X |
| STM2868 | -10.64 | putative cytoplasmic protein |  | X | X |  |
| sopD | -10.73 | secreted protein in the Sop family; transferred to eukaryotic cells |  |  |  | X |
| cheM | -10.78 | methyl accepting chemotaxis protein II, aspartate sensor-receptor |  |  |  | X |
| dps | -10.99 | stress response DNA-binding protein; starvation induced resistance to H2O2 | X |  |  | X |
| STM1629 | -11.31 | putative dipicolinate reductase |  | X | X |  |
| phoH | -11.78 | PhoB-dependent, ATP-binding pho regulon component |  |  |  |  |
| yeaG | -11.81 | putative Ser protein kinase |  |  |  | X |
| ecnB | -12.19 | putative entericidin B precursor |  |  |  | X |
| ygaU | -12.72 | putative LysM domain |  |  |  |  |
| fbaB | -13.2 | 3-oxoacyl-[acyl-carrier-protein] synthase I |  |  |  |  |
| STM1841 | -13.44 | putative outer membrane or exported |  |  | X | X |
| ampH | -14.03 | penicillin- binding protein |  |  |  |  |
| sipA | -14.3 | cell invasion protein | X | X |  | X |
| elaB | -14.43 | putative inner membrane protein |  |  |  |  |
| sipD | -14.99 | cell invasion protein |  | X | X | X |
| ydeI | -15.37 | putative periplasmic protein |  |  |  |  |
| ygdI | -16.14 | putative lipoprotein |  |  |  | X |
| pipC | -16.34 | Pathogenicity island encoded protein: homologous to ipgE of Shigella |  | X |  | X |
| yciE | -16.68 | putative cytoplasmic protein |  |  |  |  |
| katE | -16.7 | catalase; hydroperoxidase HPII(III), RpoS dependent |  |  |  | X |
| ygaM | -17.53 | putative inner membrane protein |  |  |  |  |
| ybgS | -17.94 | putative homeobox protein |  |  |  |  |
| STM1513 | -18.8 | putative cytoplasmic protein |  |  |  |  |
| ymdF | -20.76 | putative cytoplasmic protein |  |  |  |  |
| hfq | -21.66 | host factor I for bacteriophage Q beta replication, a growth-related protein |  |  | X | X |
| yghA | -24.47 | putative oxidoreductase |  |  |  | X |
| yciF | -24.49 | putative cytoplasmic protein |  |  |  |  |
| yjbJ | -28.92 | putative cytoplasmic protein |  |  |  | X |
| yciG | -33.88 | putative cytoplasmic protein |  |  |  |  |
| osmY | -34.13 | hyperosmotically inducible periplasmic protein, RpoS-dependent stationary phase gene | X |  |  | X |

^a^Gene names according to ColiBase [3]

^b^ Product according to KEGG (http://www.genome.jp/kegg/; [4])

^c^ Assignment according to [5]

^d^ Assignment according to HGT-GB (http://www.tinet.org/~debb/HGT/; [6]
