# Supplementary material for: Deep Sequencing Analysis of Small Noncoding RNA and mRNA Targets of the Global Post-Transcriptional Regulator, Hfq
Source: PLoS Genet. 2008 Aug 22;4(8):e1000163. doi: 10.1371/journal.pgen.1000163 (PMC2515195; doi:10.1371/journal.pgen.1000163)
Supplement: Table S2 — Deregulated genes in Δhfq after 12 hrs SPI-inducing conditions. (0.21 MB DOC) [file pgen.1000163.s007.doc]

**Table S2: Deregulated genes in Δ*hfq* after 12 hrs SPI-inducing conditions**

| **Gene name^a^** | **Fold change** | **Product^b^** | **2D-analysis^c^** | **HGT^d^** |
| --- | --- | --- | --- | --- |
| ybfM | 62.11 | putative outer membrane protein | X |  |
| pagC | 45.66 | PhoP regulated: reduced macrophage survival | X | X |
| ybfN | 34.36 | putative lipoprotein |  |  |
| virK | 16.31 | virulence gene; homologous sequence to virK in Shigella |  |  |
| yggN | 8.70 | putative periplasmic protein |  |  |
| ugtL | 8.70 | putative membrane protein: homology with chitinase from Schizosaccharomyces |  | X |
| rseA | 8.20 | anti sigma E (sigma 24) factor, negative regulator |  |  |
| ddg | 7.04 | cold shock-induced palmitoleoyl transferase |  |  |
| ygiM | 6.85 | putative SH3 domain protein |  |  |
| STM1044 | 6.45 |  |  |  |
| STM1253 | 6.33 | putative inner membrane protein |  | X |
| STM1583 | 6.21 | putative cytoplasmic protein |  |  |
| rseB | 6.17 | anti sigma E (sigma 24) factor, negative regulator |  |  |
| STM2585A | 6.10 | Gifsy-1 prophage: Homolog of pagK |  |  |
| yhjW | 6.06 | putative membrane-associated, metal-dependent hydrolase |  |  |
| STM4257 | 5.68 | putative inner membrane or exported |  | X |
| STM4260 | 5.21 | membrane permease, predicted cation efflux pump |  | X |
| STM1698 | 5.18 | putative inner membrane protein |  |  |
| STM4259 | 5.18 | putative ABC exporter outer membrane component homolog |  | X |
| yraP | 4.85 | paral putative periplasmic protein | X |  |
| phoN | 4.78 | non-specific acid phosphatase |  |  |
| sscB | 4.69 | Secretion system chaperone |  | X |
| sixA | 4.65 | phosphohistidine phosphatase |  |  |
| pmrD | 4.55 | polymyxin resistance protein B |  |  |
| mig-14 | 4.46 | putative transcription activator |  |  |
| sseI | 4.46 | Gifsy-2 prophage; putative type III secreted protein |  |  |
| yobG | 4.44 | putative inner membrane protein |  |  |
| STM1330 | 4.44 | putative DNA/RNA non-specific endonuclease |  |  |
| sseJ | 4.39 | Salmonella translocated effector: regulated by SPI-2 |  | X |
| yraO | 4.29 | putative phosphoheptose isomerase |  |  |
| gcvH | 4.26 | glycine cleavage complex protein H, carrier of aminomethyl moiety via covalently bound lipoyl cofactor |  |  |
| rpoE | 4.18 | sigma E (sigma 24 ) factor of RNA polymerase, response to periplasmic stress |  |  |
| gcvP | 4.17 | glycine cleavage complex protein P, glycine decarboxylase |  |  |
| STM1854 | 3.94 | putative inner membrane protein |  |  |
| ompC | 3.91 | outer membrane protein 1b (ib;c), porin |  |  |
| htrA | 3.91 | periplasmic serine protease Do, heat shock protein | X |  |
| pagK | 3.91 | PhoPQ-activated gene |  | X |
| ybhQ | 3.88 | putative inner membrane protein |  |  |
| rna | 3.82 | RNase I, cleaves phosphodiester bond between any two nucleotides |  |  |
| hflK | 3.77 | with HflC, part of modulator for protease specific for FtsH phage lambda cII repressor |  |  |
| pgtE | 3.65 | Phosphoglycerate transport: outer membrane protein E |  |  |
| ydgR | 3.64 | putative POT family, peptide transport protein |  |  |
| cutC | 3.57 | copper homeostasis protein |  |  |
| rseC | 3.56 | regulator of sigma E (sigma 24) factor |  |  |
| STM2303 | 3.56 | putative inner membrane protein |  |  |
| pdgL | 3.38 | Periplasmic dipeptidase for D-ala-D-ala digestion in peptidoglycan |  |  |
| hflC | 3.36 | with HflK, part of modulator for protease specific for FtsH phage lambda cII repressor |  |  |
| sseD | 3.24 | Secretion system effector |  | X |
| pagD | 3.23 | PhoP regulated |  | X |
| STM4504 | 3.13 | putative cytoplasmic protein |  |  |
| yaeT | 3.13 | putative outer membrane antigen | X |  |
| hflX | 3.11 | putative GTP-ase, together with HflCK possibly involved in phage lambda cII repressor stability |  |  |
| yhjJ | 3.08 | putative Zn-dependent peptidase |  |  |
| phoP | 3.06 | response regulator in two-component regulatory system with PhoQ, transcribes genes expressed under low Mg+ concentration (OmpR family) |  |  |
| STM1052 | 3.06 |  |  |  |
| ycbK | 3.03 | putative outer membrane protein |  |  |
| STM1697 | 2.99 | putative Diguanylate cyclase/phosphodiesterase domain 2 |  |  |
| sspH2 | 2.96 | Leucine-rich repeat protein, induced by the SPI-2 regulator ssrA/B |  |  |
| hlpA | 2.91 | histone-like protein, located in outer membrane |  |  |
| gst | 2.89 | glutathionine S-transferase |  |  |
| STM1839 | 2.88 | putative periplasmic or exported protein |  |  |
| STM2447 | 2.83 | putative outer membrane lipoprotein |  |  |
| gcvT | 2.82 | glycine cleavage complex protein T, aminomethyltransferase, tetrahydrofolate-dependent |  |  |
| STM3036 | 2.72 | putative inner membrane protein |  |  |
| yfcN | 2.67 | putative Smr domain |  |  |
| cca | 2.62 | tRNA nucleotidyl transferase |  |  |
| surA | 2.62 | peptidyl-prolyl cis-trans isomerase, survival protein | X |  |
| mreB | 2.57 | rod shape-determining protein; HSP70 class molecular chaperones involved in cell morphogenesis |  |  |
| ycbL | 2.56 | putative Metallo-beta-lactamase |  |  |
| citA | 2.55 | citrate-proton symporter |  |  |
| STM0081 | 2.53 | putative secreted protein |  |  |
| ybhR | 2.50 | putative ABC superfamily (membrane) transport protein |  |  |
| lpxD | 2.49 | UDP-3-O-(3-hydroxymyristoyl)-glucosamine n-acyltransferase |  |  |
| STM2585 | 2.49 | Gifsy-1 prophage: similar to transpose |  |  |
| STM4261 | 2.46 | putative inner membrane protein |  | X |
| ydiV | 2.44 | putative Diguanylate cyclase/phosphodiesterase domain 1 |  |  |
| kdgK | 2.44 | ketodeoxygluconokinase |  |  |
| yraR | 2.43 | putative nucleoside-diphosphate-sugar epimerase |  |  |
| pdxA | 2.38 | NAD-dependent dehydrogenase/carboxylase; pyridoxine phosphate biosynthetic protein PdxJ-PdxA subunit |  |  |
| yfiD | 2.33 | putative formate acetyltransferase |  |  |
| STM0082 | 2.22 | putative secreted protein |  |  |
| yfeK | 2.21 | putative periplasmic protein |  |  |
| yidY | 2.19 | putative MFS family tranport protein (1st mdule) |  |  |
| STM1940 | 2.16 | putative cell wall-associated hydrolase |  |  |
| bacA | 2.15 | bacitracin resistance; possibly phosphorylates undecaprenol |  |  |
| yiiD | 2.14 | putative acetyltransferase |  |  |
| aphA | 2.13 | non-specific acid phosphatase/phosphotransferase, class B | X |  |
| yijD | 2.07 | putative inner membrane protein |  |  |
| ygcA | 2.05 | putative RNA methyltransferase |  |  |
| thrC | 2.03 | threonine synthase |  |  |
| yheO | 2.02 | putative regulator |  |  |
| yciE | -2.04 | putative cytoplasmic protein |  |  |
| yhjH | -2.05 | putative Diguanylate cyclase/phosphodiesterase domain 3 |  |  |
| wraB | -2.07 | trp-repressor binding protein |  |  |
| ygaM | -2.10 | putative inner membrane protein |  |  |
| nrdA | -2.13 | ribonucleoside diphosphate reductase 1, alpha subunit |  |  |
| STM3362 | -2.16 | putative periplasmic protein |  |  |
| glmS | -2.18 | L-glutamine:D-fructose-6-phosphate aminotransferase |  |  |
| mtlR | -2.23 | repressor for mtl |  |  |
| ybgS | -2.23 | putative homeobox protein |  |  |
| cheZ | -2.27 | chemotactic response; CheY protein phophatase |  |  |
| yjfN | -2.29 | putative inner membrane protein |  |  |
| ecnR | -2.29 | putative bacterial regulatory protein, luxR family |  |  |
| fbaB | -2.30 | 3-oxoacyl-[acyl-carrier-protein] synthase I |  |  |
| flgK | -2.33 | flagellar biosynthesis, hook-filament junction protein 1 |  |  |
| fimD | -2.35 | outer membrane usher protein |  |  |
| ydeZ | -2.38 | putative ABC superfamily (membrane), sugar transport protein |  |  |
| STM3156 | -2.39 | putative cytoplasmic protein |  |  |
| osmB | -2.43 | osmotically inducible lipoprotein |  |  |
| katE | -2.46 | catalase; hydroperoxidase HPII(III), RpoS dependent |  |  |
| potE | -2.46 | APC family, putrescine/ornithine antiporter |  |  |
| yciF | -2.50 | putative cytoplasmic protein |  |  |
| cheB | -2.52 | methyl esterase, response regulator for chemotaxis (cheA sensor) |  |  |
| STM0699 | -2.52 | putative cytoplasmic protein |  |  |
| gapA | -2.60 | glyceraldehyde-3-phosphate dehydrogenase A |  |  |
| STM3155 | -2.60 | putative cytoplasmic protein |  |  |
| cfa | -2.63 | cyclopropane fatty acyl phospholipid synthase |  |  |
| STM2281 | -2.64 | putative transcriptional regulator, LysR family |  |  |
| STM3154 | -2.65 | putative ATP-dependent RNA helicase-like protein |  |  |
| cyoC | -2.67 | cytochrome o ubiquinol oxidase subunit III |  |  |
| hha | -2.74 | hemolysin expression modulating protein (involved in environmental regulation of virulence factors) |  |  |
| cyoD | -2.74 | cytochrome o ubiquinol oxidase subunit IV |  |  |
| fliT | -2.82 | flagellar biosynthesis; possible export chaperone for FliD |  |  |
| fliZ | -2.82 | putative regulator of FliA |  |  |
| nuoA | -2.83 | NADH dehydrogenase I chain A |  |  |
| cheW | -2.88 | purine-binding chemotaxis protein; regulation |  |  |
| yccJ | -2.97 | putative cytoplasmic protein |  |  |
| ybaJ | -2.98 | putative cytoplasmic protein |  | X |
| agsA | -2.99 | Molecular chaperone (small heat shock protein) |  | X |
| flgL | -2.99 | Flagellar biosynthesis; hook-filament junction protein |  |  |
| STM1093 | -3.07 | putative cytoplasmic protein |  |  |
| fimC | -3.08 | periplasmic chaperone, required for type 1 fimbriae |  | X |
| ygaU | -3.16 | putative LysM domain |  |  |
| cyoB | -3.17 | cytochrome o ubiquinol oxidase subunit I |  |  |
| sodC | -3.19 | copper/zinc superoxide dismutase |  |  |
| osmE | -3.20 | transcriptional activator of ntrL gene |  |  |
| yqjC | -3.25 | putative periplasmic protein |  |  |
| STM0731 | -3.29 | putative inner membrane protein |  |  |
| tcp | -3.39 | methyl-accepting transmembrane citrate/phenol chemoreceptor |  | X |
| cheR | -3.44 | glutamate methyltransferase, response regulator for chemotaxis |  |  |
| flgN | -3.56 | flagellar biosynthesis: belived to be export chaperone for FlgK and FlgL |  |  |
| yeaG | -3.60 | putative Ser protein kinase |  |  |
| mopA | -3.65 | chaperone Hsp60 with peptide-dependent ATPase activity, affects cell division |  |  |
| cheY | -3.88 | chemotaxis regulator, transmits chemoreceptor signals to flagelllar motor components |  |  |
| fliS | -3.94 | flagellar biosynthesis; repressor of class 3a and 3b operons (RflA activity) |  |  |
| cyoA | -4.09 | cytochrome o ubiquinol oxidase subunit II | X |  |
| cheM | -4.29 | methyl accepting chemotaxis protein II, aspartate sensor-receptor |  |  |
| motB | -4.31 | enables flagellar motor rotation, linking torque machinery to cell wall |  |  |
| speF | -4.40 | ornithine decarboxylase isozyme, inducible |  |  |
| motA | -4.44 | proton conductor component of motor, torque generator |  |  |
| mopB | -4.63 | chaperone Hsp10, affects cell division |  |  |
| ygdI | -4.69 | putative lipoprotein |  |  |
| fimA | -4.76 | major type 1 subunit fimbrin (pilin) |  |  |
| ecnB | -6.04 | putative entericidin B precursor |  |  |
| orfX | -6.20 | putative cytoplasmic protein |  |  |
| osmY | -6.39 | hyperosmotically inducible periplasmic protein, RpoS-dependent stationary phase gene | X | X |
| dps | -6.62 | stress response DNA-binding protein; starvation induced resistance to H2O2 | X |  |
| STM1851 | -6.68 | putative cytoplasmic protein |  |  |
| yciG | -7.37 | putative cytoplasmic protein |  |  |
| flgM | -10.31 | anti-FliA (anti-sigma) factor; also known as RflB protein |  |  |
| STM1513 | -15.52 | putative cytoplasmic protein |  |  |
| fliC | -15.65 | flagellar biosynthesis; flagellin, filament structural protein | X |  |
| ymdF | -19.63 | putative cytoplasmic protein |  |  |
| hfq | -32.55 | host factor I for bacteriophage Q beta replication, a growth-related protein |  |  |
|  |  |  |  |  |

^a^Gene names according to ColiBase [3]

^b^ Product according to KEGG (http://www.genome.jp/kegg/; [4])

^c^ Assignment according to [5]

^d^ Assignment according to HGT-GB (http://www.tinet.org/~debb/HGT/; [6])
