# Supplementary material for: Deep Sequencing Analysis of Small Noncoding RNA and mRNA Targets of the Global Post-Transcriptional Regulator, Hfq
Source: PLoS Genet. 2008 Aug 22;4(8):e1000163. doi: 10.1371/journal.pgen.1000163 (PMC2515195; doi:10.1371/journal.pgen.1000163)
Supplement: Table S3 — Coverage of known and candidate Salmonella sRNA loci in pyrosequencing data. (0.26 MB DOC) [file pgen.1000163.s008.doc]

**Table S3: Coverage of known and candidate *Salmonella*** sRNA loci in pyrosequencing data

| **sRNA^a^** | **Alternative**  **IDs^b^** | **Identification^c^** | **Adjacent**  **genes^d^** | **Orientation^e^** | **5’ end^f^** | **3’ end^f^** | **454**  **control coIP^g^** | **454**  **Hfq coIP^h^** | **Enrichment^i^** | **Northern^j^** |
| --- | --- | --- | --- | --- | --- | --- | --- | --- | --- | --- |
| **STnc10** | - | V | STM0038/*nhaA* | → ← → | 46114 | 46050 | 0 | 0 |  | np |
| **STnc20** | - | V | STM0042/*rpsT* | ← → ← | 51926 | 52260 | 1 | 2 | 2.0 | np |
| **STnc30** | - | V | *lytB*/STM005 | → → → | 58792 | 58923 | 1 | 0 |  | np |
| **STnc470** | - | IV | STM0081/STM0082 | → ← ← | 94548 | 94770 | 0 | 70 | ≥70.0 | ~1250nt |
| ***sgrS*** | *ryaA* | I | *yabN/leuD* | ← → ← | 128574 | 128812 | 3 | 61 | 20.3 |  |
| **STnc40** | - | V | *secA/mutT* | → → → | 161464 | 161537 | 0 | 0 |  | np |
| **STnc50** | - | V | *lpdA*/STM0155 | → ← → | 182539 | 182458 | 0 | 0 |  | np |
| **STnc60** | - | V | *fhuB/stfA* | → ← → | 230277 | 230063 | 0 | 0 |  | np |
| ***isrA*** | - | II | STM0294.ln/STM0295 | → → → | 339338 | 339760 | 0 | 0 |  |  |
| ***sroB*** | *rybC* | I | *ybaK/ybaP* | ← → ← | 556005 | 556085 | 27 | 1530 | 56.7 |  |
| **STnc480** | - | IV | *glxK/ylbA* | → ← ← | 587848 | 587926 | 4 | 74 | 18.5 | nd |
| **STnc70** | - | V | *dsbG/ahpC* | ← → → | 670157 | 670305 | 5 | 7 | 1.4 | np |
| ***sroC*** | - | I | *gltJ/gltI* | ← ← ← | 728913 | 728761 | 26 | 898 | 34.5 |  |
| ***rybB*** | p25 | III | STM0869/STM0870 | → ← ← | 942632 | 942554 | 3 | 103 | 34.3 |  |
| **STnc80** | - | V | STM0897/STM0898 | ← → ← | 967580 | 967900 | 0 | 0 |  | np |
| **STnc90** | - | V | STM0903/STM0904 | → → ← | 974284 | 974363 | 0 | 0 |  | np |
| **STnc100** | - | V | STM0904/STM0905 | ← → → | 975011 | 975224 | 0 | 0 |  | np |
| **STnc110** | - | V | STM0905/STM0906 | → → → | 976578 | 976765 | 0 | 0 |  | np |
| **STnc120** | - | V | STM0929/*orfB* | ← ← → | 1004777 | 1004432 | 0 | 0 |  | np |
| **STnc490^k^** | - | IV | *clpA/tnpA_1* | → ← → | 1024975 | 1025165 | 75 | 385 | 5.1 | ~85nt |
| **STnc130** | - | V | *serS/dmsA* | → ← → | 1045232 | 1045098 | 0 | 0 |  | nd |
| ***isrB-1*** | - | II | *sbcA*/STM1010 | ← → ← | 1104179 | 1104266 | 2 | 4 | 2.0 |  |
| **STnc140** | - | V | STM1025/STM1026 | ← → ← | 1113681 | 1113750 | 0 | 0 |  | np |
| **STnc500** | - | IV | STM1127/STM1128 | ← ← ← | 1216157 | 1216440 | 7 | 84 | 12.0 | ~65nt |
| ***sraB*** | *pke2* | I | *yceF/yceD* | ← → → | 1275071 | 1275236 | 0 | 0 |  |  |
| **STnc640** | - | IV | *icdA*/STM1239 | → → → | 1325636 | 1326082 | 0 | 10 | ≥10.0 | ~1500nt |
| **STnc150** | - | V | *icdA*/STM1239 | → ← → | 1325914 | 1325649 | 0 | 1 | ≥1.0 | ~90nt |
| ***isrC*** | - | II | *envF/msgA* | ← → ← | 1329145 | 1329432 | 0 | 1 | ≥1.0 |  |
| **STnc510** | - | IV | STM1245/*pagC* | → → → | 1331440 | 1332250 | 4 | 28 | 7.0 | nd |
| **STnc520** | - | IV | STM1248/STM1249 | → ← ← | 1332809 | 1334044 | 12 | 100 | 8.3 | ~80nt |
| **STnc160** | - | V | STM1262/STM1263 | → ← → | 1345782 | 1345732 | 0 | 0 |  | np |
| ***isrD*** | - | II | STM1261/STM1263 | → ← → | 1345788 | 1345738 | 0 | 0 |  |  |
| ***ryhB-2*** | *isrE* | II | STM1273/*yeaQ* | → ← → | 1352987 | 1352875 | 0 | 0 |  |  |
| **STnc530** | - | IV | *yeaJ/yeaH* | → ← → | 1359779 | 1360418 | 2 | 15 | 7.5 | nd |
| **STnc540** | - | IV | *himA/btuC* | → → → | 1419369 | 1419570 | 7 | 23 | 3.3 | ~85nt |
| ***rprA*** | IS083 | I | *ydik/ydil* | ← ← ← | 1444938 | 1444832 | 37 | 286 | 7.7 |  |
| ***rydB*** | tpe7, IS082 | I | *ydiH*/STM1368 | → → ← | 1450415 | 1450519 | 4 | 10 | 2.5 |  |
| **STnc550** | - | IV | *purR/sodB* | ← → ← | 1508946 | 1509412 | 6 | 10 | 1.7 | nd |
| **STnc570^l^** | *yneM*, small ORF | IV | *ydeI/ydeE* | → ← ← | 1593723 | 1594413 | 2 | 21 | 10.5 | ~190nt |
| **STnc560** | *hbrC* | IV | *ydeI/ydeE* | → → ← | 1593723 | 1594413 | 10 | 290 | 29.0 | ~90nt |
| **STnc170** | - | V | STM1528/STM1530 | ← ← → | 1606116 | 1605784 | 0 | 0 |  | np |
| ***isrF*** | - | II | STM1552/STM1554 | → ← ← | 1630160 | 1629871 | 1 | 0 |  |  |
| ***rydC*** | IS067 | I | STM1638/*cybB* | → → ← | 1729673 | 1729738 | 5 | 245 | 49.0 |  |
| ***micC*** | IS063, tke8 | III | *nifJ/ynaF* | → ← → | 1745786 | 1745678 | 0 | 15 | ≥15.0 |  |
| **STnc580** | - | IV | *dbpA*/STM1656 | ← ← ← | 1749662 | 1750147 | 11 | 311 | 28.3 | ~100nt |
| **STnc180** | - | V | *acnA/cysB* | ← ← ← | 1807776 | 1807565 | 1 | 5 | 5.0 | ~2000nt |
| **STnc190** | - | V | STM1841/*kdgR* | → → ← | 1937518 | 1937652 | 1 | 12 | 12.0 | ~500nt |
| ***ryeB*** | tpke79 | I | STM1871/STM1872 | → ← ← | 1968155 | 1968053 | 24 | 653 | 27.2 |  |
| **STnc200** | - | V | *edd/zwf* | ← ← ← | 1979598 | 1979550 | 0 | 3 | ≥3.0 | nd |
| **STnc210** | - | V | *yecA*/STM1939 | ← → ← | 2032404 | 2032580 | 0 | 0 |  | np |
| ***dsrA*** | - | I | *yodD/yedP* | → ← → | 2068736 | 2068649 | 6 | 149 | 24.8 |  |
| ***rseX*** | - | I | STM1994/*ompS* | ← → → | 2077175 | 2077269 | 0 | 3 | ≥3.0 |  |
| **STnc220** | - | V | *ompS/cspB* | → ← ← | 2079068 | 2078990 | 0 | 8 | ≥8.0 | nd |
| **STnc230** | - | V | *pocR/pduF* | ← → ← | 2115370 | 2115452 | 0 | 0 |  | np |
| **STnc240** | - | V | *yeeF/yeeY* | ← ← ← | 2147409 | 2147333 | 0 | 1 | ≥1.0 | np |
| ***ryeC*** | tp11 | I | *yegD*/STM2126 | → → → | 2213871 | 2214016 | 42 | 72 | 1.7 |  |
| ***cyaR*** | *ryeE* | III | *yegQ*/STM2137 | → → → | 2231130 | 2231216 | 31 | 659 | 21.3 |  |
| ***isrG*** | - | II | STM2243/STM2244 | ← → → | 2344732 | 2345013 | 0 | 0 |  |  |
| ***micF*** | - | III | *ompC/yojN* | ← → → | 2366913 | 2367005 | 0 | 11 | ≥11.0 |  |
| ***isrH-2*** | - | II | *glpC*/STM2287 | → ← → | 2394582 | 2394303 | 0 | 0 |  |  |
| ***isrH-1*** | - | II | *glpC*/STM2287 | → ← → | 2394753 | 2394303 | 0 | 0 |  |  |
| **STnc250^l^** | *ypfM*, small ORF | V | *acrD/yffB* | → ← → | 2596882 | 2596789 | 6 | 24 | 4.0 | ~220nt |
| ***ryfA*** | tp1 | I | STM2534/*sseB* | → → ← | 2674934 | 2675228 | 3 | 6 | 2.0 |  |
| ***glmY*** | tke1, *sroF* | I | *yfhK/purG* | ← ← ← | 2707847 | 2707664 | 20 | 92 | 4.6 |  |
| ***isrI*** | - | II | STM2614/STM2616 | → ← ← | 2761576 | 2761329 | 0 | 2 | ≥2.0 |  |
| ***isrJ*** | - | II | STM2614/STM2616 | → ← ← | 2762031 | 2761957 | 1 | 0 |  |  |
| ***isrK*** | - | II | STM2616/STM2617 | ← ← ← | 2762867 | 2762791 | 0 | 0 |  |  |
| ***isrB-2*** | - | II | STM2631/*sbcA* | → ← → | 2770965 | 2770872 | 0 | 0 |  |  |
| ***isrL*** | - | II | *smpB*/STM2690 | → ← → | 2839399 | 2839055 | 0 | 0 |  |  |
| ***isrM*** | - | II | STM2762/STM2763 | ← → → | 2905050 | 2905378 | 0 | 0 |  |  |
| ***isrN*** | - | II | STM2764/STM2765 | ← → ← | 2906925 | 2907067 | 0 | 0 |  |  |
| **STnc260** | - | V | STM2816/*luxS* | ← → ← | 2966073 | 2966247 | 0 | 0 |  | np |
| ***micA*** | *sraD* | I | *luxS/gshA* | ← → ← | 2966853 | 2966926 | 1 | 128 | 128.0 |  |
| **STnc590** | - | IV | *avrA/sprB* | ← ← ← | 3010807 | 3010966 | 3 | 27 | 9.0 | nd |
| **STnc600** | - | IV | *hilD/hilA* | → → → | 3018766 | 3019855 | 3 | 68 | 22.7 | nd |
| ***invR*** | STnc270 | III | *invH*/STM 2901 | → → → | 3044924 | 3045014 | 113 | 3236 | 28.6 |  |
| ***csrB*** | - | III | *yqcC/syd* | ← ← ← | 3117059 | 3116697 | 69 | 67 |  |  |
| ***gcvB*** | IS145 | III | *gcvA/ygdI* | ← → ← | 3135317 | 3135522 | 12 | 402 | 33.5 |  |
| ***omrA*** | *rygB* | III | *aas/galR* | ← ← → | 3170208 | 3170122 | 0 | 51 | ≥51.0 |  |
| ***omrB*** | *t59, rygA, sraE* | III | *aas/galR* | ← ← → | 3170408 | 3170322 | 1 | 52 | 52.0 |  |
| **STnc280** | - | V | *kduI/yqeF* | ← → ← | 3179540 | 3179622 | 0 | 1 | ≥1.0 | np |
| **STnc290** | - | V | *tnpA_4*/STM3033 | ← ← ← | 3194996 | 3194914 | 2 | 72 | 36.0 | ~85nt |
| ***isrO*** | - | II | STM3038/STM3039 | ← → → | 3198380 | 3198580 | 0 | 0 |  |  |
| ***ssrS*** | - | I | *ygfE/ygfA* | → → → | 3222098 | 3222280 | 836 | 451 |  |  |
| ***rygC*** | t27 | I | *ygfA/serA* | → → ← | 3222913 | 3223065 | 14 | 17 | 1.2 |  |
| **STnc300** | - | V | STM3123/STM3124 | ← ← → | 3283965 | 3283807 | 0 | 0 |  | np |
| ***rygD*** | tp8, C0730 | I | *yqiK/rfaE* | → ← ← | 3362474 | 3362327 | 17 | 104 | 6.1 |  |
| ***sraF*** | tpk1, IS160 | I | *ygjR/ygjT* | → → → | 3392069 | 3392261 | 0 | 25 | ≥25.0 |  |
| **STnc310** | - | V | *ygjT/ygjU* | → ← → | 3393327 | 3393267 | 0 | 0 |  | np |
| **STnc320** | - | V | *yhaO/tdcG* | ← → ← | 3404895 | 3404949 | 0 | 1 | ≥1.0 | np |
| **STnc610** | - | IV | *yhbC/metY* | ← ← ← | 3458296 | 3458578 | 1 | 19 | 19.0 | ~1250nt |
| **STnc330** | - | V | *greA/dacB* | ← ← → | 3468553 | 3468497 | 1 | 12 | 12.0 | ~1500nt |
| ***sraH*** | *ryhA* | I | *yhbL/arcB* | ← → ← | 3490383 | 3490500 | 55 | 2292 | 41.7 |  |
| **STnc340** | - | V | *tnpA_5/yhfL* | ← ← → | 3635884 | 3635756 | 0 | 0 |  | nd |
| ***ryhB-1*** | *sraI*, IS176 | I | *yhhX/yhhY* | ← ← → | 3715495 | 3715401 | 0 | 2 | ≥2.0 |  |
| **STnc350** | - | V | *uspA/yhiP* | → ← → | 3761440 | 3761373 | 0 | 0 |  | nd |
| **STnc360** | - | V | *yhjB/yhjC* | ← → → | 3780254 | 3780402 | 0 | 0 |  | np |
| **STnc370** | - | V | STM3654/*glyS* | ← → ← | 3839688 | 3839758 | 0 | 0 |  | np |
| **STnc380** | - | V | STM3691/*lldP* | → ← → | 3885736 | 3885629 | 0 | 0 |  | np |
| **STnc390** | - | V | *yibD/tdh* | ← ← ← | 3902653 | 3902594 | 0 | 0 |  | nd |
| ***istR-1*** | - | VI | *ilvB/emrD* | ← ← → | 3998147 | 3998018 | 0 | 0 |  | ~75nt |
| ***istR-2*** | - | VI | *ilvB/emrD* | ← ← → | 3998147 | 3998018 | 0 | 0 |  | ~140nt |
| **STnc400** | - | V | STM3844/STM3845 | → → → | 4051145 | 4051340 | 112 | 42 |  | ~55nt |
| **STnc410** | - | V | *glmU*/STM3863 | ← → ← | 4072507 | 4072730 | 0 | 0 |  | np |
| ***glmZ*** | k19, *ryiA*, *sraJ* | I | *yifK/hemY* | → → ← | 4141650 | 4141854 | 20 | 196 | 9.8 |  |
| ***spf*** | *-* | I | *polA/yihA* | → → ← | 4209066 | 4209175 | 2 | 33 | 16.5 |  |
| ***csrC*** | *sraK*, *ryiB*, *tpk2* | III | *yihA/yihI* | ← → → | 4210157 | 4210400 | 63 | 64 |  |  |
| **STnc420** | - | V | *yiiG*/STM4041 | → ← ← | 4251539 | 4251480 | 0 | 0 |  | np |
| ***isrP*** | - | II | STM4097/STM4098 | ← → ← | 4306719 | 4306866 | 0 | 2 | ≥2.0 |  |
| ***oxyS*** | - |  | *argH/oxyR* | → ← → | 4342986 | 4342866 | 0 | 10 | 10.0 |  |
| **STnc430** | - | V | *pgi/yjbE* | → ← → | 4442059 | 4441898 | 0 | 0 |  | np |
| **STnc620** | - | IV | *ssb*/STM4257 | → → → | 4476817 | 4477856 | 4 | 41 | 10.3 | nd |
| ***sraL*** | *ryjA* | III | *soxR*/STM4267 | → ← → | 4505010 | 4504870 | 0 | 0 |  |  |
| **STnc630** | - | IV | *proP/basS* | → → ← | 4532473 | 4532638 | 1 | 27 | 27.0 | nd |
| **STnc440** | - | V | STM4310/*tnpA_6* | → → → | 4559193 | 4559277 | 9 | 456 | 50.7 | ~85nt |
| **STnc450** | - | V | *ytfL/msrA* | ← ← ← | 4645134 | 4645079 | 0 | 0 |  | np |
| **STnc460** | - | V | STM4503/STM4504 | → ← → | 4758332 | 4758187 | 0 | 0 |  | np |
| ***isrQ*** | - | II | STM4508/STM4509 | ← → → | 4762997 | 4763158 | 0 | 0 |  |  |

^a^ Gene names of *Salmonella* sRNAs that have been experimentally proven here, and in previous studies. Method of identification is given in the third column. sRNA names follow *Salmonella* and/or *E. coli* nomenclature referenced in [7,8,9], except STnc470 to STnc630, which have been newly predicted in this study.

^b^ Alternative sRNA IDs. References in [7,8,9].

^c^ Evidence for sRNAs in *Salmonella*.

(I) Conserved sRNA found in *Salmonella* cDNA libraries, and previously shown to be expressed in *E. coli* (relevant ref. in [7]; Table 1).

(II) sRNA previously predicted and validated on Northern blots in *Salmonella* by [9].

(III) sRNA previously validated on Northern blots in *Salmonella* [1,7,10,11,12,13,14,15].

(IV) sRNA predicted through cDNA sequencing and validated by Northern blot analysis in this study.

(V) sRNA previously predicted by [11].

(VI) IstR sRNAs [2] were not recoverd in cDNA sequences but their expression in *Salmonella* validated by northern blot analysis in this study (Fig. S5).

^d^ Flanking genes of the intergenic region in which the sRNA candidate is located.

^e^ Orientation of sRNA candidate (middle) and flanking genes (→ and ← denote location of a gene on the

clockwise or the counterclockwise strand of the *Salmonella* chromosome).

^f^ Genomic location of sRNA candidate gene according to the *Salmonella typhimurium* LT2 genome. For

STnc470 through STnc640 start and end of the entire intergenic region are given.

^g^ Out of 145,873 sequences in total.

^h^ Out of 122,326 sequences in total.

^i^ Enrichment factor calculated by the number of blastable reads from Hfq coIP over control coIP.

^j^ Denotes verification on Northern blot in this study for new RNA transcripts; the estimated size is given in nucleotides (np = not probed; nd = no detectable transcript).

^k^ The cDNA reads map antisense internally of the IS200 element. Based on sequence identity they map to all IS200 elements (*tnpA_1* to *tnpA_6*).

^l^ STnc250 and STnc570 contain small ORFs annotated as *ypfM* or *yneM*, respectively, in *E. coli* [16].
