# Supplementary material for: Deep Sequencing Analysis of Small Noncoding RNA and mRNA Targets of the Global Post-Transcriptional Regulator, Hfq
Source: PLoS Genet. 2008 Aug 22;4(8):e1000163. doi: 10.1371/journal.pgen.1000163 (PMC2515195; doi:10.1371/journal.pgen.1000163)
Supplement: Table S4 — mRNAs in Hfq CoIP identified by ≥10 of 170,000 inserts in pyrosequencing data. (0.81 MB DOC) [file pgen.1000163.s009.doc]

Table S4: mRNAs in Hfq CoIP identified by ≥10 of 170,000 inserts in pyrosequencing data

| **STM number** | **Gene name^a^** | **Number of**  **inserts in**  **control coIP^b^** | **Number of**  **inserts in**  **Hfq coIP^c^** | **Product^d^** |
| --- | --- | --- | --- | --- |
| STM4261 |  | 254 | 1042 | putative inner membrane protein |
| STM2665 | yfiA | 72 | 648 | ribosome stabilization factor |
| STM1377 | lpp | 168 | 608 | murein lipoprotein |
| STM4087 | glpF | 40 | 570 | glycerol diffusion |
| STM1959 | fliC | 248 | 547 | flagellar biosynthesis protein |
| STM2874 | prgH | 73 | 415 | needle complex inner membrane protein |
| STM2267 | ompC | 63 | 385 | outer membrane protein C precursor |
| STM2882 | sipA | 36 | 354 | secreted effector protein |
| STM2885 | sipB | 126 | 335 | translocation machinery component |
| STM4326 | aspA | 79 | 328 | aspartate ammonia-lyase |
| STM2925 | nlpD | 30 | 300 | lipoprotein |
| STM4086 | glpK | 115 | 278 | glycerol kinase |
| STM2883 | sipD | 34 | 269 | translocation machinery component |
| STM0739 | sucD | 14 | 261 | succinyl-CoA synthetase alpha subunit |
| STM1572 | ompD | 76 | 246 | putative outer membrane porin precursor |
| STM2898 | invG | 16 | 226 | outer membrane secretin precursor |
| STM2879 | sicP | 6 | 224 | secretion chaparone |
| STM2283 | glpT | 30 | 221 | sn-glycerol-3-phosphate transport protein |
| STM1091 | sopB | 23 | 216 | secreted effector protein |
| STM1732 | ompW | 28 | 206 | outer membrane protein W precursor |
| STM0451 | hupB | 14 | 198 | DNA-binding protein HU-beta |
| STM2871 | prgK | 46 | 198 | needle complex inner membrane lipoprotein |
| STM2884 | sipC | 96 | 192 | translocation machinery component |
| STM4406.S | ytfK | 6 | 191 | putative cytoplasmic protein |
| STM2867 | hilC | 3 | 187 | invasion regulatory protein |
| STM2869 | orgB | 8 | 182 | needle complex export protein |
| STM2878 | sptP | 20 | 177 | protein tyrosine phosphatase/GTPase activating protein |
| STM2894 | invC | 14 | 175 | type III secretion system ATPase |
| STM2875 | hilD | 23 | 174 | invasion protein regulatory protein |
| STM2284 | glpA | 57 | 149 | sn-glycerol-3-phosphate dehydrogenase large subunit |
| STM3526 | glpD | 39 | 147 | sn-glycerol-3-phosphate dehydrogenase |
| STM2886 | sicA | 23 | 146 | secretion chaperone |
| STM3138 |  | 19 | 143 | putative methyl-accepting chemotaxis protein |
| STM2896 | invA | 19 | 142 | needle complex export protein |
| STM0833 | ompX | 6 | 137 | outer membrane protein X |
| STM2899 | invF | 18 | 129 | invasion regulatory protein |
| STM2924 | rpoS | 19 | 129 | RNA polymerase sigma factor |
| STM0629 | cspE | 9 | 125 | cold shock protein E |
| STM2285 | glpB | 33 | 119 | anaerobic glycerol-3-phosphate dehydrogenase subunit B |
| STM0736 | sucA | 42 | 110 | 2-oxoglutarate dehydrogenase |
| STM2445 | ucpA | 5 | 105 | short chain dehydrogenase |
| STM1070 | ompA | 77 | 102 | putative hydrogenase membrane component precurosr |
| STM2282 | glpQ | 31 | 98 | periplasmic glycerophosphodiester phosphodiesterase |
| STM3500 | pckA | 33 | 96 | phosphoenolpyruvate carboxykinase |
| STM3649 | cspA | 7 | 94 | major cold shock protein |
| STM0748 | tolB | 7 | 90 | translocation protein TolB precursor |
| STM1782 | ychH | 10 | 90 | putative inner membrane protein |
| STM3420 | secY | 30 | 87 | preprotein translocase SecY |
| STM1171 | flgN | 8 | 80 | putative FlgK/FlgL export chaperone |
| STM0737 | sucB | 12 | 79 | dihydrolipoamide acetyltransferase |
| STM2891 | spaO | 8 | 77 | type III secretion protein |
| STM2892 | invJ | 10 | 75 | needle length control protein |
| STM3281 | nlpI | 19 | 75 | lipoprotein |
| STM1887 | yebK | 0 | 74 | putative transcriptional regulator |
| STM4360 | miaA | 5 | 74 | tRNA delta(2)-isopentenylpyrophosphate transferase |
| STM4260 |  | 16 | 71 | predicted cation efflux pump |
| STM0738 | sucC | 19 | 70 | succinyl-CoA synthetase subunit beta |
| STM1919 | cheM | 12 | 70 | methyl accepting chemotaxis protein II |
| STM0740 | cydA | 21 | 69 | cytochrome d terminal oxidase polypeptide subunit I |
| STM2870 | orgA | 16 | 69 | needle complex assembly protein |
| STM3150 | hypO | 2 | 68 | putative Ni/Fe hydrogenase small subunit |
| STM3630 | dppA | 6 | 68 | dipeptide transport protein |
| STM2328 | nuoA | 3 | 66 | NADH dehydrogenase alpha subunit |
| STM0945 | clpA | 13 | 65 | ATP-binding subunit of serine protease |
| STM1923 | motA | 9 | 65 | flagellar motor protein |
| STM2897 | invE | 14 | 65 | invasion protein |
| STM1921 | cheA | 16 | 62 | chemotaxis sensory histidine protein kinase |
| STM2314 |  | 20 | 61 | putative chemotaxis signal transduction protein |
| STM2895 | invB | 11 | 60 | secretion chaperone |
| STM1230 | phoQ | 8 | 58 | sensor kinase protein |
| STM4262 |  | 8 | 58 | putative ABC-type bacteriocin/lantibiotic exporter |
| STM1751 | hns | 13 | 57 | DNA-binding protein HLP-II |
| STM4336 | ecnB | 3 | 57 | putative entericidin B precursor |
| STM1090 | pipC | 10 | 55 | pathogenicity island-encoded protein C |
| STM2957 | rumA | 4 | 55 | 23S rRNA (uracil-5-)-methyltransferase |
| STM1922 | motB | 10 | 54 | flagellar motor protein |
| STM2868 | orgC | 7 | 54 | putative cytoplasmic protein |
| STM2872 | prgJ | 8 | 54 | needle complex minor subunit |
| STM4232 | malM | 7 | 54 | periplasmic protein precursor |
| STM1184 | flgL | 8 | 53 | flagellar hook-associated protein |
| STM4258 |  | 10 | 53 | putative methyl-accepting chemotaxis protein |
| STM4362 | hflX | 7 | 53 | putative GTP-ase |
| STM1066 | rmf | 4 | 52 | ribosome modulation factor |
| STM1431 | sodB | 21 | 52 | superoxide dismutase |
| STM3604 |  | 8 | 52 | putative inner membrane protein |
| STM1765 | narK | 11 | 50 | nitrite extrusion protein |
| STM2261 | napF | 13 | 50 | electron transfer protein |
| STM3053 | gcvP | 30 | 50 | glycine dehydrogenase |
| STM3466 | crp | 4 | 50 | catabolite activator protein |
| STM0600 | cstA | 19 | 49 | carbon starvation protein |
| STM1285 | yeaG | 16 | 49 | putative serine protein kinase |
| STM1311 | osmE | 11 | 49 | transcriptional activator |
| STM3515 | malT | 14 | 49 | transcriptional regulator MalT |
| STM0960 | ftsK | 8 | 47 | cell division protein |
| STM1203 | ptsG | 8 | 47 | glucose-specific IIBC component |
| STM3711 | rfaF | 3 | 47 | ADP-heptose-LPS heptosyltransferase 1 |
| STM4361 | hfq | 5 | 47 | RNA-binding protein Hfq |
| STM0943 | cspD | 5 | 46 | stress response protein |
| STM1183 | flgK | 5 | 46 | flagellar hook-associated protein |
| STM2065 | phsA | 3 | 46 | thiosulfate reductase precursor |
| STM2088 | rfbX | 1 | 46 | putative O-antigen transferase |
| STM2873 | prgI | 15 | 46 | needle complex major subunit |
| STM4151 | rplJ | 6 | 46 | 50S ribosomal protein L10 |
| STM4325 | dcuA | 19 | 45 | anaerobic C4-dicarboxylate transporter |
| STM0617 | rna | 3 | 44 | RNase I |
| STM1231 | phoP | 3 | 44 | response regulator |
| STM2889 | spaQ | 2 | 44 | needle complex export protein |
| STM3803 | yidF | 5 | 44 | putative cytoplasmic protein |
| STM4152 | rplL | 2 | 44 | 50S ribosomal protein L7/L12 |
| STM0439 | cyoE | 16 | 43 | protoheme IX farnesyltransferase |
| STM2001 | yeeI | 6 | 43 | putative inner membrane protein |
| STM2893 | invI | 10 | 43 | needle complex assembly protein |
| STM2876 | hilA | 4 | 42 | invasion protein transcriptional activator |
| STM2881 | iacP | 6 | 42 | acyl carrier protein |
| STM3197 | glgS | 2 | 42 | glycogen synthesis protein GlgS |
| STM0158 | acnB | 21 | 41 | aconitate hydratase |
| STM0741 | cydB | 20 | 41 | cytochrome d terminal oxidase polypeptide subunit II |
| STM2286 | glpC | 27 | 41 | sn-glycerol-3-phosphate dehydrogenase K-small subunit |
| STM0039 | nhaA | 2 | 40 | Na+/H antiporter |
| STM1336 | rplT | 10 | 40 | 50S ribosomal protein L20 |
| STM1917 | cheB | 16 | 40 | chemotaxis-specific methylesterase |
| STM2081 | gnd | 3 | 40 | 6-phosphogluconate dehydrogenase |
| STM2082 | rfbP | 3 | 40 | undecaprenol-phosphate galactosephosphotransferase/O-antigen transferase |
| STM2326 | nuoC | 7 | 40 | NADH dehydrogenase I chain C/D |
| STM2827 | alaS | 3 | 40 | alanyl-tRNA synthetase |
| STM3106 | ansB | 3 | 40 | periplasmic L-asparaginase II |
| STM3807 | yidE | 1 | 40 | hypothetical protein |
| STM0457 | cof | 2 | 39 | putative hydrolase |
| STM1960 | fliD | 14 | 39 | flagellar hook-associated protein |
| STM3404 | smg | 9 | 39 | hypothetical protein |
| STM4060 | cpxP | 1 | 39 | periplasmic repressor |
| STM0831 | dps | 9 | 38 | DNA protection during starvation conditions |
| STM1057 | pepN | 5 | 37 | aminopeptidase N |
| STM1838 | yobF | 7 | 37 | putative cytoplasmic protein |
| STM3359 | mdh | 14 | 37 | malate dehydrogenase |
| STM4315 |  | 0 | 37 | putative DNA-binding protein |
| STM0994 | mukB | 4 | 36 | condesin subunit B |
| STM1746.S | oppA | 5 | 36 | oligopeptide transport protein |
| STM1841 |  | 0 | 36 | hypothetical protein |
| STM2660 | clpB | 46 | 36 | ATP-dependent protease |
| STM3884 | rbsB | 6 | 36 | D-ribose transport protein |
| STM4305.S |  | 3 | 36 | putative anaerobic dimethylsulfoxide reductase subunit A |
| STM0129 | murC | 4 | 35 | UDP-N-acetylmuramate--L-alanine ligase |
| STM1165 | grxB | 5 | 35 | glutaredoxin 2 |
| STM1386 | ttrS | 6 | 35 | sensory histidine kinase |
| STM1742 | oppF | 3 | 35 | oligopeptide transport protein |
| STM1855 | sopE2 | 4 | 35 | type III-secreted effector protein |
| STM3162 | yghB | 2 | 35 | hypothetical protein |
| STM1238 | icdA | 17 | 34 | isocitrate dehydrogenase |
| STM1333 | thrS | 10 | 34 | threonyl-tRNA synthetase |
| STM1918 | cheR | 8 | 34 | glutamate methyltransferase |
| STM2323.S | nuoG | 15 | 34 | NADH dehydrogenase gamma subunit |
| STM3419 | rpmJ | 9 | 34 | 50S ribosomal protein L36 |
| STM3430 | rplN | 10 | 34 | 50S ribosomal protein L14 |
| STM3445 | tuf | 37 | 34 | elongation factor Tu |
| STM4259 |  | 7 | 34 | putative ABC exporter outer membrane component |
| STM0013 | dnaJ | 2 | 33 | heat shock protein |
| STM1925 | flhD | 1 | 33 | transcriptional activator FlhD |
| STM1956 | fliA | 11 | 33 | flagellar biosynthesis sigma factor FliA |
| STM2956 | relA | 5 | 33 | (p)ppGpp synthetase I |
| STM3700 | gpsA | 1 | 33 | NAD(P)H-dependent glycerol-3-phosphate dehydrogenase |
| STM4154 | rpoC | 37 | 33 | DNA-directed RNA polymerase beta' subunit |
| STM4237 | lexA | 6 | 33 | LexA repressor |
| STM0964 | dmsA | 7 | 32 | anaerobic dimethyl sulfoxide reductase subunit A |
| STM1085 | yccA | 4 | 32 | putative transport protein |
| STM1164 | yceB | 3 | 32 | putative outer membrane lipoprotein |
| STM3592 | yhiP | 4 | 32 | putative peptide transport protein |
| STM4562 |  | 3 | 32 | putative inner membrane protein |
| STM0614 | ybdQ | 9 | 31 | putative universal stress protein |
| STM1837 | cspC | 11 | 31 | cold shock protein |
| STM3183 | icc | 4 | 31 | cyclic 3',5'-adenosine monophosphate phosphodiesterase |
| STM3577 | tcp | 11 | 31 | methyl-accepting transmembrane citrate/phenol chemoreceptor |
| STM0749 | pal | 7 | 30 | peptidoglycan-associated lipoprotein precursor |
| STM1583 |  | 1 | 30 | putative cytoplasmic protein |
| STM1955 | fliZ | 3 | 30 | putative FliA-regulator |
| STM2432 | ptsI | 7 | 30 | PEP-protein phosphotransferase |
| STM3006 | ygdQ | 3 | 30 | putative transport protein |
| STM4582 | slt | 0 | 30 | soluble lytic murein transglycosylase |
| STM0093 | imp | 2 | 29 | organic solvent tolerance protein precursor |
| STM0224 | yaeT | 5 | 29 | putative outer membrane protein precursor |
| STM0687 | ybfM | 2 | 29 | putative outer membrane protein |
| STM0688 | ybfN | 1 | 29 | putative lipoprotein |
| STM1888 | pykA | 6 | 29 | pyruvate kinase |
| STM3705 | yibP | 2 | 29 | hypothetical protein |
| STM0120 | mraW | 4 | 28 | S-adenosyl-methyltransferase |
| STM1172 | flgM | 10 | 28 | anti-FliA factor |
| STM1334.c | infC | 11 | 28 | translation initiation factor IF-3 |
| STM1996 | cspB | 0 | 28 | putative cold-shock protein |
| STM3918 | rfe | 2 | 28 | undecaprenyl-phosphate N-acetylglucosaminyltransferase |
| STM4153 | rpoB | 11 | 28 | DNA-directed RNA polymerase beta subunit |
| STM0472 | maa | 4 | 27 | maltose O-acetyltransferase |
| STM0800 | slrP | 9 | 27 | leucine-rich repeat protein |
| STM2301 | pqaB | 4 | 27 | putative melittin resistance protein |
| STM2532 |  | 3 | 27 | putative inner membrane lipoprotein |
| STM3004 | ygdP | 3 | 27 | dinucleoside polyphosphate hydrolase |
| STM3055 | gcvT | 7 | 27 | aminomethyltransferase |
| STM3865 | atpD | 17 | 27 | ATP synthase subunit B |
| STM4243 | yjbN | 1 | 27 | hypothetical protein |
| STM4561 | osmY | 7 | 27 | hyperosmotically-inducible periplasmic protein |
| STM0130 | ddl | 6 | 26 | D-alanylalanine synthetase |
| STM0226 | lpxD | 3 | 26 | UDP-3-O-[3-hydroxymyristoyl] glucosamine N-acyltransferase |
| STM0366 | yahO | 5 | 26 | putative periplasmic protein |
| STM0734 | sdhA | 25 | 26 | succinate dehydrogenase catalytic subunit |
| STM1290 | gapA | 11 | 26 | glyceraldehyde-3-phosphate dehydrogenase |
| STM1924.S | flhC | 3 | 26 | flagellar transcriptional activator |
| STM2368 | truA | 0 | 26 | tRNA pseudouridine synthase A |
| STM3070 | epd | 3 | 26 | D-erythrose 4-phosphate dehydrogenase |
| STM3216 |  | 6 | 26 | putative methyl-accepting chemotaxis protein |
| STM4037 | fdoG | 26 | 26 | formate dehydrogenase alpha subunit |
| STM4076 | ydeZ | 6 | 26 | putative sugar transport protein |
| STM0122 | ftsI | 7 | 25 | division specific transpeptidase |
| STM0125 | mraY | 1 | 25 | phospho-N-acetylmuramoyl-pentapeptide-transferase |
| STM1196 | acpP | 10 | 25 | acyl carrier protein |
| STM1317 | celG | 0 | 25 | hypothetical protein |
| STM1318 | katE | 4 | 25 | hydroperoxidase HPII |
| STM1324 |  | 5 | 25 | putative cytoplasmic protein |
| STM1338 | pheT | 13 | 25 | phenylalanyl-tRNA synthetase beta subunit |
| STM1661 | ydaA | 18 | 25 | putative universal stress protein |
| STM1801 | ycgO | 2 | 25 | cell volume regulation protein CvrA |
| STM1938 | yecA | 7 | 25 | putative metal-binding protein |
| STM1947 | uvrY | 2 | 25 | response regulator |
| STM2318 | nuoL | 6 | 25 | NADH dehydrogenase subunit L |
| STM2983 | ygdI | 6 | 25 | putative lipoprotein |
| STM3003 | ptsP | 8 | 25 | transcriptional regulator |
| STM3403 | yrdD | 4 | 25 | putative DNA topoisomerase |
| STM3616 | yhjL | 5 | 25 | tetratricopeptide repeat protein |
| STM3701 | secB | 5 | 25 | export protein SecB |
| STM3968 | udp | 8 | 25 | uridine phosphorylase |
| STM4343 | frdA | 15 | 25 | fumarate reductase |
| STM1349 | pps | 9 | 24 | phosphoenolpyruvate synthase |
| STM1601 | ugtL | 1 | 24 | hypothetical protein |
| STM2530 |  | 3 | 24 | putative anaerobic dimethylsulfoxide reductase |
| STM2888 | spaR | 2 | 24 | needle complex export protein |
| STM4170 | hupA | 12 | 24 | DNA-binding protein HU-alpha |
| STM4368 | vacB | 4 | 24 | putative exoribonuclease |
| STM0735 | sdhB | 8 | 23 | succinate dehydrogenase catalytic subunit |
| STM0863 | dacC | 2 | 23 | D-alanyl-D-alanine carboxypeptidase |
| STM1000 | asnS | 3 | 23 | asparaginyl-tRNA synthetase |
| STM1749 | adhE | 9 | 23 | iron-dependent alcohol dehydrogenase |
| STM1920 | cheW | 6 | 23 | chemotaxis docking protein |
| STM2083 | rfbK | 1 | 23 | phosphomannomutase |
| STM2327 | nuoB | 3 | 23 | NADH dehydrogenase beta subunit |
| STM2526 | ndk | 2 | 23 | nucleoside diphosphate kinase |
| STM2651 | yfiQ | 11 | 23 | putative acetyl-CoA synthetase |
| STM3591 | uspA | 14 | 23 | universal stress protein A |
| STM3702 | grxC | 2 | 23 | glutaredoxin 3 |
| STM3808.S | ibpB | 1 | 23 | small heat shock protein |
| STM3972 | aarF | 8 | 23 | putative ubiquinone biosynthesis protein UbiB |
| STM4257 |  | 5 | 23 | hypothetical protein |
| STM0186 | dksA | 12 | 22 | dnaK suppressor protein |
| STM0452 | cypD | 3 | 22 | peptidyl-prolyl isomerase |
| STM0730 | gltA | 16 | 22 | citrate synthase |
| STM1094 | pipD | 2 | 22 | pathogenicity island-encoded protein D |
| STM1249 |  | 5 | 22 | utative periplasmic protein |
| STM2091 | rfbG | 3 | 22 | CDP glucose 4,6-dehydratase |
| STM2296 | ais | 0 | 22 | aluminum-inducible protein |
| STM2780 | pipB2 | 3 | 22 | secreted effector protein |
| STM2890 | spaP | 2 | 22 | needle complex export protein |
| STM4410 | ytfN | 6 | 22 | putative periplasmic protein |
| STM1161.S | yceP | 1 | 21 | putative cytoplasmic protein |
| STM1190 | yceD | 9 | 21 | putative metal-binding protein |
| STM1283 | yeaJ | 2 | 21 | putative methyl-accepting chemotaxis protein |
| STM1286 | mipA | 6 | 21 | MltA-interacting protein A |
| STM1291 | yeaA | 1 | 21 | methionine sulfoxide reductase B |
| STM1683 | tyrR | 1 | 21 | transcriptional regulator |
| STM1795 |  | 9 | 21 | putative glutamic dehyrogenase-like protein |
| STM1916 | cheY | 3 | 21 | chemotaxis regulator |
| STM1941 |  | 0 | 21 | putative inner membrane protein |
| STM1945 | pgsA | 2 | 21 | phosphatidylglycerophosphate synthetase |
| STM2391 | fadL | 1 | 21 | outer membrane-bound fatty acid transporter |
| STM3147 | hybC | 3 | 21 | hydrogenase-2 large subunit |
| STM3282 | pnp | 4 | 21 | polynucleotide phosphorylase |
| STM3415 | rpoA | 11 | 21 | DNA-directed RNA polymerase alpha subunit |
| STM3417 | rpsK | 8 | 21 | 30S ribosomal protein S11 |
| STM4240 | yjbJ | 13 | 21 | putative cytoplasmic protein |
| STM4330 | groEL | 43 | 21 | chaperonin GroEL |
| STM0653 | ybeL | 3 | 20 | putative cytoplasmic protein |
| STM0732 | sdhC | 4 | 20 | succinate dehydrogenase cytochrome b556 large membrane subunit |
| STM1713 | cysB | 3 | 20 | transcriptional regulator for cysteine regulon |
| STM2035 | cbiA | 4 | 20 | cobyrinic acid a,c-diamide synthase |
| STM2322 | nuoH | 6 | 20 | NADH dehydrogenase subunit H |
| STM2533 | sseA | 4 | 20 | putative sulfurtransferase |
| STM3187 | ygiB | 0 | 20 | putative inner membrane protein |
| STM3538 | glgB | 10 | 20 | glycogen branching enzyme |
| STM3708 | tdh | 5 | 20 | L-threonine 3-dehydrogenase |
| STM3710 | rfaD | 9 | 20 | ADP-L-glycero-D-mannoheptose-6-epimerase |
| STM3900 | ilvL | 2 | 20 | ilvGEDA operon leader peptide |
| STM3996 | yihE | 1 | 20 | putative type II homoserine kinase |
| STM0088 | apaH | 2 | 19 | diadenosinetetraphosphatase |
| STM0124 | murF | 6 | 19 | D-alanine-D-alanine ligase |
| STM0128 | murG | 4 | 19 | N-acetylglucosaminyl transferase |
| STM0133 | ftsZ | 7 | 19 | cell division protein FtsZ |
| STM0449 | clpX | 10 | 19 | ATP-dependent protease ATP-binding subunit |
| STM1398 | sseB | 2 | 19 | translocation machinery component |
| STM1660.S | fnr | 4 | 19 | transcriptional regulator |
| STM1754 | ychK | 0 | 19 | putative phosphoesterase |
| STM1796 | treA | 6 | 19 | trehalase |
| STM1875 | yobA | 0 | 19 | putative copper resistance protein |
| STM2059 | yeeX | 1 | 19 | hypothetical protein |
| STM2184 | sanA | 3 | 19 | vancomycin sensitivity |
| STM2270 | rcsB | 0 | 19 | response regulator |
| STM2280 |  | 1 | 19 | putative permease |
| STM2433 | crr | 7 | 19 | glucose-specific PTS system enzyme IIA component |
| STM2681 | grpE | 1 | 19 | heat shock protein |
| STM2782 | mig-14 | 1 | 19 | putative transcriptional activator |
| STM3113 | nupG | 2 | 19 | nucleoside transport |
| STM3426 | rpsH | 6 | 19 | 30S ribosomal protein S8 |
| STM3986 | trkH | 3 | 19 | potassium transport protein |
| STM4331 | yjeI | 6 | 19 | putative outer membrane lipoprotein |
| STM4391 | rpsF | 1 | 19 | 30S ribosomal protein S6 |
| STM0132 | ftsA | 6 | 18 | cell division protein |
| STM0474 | ybaJ | 4 | 18 | putative cytoplasmic protein |
| STM0508 | ybbP | 2 | 18 | putative inner membrane protein |
| STM0750 | ybgF | 4 | 18 | putative periplasmic protein |
| STM0959 | lrp | 1 | 18 | leucine-responsive regulatory protein |
| STM1148.S | ymdC | 6 | 18 | putative phospholipase |
| STM1335 | rpmI | 5 | 18 | 50S ribosomal protein L35 |
| STM1602 | sifB | 0 | 18 | secreted effector protein |
| STM1745 | oppB | 1 | 18 | oligopeptide permease ABC transporter membrane component |
| STM1804.S | ycgB | 4 | 18 | putative cytoplasmic protein |
| STM1840 | yobG | 2 | 18 | putative inner membrane protein |
| STM1867 | pagK | 0 | 18 | PagK |
| STM1950 | sdiA | 3 | 18 | transcriptional regulator |
| STM2214 | spr | 2 | 18 | putative lipoprotein |
| STM2333 | yfbS | 1 | 18 | putative response regulator |
| STM2486 |  | 1 | 18 | putative inner membrane protein |
| STM2640 | rpoE | 2 | 18 | RNA polymerase sigma-70 factor |
| STM2675 | rimM | 3 | 18 | 16S rRNA-processing protein |
| STM2945 | sopD | 3 | 18 | secreted effector protein |
| STM3186 | tolC | 10 | 18 | outer membrane channel precursor protein |
| STM3229 | yqjD | 3 | 18 | putative inner membrane protein |
| STM3272 | yhbS | 4 | 18 | putative transport protein |
| STM3286 | infB | 4 | 18 | translation initiation factor IF-2 |
| STM3373 | mreC | 0 | 18 | rod shape-determining protein |
| STM3434 | rpsC | 20 | 18 | 30S ribosomal protein S3 |
| STM3611 | yhjH | 1 | 18 | hypothetical protein |
| STM3680 | aldB | 3 | 18 | aldehyde dehydrogenase B |
| STM3867 | atpA | 27 | 18 | ATP synthase subunit A |
| STM4078 | yneB | 9 | 18 | hypothetical protein |
| STM0134 | lpxC | 15 | 17 | UDP-3-O-[3-hydroxymyristoyl] N-acetylglucosamine deacetylase |
| STM0160 | yacL | 1 | 17 | hypothetical protein |
| STM0211 | yaeH | 13 | 17 | putative cytoplasmic protein |
| STM0311 | yafJ | 0 | 17 | putative glutamine amidotransferase |
| STM0327 |  | 6 | 17 | putative cytoplasmic protein |
| STM0440 | cyoD | 6 | 17 | cytochrome o ubiquinol oxidase subunit IV |
| STM0465 | ybaY | 11 | 17 | hypothetical protein |
| STM0733 | sdhD | 6 | 17 | succinate dehydrogenase cytochrome b556 small membrane subunit |
| STM0772 | gpmA | 12 | 17 | phosphoglyceromutase |
| STM0962 | ycaJ | 3 | 17 | hypothetical protein |
| STM0971 |  | 0 | 17 | putative cytoplasmic protein |
| STM1177 | flgE | 13 | 17 | flagellar hook protein |
| STM1444 | slyA | 2 | 17 | transcriptional regulator SlyA |
| STM1626 | trg | 1 | 17 | methyl-accepting chemotaxis protein III |
| STM1638 |  | 1 | 17 | putative SAM-dependent methyltransferase |
| STM1830 | manX | 12 | 17 | mannose-specific enzyme IIAB |
| STM1972 | fliI | 3 | 17 | flagellum-specific ATP synthase |
| STM2061 | sbmC | 8 | 17 | DNA gyrase inhibitor |
| STM2767 |  | 1 | 17 | putative DNA/RNA helicase |
| STM2796 | yqaE | 0 | 17 | putative transport protein |
| STM2800 |  | 1 | 17 | putative inner membrane protein |
| STM2865 | avrA | 2 | 17 | secreted effector protein |
| STM3056 | visC | 3 | 17 | hypothetical protein |
| STM3069 | pgk | 17 | 17 | phosphoglycerate kinase |
| STM3154 |  | 0 | 17 | putative ATP-dependent RNA helicase-like protein |
| STM3184 | yqiB | 3 | 17 | putative cytoplasmic protein |
| STM3188 | ygiC | 6 | 17 | putative glutathionylspermidine synthase |
| STM3228 | yqjC | 5 | 17 | putative periplasmic protein |
| STM3407 | fmt | 3 | 17 | methionyl-tRNA formyltransferase |
| STM3436 | rpsS | 8 | 17 | 30S ribosomal protein S19 |
| STM3440 | rplC | 3 | 17 | 50S ribosomal protein L3 |
| STM3715 | rfaZ | 0 | 17 | lipopolysaccharide core biosynthetic protein |
| STM3879 | yieN | 0 | 17 | putative regulatory protein |
| STM3885 | rbsK | 1 | 17 | ribokinase |
| STM4411 | ytfP | 2 | 17 | putative cytoplasmic protein |
| STM0068 | caiF | 0 | 16 | of cai/fix operon transcriptional regulator |
| STM0222 | cdsA | 4 | 16 | CDP-diglyceride synthase |
| STM0228 | lpxA | 1 | 16 | UDP-N-acetylglucosamine acyltransferase |
| STM0446 | bolA | 1 | 16 | putative regulatory protein |
| STM0448 | clpP | 2 | 16 | ATP-dependent Clp protease proteolytic subunit |
| STM1227 | pepT | 4 | 16 | peptidase T |
| STM1313 | celB | 0 | 16 | sugar-specific enzyme II |
| STM1328 |  | 2 | 16 | putative outer membrane protein |
| STM1389 | orf319 | 4 | 16 | putative inner membrane protein |
| STM1682 | tpx | 1 | 16 | thiol peroxidase |
| STM1839 |  | 1 | 16 | hypothetical protein |
| STM1915 | cheZ | 2 | 16 | chemotactic response protein |
| STM2060 | yeeA | 1 | 16 | putative inner membrane protein |
| STM2259 | napA | 23 | 16 | periplasmic nitrate reductase |
| STM2299 | yfbG | 9 | 16 | hypothetical protein |
| STM2300 |  | 3 | 16 | putative cytoplasmic protein |
| STM2316.S | nuoN | 2 | 16 | NADH dehydrogenase subunit N |
| STM2325 | nuoE | 5 | 16 | ATP synthase subunit E |
| STM2337 | ackA | 5 | 16 | acetate/propionate kinase |
| STM2390 | yfcZ | 4 | 16 | putative cytoplasmic protein |
| STM2652 | pssA | 2 | 16 | phosphatidylserine synthase |
| STM3068 | fba | 9 | 16 | fructose-bisphosphate aldolase |
| STM3320 | rpoN | 9 | 16 | DNA-directed RNA polymerase subunit N |
| STM3321 | yhbH | 7 | 16 | putative sigma N modulation factor |
| STM3728 | rpmB | 1 | 16 | 50S ribosomal protein L28 |
| STM3917 | rho | 2 | 16 | transcription termination factor Rho |
| STM4495 |  | 5 | 16 | putative type II restriction enzyme methylase subunit |
| STM4512 | iadA | 1 | 16 | isoaspartyl dipeptidase |
| STM4541 | mdoB | 3 | 16 | phosphoglycerol transferase I |
| STM0012 | dnaK | 8 | 15 | molecular chaperone DnaK |
| STM0126 | murD | 4 | 15 | UDP-N-acetylmuramoyl-L-alanyl-D-glutamate synthetase |
| STM0127 | ftsW | 3 | 15 | essential cell division gene |
| STM0666 | lnt | 4 | 15 | apolipoprotein N-acyltransferase |
| STM0667 | ybeX | 3 | 15 | putative transport protein |
| STM1071 | sulA | 3 | 15 | cell division inhibitor |
| STM1400 | sseC | 2 | 15 | translocation machinery component |
| STM1445 | slyB | 6 | 15 | putative outer membrane lipoprotein |
| STM1463 | add | 2 | 15 | adenosine deaminase |
| STM1480 | pntB | 1 | 15 | pyridine nucleotide transhydrogenase |
| STM1533 |  | 1 | 15 | putative hydrogenase |
| STM1641 | hrpA | 3 | 15 | ATP-dependent helicase |
| STM1651 | nifJ | 3 | 15 | putative pyruvate-flavodoxin oxidoreductase |
| STM1805 | fadR | 1 | 15 | fatty acid metabolism regulator |
| STM2084 | rfbM | 1 | 15 | mannose-1-phosphate guanylyltransferase |
| STM2089 | rfbJ | 1 | 15 | CDP-abequose synthase |
| STM2309 | menD | 4 | 15 | 2-oxoglutarate decarboxylase |
| STM2346 |  | 0 | 15 | putative NTP pyrophosphohydrolase |
| STM2472 | maeB | 5 | 15 | phosphate acetyltransferase |
| STM2646 | yfiD | 9 | 15 | putative formate acetyltransferase |
| STM2952 | eno | 5 | 15 | phosphopyruvate hydratase |
| STM3002 | lgt | 0 | 15 | prolipoprotein diacylglyceryl transferase |
| STM3054 | gcvH | 3 | 15 | glycine cleavage system protein H |
| STM3209 | rpsU | 2 | 15 | 30S ribosomal protein S21 |
| STM3402 | yrdC | 1 | 15 | putative dsRNA-binding protein |
| STM3416 | rpsD | 7 | 15 | 30S ribosomal protein S4 |
| STM3418 | rpsM | 4 | 15 | 30S ribosomal protein S13 |
| STM3446 | fusA | 19 | 15 | elongation factor EF-2 |
| STM3537 | glgX | 3 | 15 | glycogen debranching enzyme |
| STM3586.S | yhiH | 5 | 15 | putative ABC-type multidrug transport system ATPase component |
| STM3864 | atpC | 13 | 15 | ATP synthase subunit epsilon |
| STM3958 | recQ | 0 | 15 | ATP-dependent DNA helicase |
| STM3999 | polA | 4 | 15 | DNA polymerase I |
| STM4073 | ydeW | 1 | 15 | putative transcriptional repressor |
| STM4241 | zur | 0 | 15 | transcriptional repressor |
| STM4290 | proP | 2 | 15 | low-affinity proline transporter |
| STM4297 | melR | 1 | 15 | melibiose operon regulator |
| STM4359 | mutL | 4 | 15 | DNA mismatch repair protein |
| STM0064 | dapB | 1 | 14 | dihydrodipicolinate reductase |
| STM0365 | yahN | 0 | 14 | putative transport protein |
| STM0417 | ribH | 0 | 14 | riboflavin synthase subunit beta |
| STM0665 | gltI | 5 | 14 | glutamate/aspartate transporter |
| STM0694 | fldA | 0 | 14 | flavodoxin |
| STM0743 | ybgE | 3 | 14 | putative inner membrane lipoprotein |
| STM0963 | serS | 5 | 14 | seryl-tRNA synthetase |
| STM1234.S | trmU | 4 | 14 | tRNA (5-methylaminomethyl-2-thiouridylate)-methyltransferase |
| STM1239 |  | 1 | 14 | putative cytoplasmic protein |
| STM1284 | yeaH | 4 | 14 | hypothetical protein |
| STM1409 | ssaJ | 0 | 14 | needle complex inner membrane lipoprotein |
| STM1589 | yncB | 9 | 14 | putative NADP-dependent oxidoreductase |
| STM1712 | acnA | 8 | 14 | aconitate hydratase |
| STM1731 |  | 3 | 14 | putative catalase |
| STM1845 | prc | 2 | 14 | carboxy-terminal protease |
| STM1846 | proQ | 0 | 14 | putative solute/DNA competence effector |
| STM2033 | cbiC | 1 | 14 | precorrin-8X methylmutase |
| STM2090 | rfbH | 6 | 14 | CDP-6-deoxy-D-xylo-4-hexulose-3-dehydrase |
| STM2297 | yfbE | 2 | 14 | 4-amino-4-deoxy-L-arabinose lipopolysaccharide-modifying enzyme |
| STM2298 | pmrF | 3 | 14 | putative glycosyl transferase |
| STM2320 | nuoJ | 3 | 14 | NADH dehydrogenase subunit J |
| STM2489 | dapA | 2 | 14 | dihydrodipicolinate synthase |
| STM2674 | trmD | 3 | 14 | tRNA (guanine-N(1)-)-methyltransferase |
| STM2829 | recA | 3 | 14 | recombinase A |
| STM2866 | sprB | 2 | 14 | transcriptional regulator |
| STM2877 | iagB | 4 | 14 | invasion protein precursor |
| STM3058 | pepP | 4 | 14 | proline aminopeptidase P II |
| STM3061 | ygfA | 0 | 14 | putative ligase |
| STM3149 | hybA | 0 | 14 | putative hydrogenase-2 component |
| STM3223 | ygjR | 4 | 14 | putative dehydrogenase |
| STM3225 | ygjU | 3 | 14 | putative dicarboxylate permease |
| STM3342 | sspA | 2 | 14 | stringent starvation protein A |
| STM3369 | yhdP | 2 | 14 | putative protease |
| STM3534 | glgP | 4 | 14 | glycogen phosphorylase |
| STM3919 | wzzE | 0 | 14 | enterobacterial common antigen chain length regulator |
| STM3939 | cyaA | 3 | 14 | adenylate cyclase |
| STM3947 | dapF | 2 | 14 | diaminopimelate epimerase |
| STM4089 | menG | 5 | 14 | ribonuclease activity regulator protein RraA |
| STM4409 | ytfM | 2 | 14 | putative outer membrane protein |
| STM4517 | yjiO | 0 | 14 | putative transport protein |
| STM4586 | rob | 1 | 14 | transcriptional regulator |
| STM0123 | murE | 5 | 13 | UDP-N-acetylmuramoylalanyl-D-glutamate--2,6-diaminopimelate ligase |
| STM0212 |  | 1 | 13 | putative inner membrane protein |
| STM0537 | cysS | 2 | 13 | cysteinyl-tRNA synthetase |
| STM0543 | fimA | 1 | 13 | fimbrin |
| STM0791 | hutH | 7 | 13 | histidine ammonia-lyase |
| STM0865 | ybjG | 1 | 13 | putative permease |
| STM0940 | ybjX | 1 | 13 | VirK-like protein |
| STM0944 | clpS | 2 | 13 | ATP-dependent Clp protease adaptor protein ClpS |
| STM0961 | lolA | 4 | 13 | outer-membrane lipoprotein carrier protein precursor |
| STM1112 | cbpA | 0 | 13 | DNA-binding protein |
| STM1221 | cobB | 1 | 13 | NAD-dependent deacetylase |
| STM1488 | mlc | 4 | 13 | pts operon transcriptional repressor |
| STM1679 | mppA | 4 | 13 | periplasmic murein tripeptide transport protein |
| STM1777 | hemA | 3 | 13 | glutamyl-tRNA reductase |
| STM1848 | yebS | 1 | 13 | putative inner membrane protein |
| STM1881 | yebF | 4 | 13 | putative periplasmic protein |
| STM1886 | zwf | 3 | 13 | glucose-6-phosphate 1-dehydrogenase |
| STM1935 | ftn | 3 | 13 | cytoplasmic ferritin |
| STM1946 | uvrC | 5 | 13 | excinuclease ABC subunit C |
| STM1979 | fliP | 3 | 13 | flagellar biosynthesis protein |
| STM2080 | udg | 1 | 13 | UDP-glucose/GDP-mannose dehydrogenase |
| STM2086 | rfbU | 2 | 13 | mannosyl transferase |
| STM2093 | rfbI | 6 | 13 | CDP-6-deoxy-delta-3,4-glucoseen reductase |
| STM2215 | rtn | 1 | 13 | hypothetical protein |
| STM2226 | yejK | 0 | 13 | nucleoid-associated protein NdpA |
| STM2317 | nuoM | 5 | 13 | NADH dehydrogenase subunit M |
| STM2321 | nuoI | 3 | 13 | NADH dehydrogenase subunit I |
| STM2324 | nuoF | 8 | 13 | NADH dehydrogenase I chain F |
| STM2336 |  | 0 | 13 | hypothetical protein |
| STM2638 | rseB | 0 | 13 | periplasmic negative regulator of sigmaE |
| STM2662 | rluD | 3 | 13 | ribosomal large subunit pseudouridine synthase D |
| STM2677 | ffh | 5 | 13 | 4.5S-RNP protein |
| STM2887 | spaS | 2 | 13 | type III secretion protein |
| STM3157 | yghA | 6 | 13 | oxidoreductase |
| STM3226 | yqjA | 1 | 13 | hypothetical protein |
| STM3296 | hflB | 9 | 13 | ATP-dependent zinc-metallo protease |
| STM3368 | tldD | 4 | 13 | microcin B17-processing protein |
| STM3410 | mscL | 4 | 13 | large-conductance mechanosensitive channel |
| STM3536 | glgC | 7 | 13 | glucose-1-phosphate adenylyltransferase |
| STM3569 | ftsX | 2 | 13 | putative cell division protein |
| STM3704 | pmgI | 5 | 13 | phosphoglyceromutase |
| STM3719 | rfaB | 0 | 13 | lipopolysaccharide-1,6-D-galactosyltransferase |
| STM3871 | atpB | 6 | 13 | ATP synthase subunit A |
| STM3973 | tatA | 2 | 13 | twin argininte translocase protein A |
| STM4275 | acs | 1 | 13 | acetyl-coenzyme A synthetase |
| STM4367 | yjeB | 3 | 13 | putative negative regulator |
| STM4404 | cysQ | 2 | 13 | sulfite biosynthetic protein |
| STM4580.S | nadR | 0 | 13 | nicotinamide-nucleotide adenylyltransferase |
| STM0131 | ftsQ | 2 | 12 | cell division protein |
| STM0216 | rpsB | 5 | 12 | 30S ribosomal protein S2 |
| STM0217 | tsf | 6 | 12 | elongation factor Ts |
| STM0408 | secF | 1 | 12 | protein export protein SecF |
| STM0442 | cyoB | 11 | 12 | cytochrome o ubiquinol oxidase subunit I |
| STM0450 | lon | 5 | 12 | ATP-dependent protease Lon |
| STM0542 | folD | 0 | 12 | 5,10-methylene-tetrahydrofolate dehydrogenase/5,10-methylene-tetrahydrofolate cyclohydrolase |
| STM0669 | phoL | 3 | 12 | putative phosphate starvation-inducible protein |
| STM0683 | nagA | 5 | 12 | N-acetylglucosamine-6-phosphate deacetylase |
| STM0802 | moaA | 2 | 12 | molybdenum cofactor biosynthesis protein A |
| STM0807 | ybhL | 3 | 12 | putative permease |
| STM0888 | artM | 0 | 12 | arginine transport system component |
| STM0934 | ltaA | 1 | 12 | L-allo-threonine aldolase |
| STM1119 | wraB | 8 | 12 | TrpR binding protein WrbA |
| STM1246 | pagC | 14 | 12 | virulence membrane protein PAGC precursor |
| STM1486 | ynfM | 0 | 12 | putative transport protein |
| STM1511 | ydfG | 0 | 12 | putative oxidoreductase |
| STM1558 |  | 2 | 12 | putative glycosyl hydrolase |
| STM1582 | nhoA | 0 | 12 | putative arylamine N-acetyltransferase |
| STM1710 | pgpB | 0 | 12 | phosphatidylglycerophosphate phosphatase B |
| STM1743 | oppD | 3 | 12 | oligopeptide transporter ATP-binding component |
| STM1752 | galU | 1 | 12 | glucose-1-phosphate uridylyltransferase |
| STM1807 | dsbB | 0 | 12 | disulfide bond formation protein B |
| STM1976 | fliM | 9 | 12 | flagellar motor switch protein |
| STM2067 | sbcB | 2 | 12 | exonuclease I |
| STM2217 | yejB | 1 | 12 | putative ABC-type dipeptide/oligopeptide/nickel transport system permease |
| STM2246 | narP | 0 | 12 | response regulator |
| STM2378 | fabB | 10 | 12 | 3-oxoacyl-(acyl carrier protein) synthase |
| STM2388 | yfcX | 1 | 12 | putative dehydrogenase |
| STM2520 | yfgL | 3 | 12 | putative serine/threonine protein kinase |
| STM2688 | smpB | 1 | 12 | SsrA-binding protein |
| STM2781 | virK | 1 | 12 | virulence protein |
| STM2814 | emrA | 0 | 12 | multidrug resistance secretion protein |
| STM2950 |  | 2 | 12 | putative metal-dependent hydrolase |
| STM3040 | lysS | 5 | 12 | lysyl-tRNA synthetase |
| STM3107 | yggN | 1 | 12 | putative periplasmic protein |
| STM3153 | yqhA | 3 | 12 | hypothetical protein |
| STM3189 | ygiD | 0 | 12 | putative cytoplasmic protein |
| STM3201 | glnE | 2 | 12 | adenylyl transferase for glutamine synthetase |
| STM3297 | ftsJ | 2 | 12 | 23S rRNA methyltransferase |
| STM3441 | rpsJ | 2 | 12 | 30S ribosomal protein S10 |
| STM3453 | fkpA | 3 | 12 | FKBP-type peptidyl-prolyl cis-trans isomerase |
| STM3589 | pitA | 0 | 12 | low-affinity phosphate transporter |
| STM3602 |  | 1 | 12 | putative regulatory protein |
| STM3758 | fidL | 0 | 12 | putative inner membrane protein |
| STM3872 | atpI | 2 | 12 | ATP synthase subunit I |
| STM3970 | ubiE | 0 | 12 | ubiquinone/menaquinone biosynthesis methyltransferase |
| STM4127 | yijC | 1 | 12 | putative transcriptional repressor |
| STM4249 | aphA | 3 | 12 | non-specific acid phosphatase/phosphotransferase |
| STM4390 |  | 1 | 12 | putative cytoplasmic protein |
| STM4392 | priB | 1 | 12 | primosomal replication protein N |
| STM0046 | ileS | 4 | 11 | isoleucyl-tRNA synthetase |
| STM0047 | lspA | 3 | 11 | signal peptidase II |
| STM0121 | ftsL | 0 | 11 | cell division protein |
| STM0221 | uppS | 0 | 11 | undecaprenyl pyrophosphate synthetase |
| STM0475 | acrB | 12 | 11 | acridine efflux pump |
| STM0476 | acrA | 2 | 11 | acridine efflux pump |
| STM0484 | dnaX | 2 | 11 | DNA polymerase III subunits gamma and tau |
| STM0648 | leuS | 7 | 11 | leucyl-tRNA synthetase |
| STM0685 | nagE | 2 | 11 | N-acetylglucosamine-specific enzyme IIABC |
| STM0781 | modA | 4 | 11 | molybdate transporter |
| STM0814 | ybhQ | 5 | 11 | putative inner membrane protein |
| STM0819 | ybiH | 1 | 11 | putative transcriptional repressor |
| STM0870 |  | 0 | 11 | hypothetical protein |
| STM0941 | ybjY | 1 | 11 | hypothetical protein |
| STM0977 | serC | 3 | 11 | phosphoserine aminotransferase |
| STM1147 |  | 0 | 11 | hypothetical protein |
| STM1167 | rimJ | 0 | 11 | acetylatase |
| STM1185 | rne | 0 | 11 | RNase E |
| STM1195 | fabG | 4 | 11 | 3-ketoacyl-(acyl-carrier-protein) reductase |
| STM1300 |  | 1 | 11 | putative periplasmic protein |
| STM1415 | ssaN | 0 | 11 | type III secretion system ATPase |
| STM1468 | fumA | 5 | 11 | fumarase A |
| STM1594 | srfB | 5 | 11 | putative virulence protein |
| STM1744 | oppC | 3 | 11 | oligopeptide transport protein |
| STM1847 | yebR | 0 | 11 | putative nucleotide-binding protein |
| STM1900 | ntpA | 0 | 11 | dATP pyrophosphohydrolase |
| STM1939 |  | 0 | 11 | putative glucose-6-phosphate dehydrogenase |
| STM2028 | cbiG | 2 | 11 | cobalamin biosynthesis protein CbiG |
| STM2066 | sopA | 0 | 11 | secreted effector protein |
| STM2165 | yehZ | 0 | 11 | putative transport protein |
| STM2186 |  | 4 | 11 | putative NADPH-dependent glutamate synthase beta chain |
| STM2362 | purF | 6 | 11 | amidophosphoribosyltransferase |
| STM2446 |  | 0 | 11 | putative iron-dependent peroxidase |
| STM2494 |  | 3 | 11 | hypothetical protein |
| STM2536 | pepB | 1 | 11 | aminopeptidase B |
| STM2819 | yqaA | 0 | 11 | putative inner membrane protein |
| STM3132 |  | 4 | 11 | putative xylanase/chitin deacetylase |
| STM3133 |  | 2 | 11 | putative amidohydrolase |
| STM3144 | hypA | 0 | 11 | hydrogenase nickel incorporation protein |
| STM3207 | ygiH | 2 | 11 | hypothetical protein |
| STM3230 | yqjE | 6 | 11 | putative inner membrane protein |
| STM3362 |  | 0 | 11 | putative periplasmic protein |
| STM3421 | rplO | 11 | 11 | 50S ribosomal protein L15 |
| STM3428 | rplE | 4 | 11 | 50S ribosomal protein L5 |
| STM3465 | yhfA | 3 | 11 | putative inner membrane protein |
| STM3467 | yhfK | 1 | 11 | putative inner membrane protein |
| STM3481 | trpS | 3 | 11 | tryptophanyl-tRNA synthetase |
| STM3568 | rpoH | 7 | 11 | RNA polymerase sigma factor |
| STM3590 | uspB | 0 | 11 | universal stress protein UspB |
| STM3614 | dctA | 0 | 11 | C4-dicarboxylate transport protein |
| STM3617 |  | 1 | 11 | endo-1,4-D-glucanase |
| STM3718 | rfaI | 4 | 11 | lipopolysaccharide-alpha-1,3-D-galactosyltransferase |
| STM3773 |  | 2 | 11 | putative transcriptional regulator |
| STM3797 | ivbL | 3 | 11 | ilvB operon leader peptide |
| STM3915 | trxA | 5 | 11 | thioredoxin |
| STM3938 | hemC | 4 | 11 | porphobilinogen deaminase |
| STM3961 | pldB | 2 | 11 | lysophospholipase L2 |
| STM3975 | tatC | 0 | 11 | Sec-independent protein secretion pathway component |
| STM3978 | yigC | 1 | 11 | putative oxidoreductase |
| STM4062 | pfkA | 4 | 11 | 6-phosphofructokinase |
| STM4221 | pgi | 3 | 11 | glucose-6-phosphate isomerase |
| STM4239 |  | 5 | 11 | putative cytoplasmic protein |
| STM4334 | efp | 1 | 11 | elongation factor P |
| STM4378 | yjfN | 8 | 11 | putative inner membrane protein |
| STM4379 | yjfO | 14 | 11 | putative lipoprotein |
| STM4416 | mpl | 0 | 11 | UDP-N-acetylmuramate/L-alanyl-gamma-D-glutamyl-meso-diaminopimelate ligase |
| STM4470 | yjgD | 1 | 11 | putative cytoplasmic protein |
| STM4514.S | yjiH | 0 | 11 | putative inner membrane protein |
| STM4533 | tsr | 3 | 11 | methyl-accepting chemotaxis protein I |
| STM4563 | yjjU | 0 | 11 | putative phosphoesterase |
| STM0003 | thrB | 2 | 10 | homoserine kinase |
| STM0159 |  | 1 | 10 | putative restriction endonuclease |
| STM0318 | yafA | 3 | 10 | hypothetical protein |
| STM0376 | sbmA | 0 | 10 | putative ABC transporter membrane protein |
| STM0407 | secD | 1 | 10 | protein export protein SecD |
| STM0494 | ushA | 3 | 10 | UDP-sugar hydrolase/5'-nucleotidase |
| STM0652 |  | 6 | 10 | putative sigma-54 dependent transcriptional regulator |
| STM0872 | grxA | 0 | 10 | glutaredoxin 1 |
| STM0890 | artI | 1 | 10 | arginine transport system |
| STM0999 | ompF | 3 | 10 | outer membrane protein F precursor |
| STM1018 |  | 0 | 10 | hypothetical protein |
| STM1176 | flgD | 6 | 10 | flagellar basal body rod modification protein |
| STM1178 | flgF | 4 | 10 | cell-proximal portion of basal-body rod |
| STM1269 |  | 0 | 10 | chorismate mutase |
| STM1270 | yeaS | 0 | 10 | putative transport protein |
| STM1274 | yeaQ | 1 | 10 | putative inner membrane protein |
| STM1297 | selD | 1 | 10 | selenophosphate synthetase |
| STM1312 | celA | 0 | 10 | sugar-specific enzyme IIB |
| STM1345 | ydiU | 2 | 10 | hypothetical protein |
| STM1391 | ssrB | 3 | 10 | transcriptional activator |
| STM1399 | sscA | 0 | 10 | secretion system chaparone |
| STM1410 |  | 0 | 10 | putative cytoplasmic protein |
| STM1433 | ydhD | 3 | 10 | putative glutaredoxin protein |
| STM1536 |  | 0 | 10 | putative hydrogenase maturation protease |
| STM1685 | ycjX | 0 | 10 | putative ATPase |
| STM1714 | topA | 2 | 10 | DNA topoisomerase I |
| STM1815 | minD | 5 | 10 | cell division inhibitor protein |
| STM1831 | manY | 8 | 10 | mannose-specific enzyme IIC |
| STM1832 | manZ | 5 | 10 | mannose-specific enzyme IID |
| STM1907 | cutC | 0 | 10 | copper homeostasis protein |
| STM1961 | fliS | 5 | 10 | flagellar protein FliS |
| STM2026 | cbiJ | 2 | 10 | precorrin-6x reductase |
| STM2032 | cbiD | 3 | 10 | cobalt-precorrin-6A synthase |
| STM2034 | cobD | 1 | 10 | cobalamin biosynthesis protein |
| STM2036 | pocR | 2 | 10 | transcriptional regulator |
| STM2039 | pudB | 7 | 10 | polyhedral body protein |
| STM2087 | rfbV | 1 | 10 | abequosyltransferase |
| STM2154 | mrp | 0 | 10 | putative ATP-binding protein |
| STM2164 | yehY | 2 | 10 | putative ABC-type proline/glycine betaine transport system permease component |
| STM2189 | mglA | 0 | 10 | methyl-galactoside transport protein |
| STM2190 | mglB | 1 | 10 | galactose transport protein |
| STM2201 | yeiE | 1 | 10 | putative transcriptional regulator |
| STM2306 | menC | 1 | 10 | O-succinylbenzoate synthase |
| STM2330 | lrhA | 0 | 10 | NADH dehydrogenase transcriptional repressor |
| STM2361 |  | 3 | 10 | putative regulatory protein |
| STM2483 | dapE | 2 | 10 | succinyl-diaminopimelate desuccinylase |
| STM2523 | gcpE | 1 | 10 | 4-hydroxy-3-methylbut-2-en-1-yl diphosphate synthase |
| STM2524 | yfgA | 9 | 10 | hypothetical protein |
| STM2557 | cadC | 0 | 10 | transcriptional activator |
| STM2679 | yfjD | 1 | 10 | hypothetical protein |
| STM2858 | hypE | 2 | 10 | putative hydrogenase formation protein |
| STM3043 | dsbC | 0 | 10 | protein disulfide isomerase II |
| STM3122 |  | 1 | 10 | putative arylsulfatase |
| STM3195 | ribB | 3 | 10 | 3,4-dihydroxy-2-butanone 4-phosphate synthase |
| STM3266 | yraO | 1 | 10 | putative phosphoheptose isomerase |
| STM3338 | nanT | 0 | 10 | putative sialic acid transporter |
| STM3341 | sspB | 1 | 10 | stringent starvation protein B |
| STM3347 | yhcB | 0 | 10 | putative periplasmic protein |
| STM3363 | yhcO | 1 | 10 | putative cytoplasmic protein |
| STM3423 | rpsE | 6 | 10 | 30S ribosomal protein S5 |
| STM3439 | rplD | 1 | 10 | 50S ribosomal protein L4 |
| STM3570 | ftsE | 0 | 10 | putative cell division ATPase |
| STM3594 | prlC | 2 | 10 | oligopeptidase A |
| STM3624 | yhjU | 4 | 10 | putative inner membrane protein |
| STM3703 | yibN | 3 | 10 | putative rhodanese-like sulfurtransferase |
| STM3845 |  | 0 | 10 | putative inner membrane protein |
| STM3926 | wzxE | 0 | 10 | O-antigen translocase |
| STM4091 | hslU | 2 | 10 | ATP-dependent protease ATP-binding subunit |
| STM4093 | ftsN | 0 | 10 | essential cell division protein |
| STM4147 | secE | 0 | 10 | translocase |
| STM4314 |  | 0 | 10 | putative regulatory protein |
| STM4403 | cpdB | 1 | 10 | 2',3'-cyclic nucleotide 2'-phosphodiesterase/3'-nucleotidase bifunctional periplasmic precursor protein |
| STM4438 | pmbA | 0 | 10 | putative antibiotic maturation protein |
| STM4503 |  | 3 | 10 | putative inner membrane protein |
| STM4513 | yjiG | 0 | 10 | putative permease |
| STM4524 | hsdS | 0 | 10 | type I restriction enzyme specificity protein |

^a^Gene names according to ColiBase [3]

^b^Based on 145,873 sequences

^c^Based on 122,326 sequences

^d^Product according to KEGG ([http://www.genome.jp/kegg/](http://www.pubmedcentral.nih.gov/redirect3.cgi?&&reftype=extlink&artid=1810395&iid=129405&jid=379&FROM=Article|Body&TO=External|Link|URI&article-id=1810395&journal-id=379&rendering-type=normal&&http://www.genome.jp/kegg/); [4])
