# Supplementary material for: Deep Sequencing Analysis of Small Noncoding RNA and mRNA Targets of the Global Post-Transcriptional Regulator, Hfq
Source: PLoS Genet. 2008 Aug 22;4(8):e1000163. doi: 10.1371/journal.pgen.1000163 (PMC2515195; doi:10.1371/journal.pgen.1000163)
Supplement: Table S5 — Genes that were significantly enriched in coIP-on-Chip and were identified by pyrosequencing. (0.32 MB DOC) [file pgen.1000163.s010.doc]

**Table S5: Genes that were significantly enriched in coIP-on-Chip and were identified by pyrosequencing**

| **Gene name^a^** | **Enrichment**  **overr wt^b^** | **454** |
| --- | --- | --- |
| STM2506 | 21.12 |  |
| ybfM | 19.29 | X |
| STM1747 | 17.10 |  |
| aphA | 15.51 | X |
| ytfK | 14.23 | X |
| cpxP | 11.67 | X |
| cof | 10.15 | X |
| yqjA | 10.15 | X |
| STM1841 | 8.92 | X |
| oppA | 8.66 | X |
| STM2880 | 8.53 |  |
| STM1939 | 8.40 | X |
| ygdQ | 8.22 | X |
| cutC | 8.09 | X |
| glpF | 7.95 | X |
| yceB | 7.87 | X |
| cspE | 7.50 | X |
| sicP | 7.47 | X |
| yihO | 7.42 |  |
| dppA | 7.42 | X |
| STM0159 | 7.37 | X |
| STM2281 | 7.35 |  |
| hfq | 7.31 | X |
| yceP | 7.30 | X |
| truA | 7.05 | X |
| gltI | 7.03 | X |
| rfbP | 7.03 | X |
| yejG | 7.02 |  |
| yafJ | 7.01 | X |
| orgA | 6.89 | X |
| miaA | 6.89 | X |
| yidF | 6.86 | X |
| zwf | 6.72 | X |
| acrR | 6.71 |  |
| rfbX | 6.62 | X |
| nuoA | 6.57 | X |
| STM1128 | 6.51 |  |
| rfbU | 6.41 | X |
| ychK | 6.38 | X |
| lrhA | 6.26 | X |
| yeaJ | 6.21 | X |
| STM2507 | 6.16 |  |
| rfaB | 6.11 | X |
| yabI | 6.08 |  |
| pykA | 6.07 | X |
| hilA | 6.04 | X |
| ssaS | 6.00 |  |
| ssaT | 6.00 |  |
| ytfL | 5.92 |  |
| STM3845 | 5.87 | X |
| pepN | 5.85 | X |
| fmt | 5.83 | X |
| ucpA | 5.83 | X |
| yqiB | 5.79 | X |
| pitA | 5.71 | X |
| icc | 5.68 | X |
| rpoS | 5.64 | X |
| STM0082 | 5.62 |  |
| ybaP | 5.62 |  |
| hilC | 5.62 | X |
| hsdS | 5.59 | X |
| ddlA | 5.56 |  |
| STM0571 | 5.49 |  |
| ycbW | 5.46 |  |
| celA | 5.43 | X |
| pocR | 5.39 | X |
| rfbJ | 5.37 | X |
| STM1530 | 5.35 |  |
| maa | 5.33 | X |
| sucD | 5.31 | X |
| yafK | 5.27 |  |
| ygjU | 5.24 | X |
| STM1093 | 5.22 |  |
| STM4312 | 5.19 |  |
| yeeI | 5.13 | X |
| STM2901 | 5.09 |  |
| STM4313 | 5.07 |  |
| marA | 5.07 |  |
| STM2870 | 5.06 |  |
| narP | 5.06 | X |
| STM2742 | 5.06 |  |
| sptP | 5.00 | X |
| yrdC | 4.96 | X |
| dedA | 4.95 |  |
| STM2747 | 4.95 |  |
| yjbN | 4.94 | X |
| STM2690 | 4.94 |  |
| nlpC | 4.92 |  |
| ylbA | 4.90 |  |
| STM2705 | 4.87 |  |
| STM3461 | 4.82 |  |
| marR | 4.80 |  |
| ygcA | 4.80 |  |
| invH | 4.80 |  |
| rfaZ | 4.79 | X |
| solA | 4.79 |  |
| yebK | 4.78 | X |
| sanA | 4.76 | X |
| sdhC | 4.75 | X |
| glmS | 4.74 |  |
| imp | 4.72 | X |
| iacP | 4.72 | X |
| STM2986 | 4.71 |  |
| argR | 4.70 |  |
| ytfJ | 4.70 |  |
| nirD | 4.70 |  |
| relA | 4.70 | X |
| STM4257 | 4.69 | X |
| mglA | 4.68 | X |
| phnA | 4.66 |  |
| mltC | 4.65 |  |
| rfaI | 4.63 | X |
| rfaC | 4.63 |  |
| STM3846 | 4.62 |  |
| yidG | 4.60 |  |
| rtsA | 4.58 |  |
| ycfJ | 4.57 |  |
| STM0497 | 4.56 |  |
| glnH | 4.55 |  |
| rbsB | 4.54 | X |
| cspA | 4.49 | X |
| rfaJ | 4.49 |  |
| yecA | 4.47 | X |
| mglC | 4.45 |  |
| STM4493 | 4.42 |  |
| STM4497 | 4.41 |  |
| yhjW | 4.41 |  |
| thyA | 4.39 |  |
| hilD | 4.39 | X |
| ompX | 4.39 | X |
| STM1018 | 4.38 | X |
| hha | 4.36 |  |
| ptsG | 4.33 | X |
| ytfM | 4.32 | X |
| STM1839 | 4.32 | X |
| ais | 4.30 | X |
| STM4316 | 4.30 |  |
| oppF | 4.29 | X |
| celG | 4.27 | X |
| ydeD | 4.26 |  |
| yiaG | 4.25 |  |
| flgL | 4.25 | X |
| yejK | 4.24 | X |
| STM3528 | 4.24 |  |
| STM2280 | 4.22 | X |
| rfbV | 4.20 | X |
| STM2238 | 4.19 |  |
| STM4261 | 4.19 | X |
| ydiV | 4.18 |  |
| STM1554 | 4.17 |  |
| ychJ | 4.16 |  |
| STM1023 | 4.12 |  |
| STM4260 | 4.11 | X |
| STM4310 | 4.10 |  |
| STM2530 | 4.10 | X |
| ubiB | 4.08 |  |
| STM2746 | 4.07 |  |
| foxA | 4.06 |  |
| nlpD | 4.06 | X |
| adiY | 4.06 |  |
| STM1629 | 4.05 |  |
| STM1656 | 4.02 |  |
| STM2767 | 4.02 | X |
| polA | 4.02 | X |
| kdpE | 4.01 |  |
| aefA | 4.01 |  |
| STM2868 | 4.00 |  |
| nirB | 3.99 |  |
| def | 3.97 |  |
| STM2610 | 3.97 |  |
| flhD | 3.94 | X |
| malM | 3.94 | X |
| rfaK | 3.94 |  |
| slt | 3.93 | X |
| STM3251 | 3.93 |  |
| oafA | 3.92 |  |
| STM4258 | 3.92 | X |
| hybG | 3.91 |  |
| baeR | 3.90 |  |
| yidQ | 3.90 |  |
| yeiU | 3.88 |  |
| glpK | 3.88 | X |
| araC | 3.88 |  |
| cobB | 3.87 | X |
| sdhD | 3.87 | X |
| STM2314 | 3.86 | X |
| gnd | 3.86 | X |
| STM3773 | 3.86 | X |
| lnt | 3.85 | X |
| acrD | 3.84 |  |
| STM1874 | 3.82 |  |
| uvrY | 3.81 | X |
| ssaQ | 3.79 |  |
| ytgA | 3.78 |  |
| crp | 3.77 | X |
| STM2135 | 3.76 |  |
| STM1785 | 3.76 |  |
| yihG | 3.76 |  |
| STM2329 | 3.74 |  |
| tdh | 3.73 | X |
| rfaQ | 3.72 |  |
| STM1239 | 3.72 | X |
| spaO | 3.72 | X |
| rbsK | 3.70 | X |
| marB | 3.70 |  |
| invG | 3.69 | X |
| sdaC | 3.69 |  |
| aroE | 3.67 |  |
| sucC | 3.66 | X |
| rfc | 3.66 |  |
| rfbI | 3.65 | X |
| avrA | 3.64 | X |
| ymbA | 3.63 |  |
| STM4534 | 3.62 |  |
| STM2225 | 3.62 |  |
| dsbB | 3.61 | X |
| STM3362 | 3.61 | X |
| ybeX | 3.61 | X |
| STM4495 | 3.61 | X |
| alkB | 3.61 |  |
| STM1328 | 3.59 | X |
| STM1014 | 3.59 |  |
| lgt | 3.57 | X |
| nuoB | 3.57 | X |
| smpB | 3.54 | X |
| STM1130 | 3.54 |  |
| apaG | 3.53 |  |
| apaH | 3.53 | X |
| ybjE | 3.53 |  |
| dlhH | 3.53 |  |
| ttk | 3.52 |  |
| stdA | 3.51 |  |
| invF | 3.51 | X |
| STM0053 | 3.50 |  |
| yahN | 3.50 | X |
| STM2950 | 3.50 | X |
| STM0341 | 3.50 |  |
| sbmA | 3.50 | X |
| clpB | 3.49 | X |
| STM3155 | 3.49 |  |
| rfbM | 3.48 | X |
| yibR | 3.48 |  |
| hnr | 3.48 |  |
| STM4494 | 3.47 |  |
| STM1254 | 3.46 |  |
| yfeA | 3.46 |  |
| STM2186 | 3.45 | X |
| STM3651 | 3.44 |  |
| invC | 3.44 | X |
| STM3533 | 3.44 |  |
| hpaR | 3.43 |  |
| ydeZ | 3.43 | X |
| STM0307 | 3.43 |  |
| ygaC | 3.42 |  |
| yfaZ | 3.42 |  |
| orf245 | 3.42 |  |
| STM2754 | 3.42 |  |
| rfaL | 3.41 |  |
| ftn | 3.40 | X |
| pldB | 3.39 | X |
| hupB | 3.38 | X |
| yajD | 3.38 |  |
| STM3291 | 3.38 |  |
| dacC | 3.38 | X |
| STM4597 | 3.38 |  |
| yobA | 3.37 | X |
| yffH | 3.36 |  |
| ybjT | 3.36 |  |
| lpxO | 3.35 |  |
| invA | 3.35 | X |
| gltJ | 3.34 |  |
| mdoC | 3.34 |  |
| STM1673 | 3.34 |  |
| fepE | 3.34 |  |
| STM2766 | 3.34 |  |
| sfbA | 3.34 |  |
| STM4420 | 3.34 |  |
| mukB | 3.33 | X |
| STM2449 | 3.33 |  |
| yfeZ | 3.33 |  |
| yhfK | 3.32 | X |
| gltB | 3.32 |  |
| msbB | 3.31 |  |
| yecH | 3.31 |  |
| STM3084 | 3.31 |  |
| STM4308 | 3.31 |  |
| ubiX | 3.30 |  |
| flk | 3.30 |  |
| dfp | 3.30 |  |
| hflX | 3.30 | X |
| glnA | 3.29 |  |
| ydiJ | 3.29 |  |
| ygiH | 3.29 | X |
| lasT | 3.29 |  |
| STM1041 | 3.28 |  |
| STM0344 | 3.27 |  |
| yjgG | 3.27 |  |
| STM3906 | 3.26 |  |
| STM3907 | 3.26 |  |
| lrp | 3.26 | X |
| yciT | 3.25 |  |
| yeaL | 3.25 |  |
| fadD | 3.24 |  |
| ydeW | 3.23 | X |
| STM3698 | 3.23 |  |
| proP | 3.23 | X |
| yigZ | 3.22 |  |
| yicL | 3.22 |  |
| STM2011 | 3.22 |  |
| ybfE | 3.22 |  |
| rfbK | 3.21 | X |
| hemF | 3.21 |  |
| flhC | 3.20 | X |
| STM1532 | 3.19 |  |
| perM | 3.19 |  |
| yfgB | 3.19 |  |
| rfbC | 3.19 |  |
| ytfN | 3.18 | X |
| orfX | 3.18 |  |
| aroM | 3.17 |  |
| yabJ | 3.17 |  |
| yggN | 3.17 | X |
| proQ | 3.15 | X |
| STM3516 | 3.15 |  |
| yadQ | 3.14 |  |
| glgB | 3.14 | X |
| sopE2 | 3.12 | X |
| STM3138 | 3.12 | X |
| pagP | 3.12 |  |
| STM1697 | 3.11 |  |
| sipD | 3.11 | X |
| pqiA | 3.11 |  |
| sinR | 3.11 |  |
| STM0672 | 3.11 |  |
| yeaS | 3.11 | X |
| dapB | 3.10 | X |
| STM3785 | 3.09 |  |
| rplL | 3.09 | X |
| STM0870 | 3.09 | X |
| STM1550 | 3.08 |  |
| nhaA | 3.08 | X |
| STM0652 | 3.07 | X |
| ushA | 3.07 | X |
| STM3517 | 3.07 |  |
| ydgT | 3.06 |  |
| hscA | 3.06 |  |
| hscB | 3.06 |  |
| spaP | 3.06 | X |
| yjcB | 3.06 |  |
| STM2377 | 3.05 |  |
| yeaA | 3.05 | X |
| STM0835 | 3.05 |  |
| yfeL | 3.05 |  |
| araJ | 3.05 |  |
| cysS | 3.04 | X |
| yjfL | 3.04 |  |
| yfcH | 3.04 |  |
| ytfP | 3.04 | X |
| leuZ | 3.04 |  |
| allC | 3.03 |  |
| tatB | 3.03 |  |
| tatC | 3.03 | X |
| ssaU | 3.02 |  |
| ansB | 3.01 | X |

^a^Gene names according to ColiBase [3]

^b^When several oligonucleotides displaying significant enrichment corresponded to a single gene, the average enrichment over those oligonucleotides is shown.
