# Supplementary material for: Deep Sequencing Analysis of Small Noncoding RNA and mRNA Targets of the Global Post-Transcriptional Regulator, Hfq
Source: PLoS Genet. 2008 Aug 22;4(8):e1000163. doi: 10.1371/journal.pgen.1000163 (PMC2515195; doi:10.1371/journal.pgen.1000163)
Supplement: Table S6 — Oligodeoxynucleotides used in this study. (0.06 MB DOC) [file pgen.1000163.s011.doc]

**Table S6: Oligodeoxynucleotides used in this study**

| Name | Sequence |
| --- | --- |
| JVO-0686 | GGCCATGGAAAATGTAACCTTTGTAAG |
| JVO-0687 | GTTTTGAATTCATGGTTCGCCATTTTTATGA |
| JVO-1034 | GTTTTTTTTAATACGACTCACTATAGGATGAGGGGCATTTTATG |
| JVO-1035 | TTGCTGCAACGGTCAT |
| JVO-1117 | TCAGCCATTTTGTGCGCTT |
| JVO-1118 | TTCAGGATCGACAACGCCTT |
| JVO-1234 | aggtttggcattgtcgcct |
| JVO-1235 | CTTTTTCGAGCATCGGTGC |
| JVO-1236 | ACTATTGAGTCCCTCCCGGAAG |
| JVO-1237 | ACCGGACAATCCATGATAGCC |
| JVO-1342 | TCGCTTGCCGATTCACATT |
| JVO-1343 | CAATTTCTTCCGCACTCGGT |
| JVO-2152 | GTTTTCCATGGGAACAATGCATAC |
| JVO-2153 | GTTTTCTCGAGAACAGCCTGTTCGATC |
| JVO-2284 | GTTTTTAATACGACTCACTATAGGTTCCCGGCGACATCA |
| JVO-2285 | ATGTGTTTTAGCAACTCGGATG |
| JVO-2286 | GTTTTTAATACGACTCACTATAGGTCTATACGCCTGACTTTCCT |
| JVO-2287 | TTACAGTTACTGCAACCTTTG |
| JVO-2405 | CCTATGGGAGCGCGGTG |
| JVO-2406 | GTCAGAATACGACATTTTGGTACTC |
| JVO-2446 | GATAACCTGAGACCCCCCTG |
| JVO-2447 | AATACCCCCAAAAGCATTCG |
| JVO-2448 | ATATAAACGCGCCAGTCCAT |
| JVO-2471 | GTTTTTAATACGACTCACTATAGGATAAGCAAGGCTGGCAG |
| JVO-2472 | GCCTTGTAGAGAGTGGGG |
| JVO-3140 | CGGGTGGGATGAAATCGTAA |
| JVO-3141 | TTAGTGTCTGGCGAAACGCT |
| JVO-3142 | GTTGCTGCGGTGTAATAAGACA |
| JVO-3143 | TACGTTTGAGCTCAGGGTCG |
| JVO-3249 | AGAGAGTCAGCGCCGGG |
| JVO-3250 | AATTAAAACCACCCGCCG |
| JVO-3251 | CAGGCTACCAACCACCTCC |
| JVO-3252 | TATGGAGCGCAACGCC |
| JVO-3253 | GCGGTCTGGTGTACCTTCC |
| JVO-3254 | CGGGTCATCTTTCAGGCTG |
| JVO-3255 | TGCTTATACGCTACCGGGC |
| JVO-3256 | CTGCCTAACATCTCGTTTCTCC |
| JVO-3257 | GCCACGGTTCTCACCG |
| JVO-3258 | CAGCACACTACACAGGGTCG |
| JVO-3259 | ACCTTGCTGGCGCTCTC |
| JVO-3260 | CATCTTGCGGTCTGGCA |
| JVO-3261 | CATCGCGTTGCCAACTT |
| JVO-3262 | AAGACCCTGGCGCGGTT |
| JVO-3263 | CTTAGCAGCCTTGTAGAAGAGC |
| JVO-3264 | AAACTTGACACCGTTCGGC |
| JVO-3265 | GTGCCTCCGAACGGAAG |
| JVO-3266 | GCGACAATCACGCCCAG |
