# Supplementary material for: Deep Sequencing Analysis of Small Noncoding RNA and mRNA Targets of the Global Post-Transcriptional Regulator, Hfq
Source: PLoS Genet. 2008 Aug 22;4(8):e1000163. doi: 10.1371/journal.pgen.1000163 (PMC2515195; doi:10.1371/journal.pgen.1000163)
Supplement: Table S7 — Oligodeoxynucleotides used for Northern detection. (0.05 MB DOC) [file pgen.1000163.s012.doc]

**Table S7: Oligodeoxynucleotides used for Northern detection**

| Name | Sequence | Target region |
| --- | --- | --- |
| JVO-2405 | CCTATGGGAGCGCGGTG | STnc250 |
| JVO-2406 | GTCAGAATACGACATTTTGGTACTC | STnc290 |
| JVO-2407 | TTATTTGGACTACCTGGATG | STnc340 |
| JVO-2408 | TATGAGGAGGACAATTACCG | STnc440 |
| JVO-2445 | TACCGGACAATAATCCCTAC | STnc130 |
| JVO-2446 | GATAACCTGAGACCCCCCTG | STnc150 |
| JVO-2448 | ATATAAACGCGCCAGTCCAT | STnc180 |
| JVO-2466 | TCTGGCGGAACCTGCC | STnc220 |
| JVO-2468 | CACACCTGTCGGGCGTT | STnc310 |
| JVO-2469 | CGCAGTCCCAGGTCAGC | STnc330 |
| JVO-2498 | CTTATGTGGGCGTTTTGTTT | STnc350 |
| JVO-2499 | AATGACACCAACCTTTTACG | STnc390 |
| JVO-2500 | CTAGAGGAGGCGCTAGAAAG | STnc400 |
| JVO-3140 | CGGGTGGGATGAAATCGTAA | STnc190 |
| JVO-3141 | TTAGTGTCTGGCGAAACGCT | STnc400 |
| JVO-3142 | GTTGCTGCGGTGTAATAAGACA | STnc180 |
| JVO-3143 | TACGTTTGAGCTCAGGGTCG | STnc180 |
| JVO-3144 | TCATGTTACCGGTAAAATACCACC | STnc200 |
| JVO-3249 | AGAGAGTCAGCGCCGGG | STnc600 |
| JVO-3250 | AATTAAAACCACCCGCCG | STnc620 |
| JVO-3251 | CAGGCTACCAACCACCTCC | STnc590 |
| JVO-3252 | TATGGAGCGCAACGCC | STnc580 |
| JVO-3253 | GCGGTCTGGTGTACCTTCC | STnc610 |
| JVO-3254 | CGGGTCATCTTTCAGGCTG | STnc540 |
| JVO-3255 | TGCTTATACGCTACCGGGC | STnc560 |
| JVO-3256 | CTGCCTAACATCTCGTTTCTCC | STnc570 |
| JVO-3257 | GCCACGGTTCTCACCG | STnc480 |
| JVO-3258 | CAGCACACTACACAGGGTCG | STnc630 |
| JVO-3259 | ACCTTGCTGGCGCTCTC | STnc470 |
| JVO-3260 | CATCTTGCGGTCTGGCA | STnc490 |
| JVO-3261 | CATCGCGTTGCCAACTT | STnc500 |
| JVO-3262 | AAGACCCTGGCGCGGTT | STnc520 |
| JVO-3263 | CTTAGCAGCCTTGTAGAAGAGC | STnc640 |
| JVO-3264 | AAACTTGACACCGTTCGGC | STnc510 |
| JVO-3265 | GTGCCTCCGAACGGAAG | STnc530 |
| JVO-3266 | GCGACAATCACGCCCAG | STnc550 |
