# Supplementary material for: Deep Sequencing Analysis of Small Noncoding RNA and mRNA Targets of the Global Post-Transcriptional Regulator, Hfq
Source: PLoS Genet. 2008 Aug 22;4(8):e1000163. doi: 10.1371/journal.pgen.1000163 (PMC2515195; doi:10.1371/journal.pgen.1000163)
Supplement: Text S1 — Supplementary material and methods. (0.31 MB DOC) [file pgen.1000163.s013.doc]

**SUPPLEMENTARY MATERIAL**

**Deep sequencing analysis of small noncoding RNA and mRNA targets of the global post-transcriptional regulator, Hfq**

Alexandra Sittka, Sacha Lucchini, Kai Papenfort, Cynthia Sharma, Katarzyna Rolle,

Tim T. Binnewies, Jay C.D. Hinton and Jörg Vogel

**SUPPLEMENTARY MATERIALS AND METHODS**

*Analysis and visualization of pyrosequencing results*

After 5’end linker and polyA-tail clipping from the initial pyrosequencing results, all inserts ≥ 18 nt of the Hfq coIP and control coIP libraries were separately mapped to the *Salmonella* LT2 genome (NC_003197.fna) using WU-BLAST (http://blast.wustl.edu/). From the resulting blast positions one graph for each strand of the *Salmonella* chromosome was calculated, where the number of cDNA hits for each nucleotide position was plotted. To compare the graphs of the Hfq coIP and control coIP, the graphs were normalized to number of blastable reads. Following upload of the *Salmonella* genome sequence and annotation (NC_003197.fna and NC_003197.gff), the two graphs for each library were loaded into the Integrated Genome Browser (IGB) of Affymetrix (<http://www.affymetrix.com/support/developer/tools/download_igb.aff>, version [IGB-4.56](http://sourceforge.net/project/showfiles.php?group_id=129420&package_id=176795&release_id=487872)). Different panels show the annotations for the “+“ and “–“ strand (blue), the graphs for the “+” and “-“ strand of the Hfq coIP (red) and the control coIP (black), and the genome coordinates in the center. A specific genomic region can be selected for further analysis (*e.g.* SPI-1, Fig. 4).

SPI-1 represents an example of an entire genomic region highly enriched in the Hfq coIP library. In contrast, very few cDNA sequences mapping to SPI-1 are contained in the control coIP library. The flanking genes of *invR* (i.e. the border of SPI-1) nicely give an example of the specificity of the method (Fig. 4). While cDNAs mapping to the InvR sRNA gene represent the most abundant cluster in the Hfq coIP library, the genes in the closest proximity are barely represented in this library. In addition, the example of InvR underlines the reliability of the method to identify Hfq-dependent sRNAs.

The numbers of cDNA clones that overlap *Salmonella* sRNA genes or annotated ORFs are listed in Table S3 (sRNAs) and S4 (mRNAs). Comparing the numbers of cDNAs obtained for the Hfq coIP *vs.* control coIP, false positive sequences in the list of cDNAs of Hfq-bound RNAs were easily detected (see CsrB and CsrC, Table S3), based on the number of sequences received from the control coIP. In those cases the number of sequences was almost identical in the Hfq coIP and the control coIP libraries (note that the numbers are not normalized to the number of blastable reads.) While Table S3 and S4 only present absolute numbers of sequences, which map along an entire RNA transcript, it is necessary to analyze a gene of interest in detail. The ratio of hits per nucleotide of the Hfq coIP to hits from the control coIP provides a measure of enrichment, which can be visualized for every single nucleotide in a stepstair diagram. In the case of *ompD*, the number of cDNAs obtained in the Hfq coIP library (246) compared to those of control coIP library (76) lead to an overall enrichment factor of 3.3. Analysis of the single cDNA sequences over the entire transcript length reveals single “hot spots” in the transcript bound by Hfq, leading to enrichment factors up to 30 (for the region spanning the ATG start codon; Fig. 7A). However, the number of *ompA* cDNA sequences received in Hfq coIP (102) *vs.* control coIP (77) only lead to an overall enrichment factor of 1.3. At first glance, this would lead one to assume that *ompA* is not an Hfq-bound mRNA. However, closer inspection of the clone distribution along the entire mRNA, and of the enrichment of certain parts of the mRNA, reveals that the 5’ region (around ATG start codon) and a central region are highly enriched in the Hfq coIP (up to 12-fold; Fig. 7B)). Such detailed inspection offers the advantage of being able to analyze the 5’/3’ UTRs of mRNAs, which are not included in Table S4, but are known to be often bound by Hfq.

#### Quantitative RT-PCR

The wild-type strain SL1344 and JVS-0255 (Δ*hfq*) were grown in liquid culture from single colonies to an OD_600_ of 2 or for 12 hours under SPI-1 inducing conditions, respectively. Experiments were carried out as described previously [1]. Briefly, RNA was isolated using the SV40 Total RNA Isolation kit (Promega) according to manufacturer’s instructions. Expression of *rpoE*, *degP,* and *rpoS* mRNA was quantitatively assessed by qRT-PCR in a 7900HT (Applied Biosystems), with the *rfaH* gene as reference. For each reaction (25µL final vol.) 1µl of RNA sample (100 ng/ reaction) were mixed with 0.25µl of primer pairs (0.5 µM final) and 12.5 µL of SYBR Green mix (Qiagen). For coupled cDNA synthesis and target gene amplification 0.25µl of Quantitect RT mix was added. Each sample was assayed in triplicate for each run. Control RNA from wild-type cells was used to construct a standard curve for all inspected genes. Reaction conditions were: 30 min 50°C, 15 min 95°C, and 45 cycles at 94°C for 20 sec, 60°C for 40 sec, and 72°C for 40 sec. Oligodeoxynucleotides used in this experiment (JVO-1234/1235 (*degP*), JVO-1117/1118 (*rfaH*), JVO-1236/1237 (*rpoE*), JVO-1342/1343 (*rpoS*)) are listed in Table S6.

*Western blot*

Cultures were inoculated into fresh medium 1/100 from o/n cultures. Incubation was carried out under standard conditions to early stationary phase (OD_600_ of 2) or for 12 hours under SPI-1 inducing condition, respectively. Whole cell proteins were resolved by 12 % SDS PAGE and transferred to a PVDF membrane. Sigma proteins were detected using monoclonal antibodies against RpoE or RpoS, respectively (Neoclone).

*Northern analysis*

Cultures were inoculated into fresh medium 1/100 from o/n cultures. Incubation was carried out under standard conditions to logarithmic phase. RNA was extracted using TRIzol Reagent. For detailed protocol see [2].

|  |  |  |  |  |  |  |
| --- | --- | --- | --- | --- | --- | --- |
|  |  |  |  |  |  |  |
|  |  |  |  |  |  |  |
|  |  |  |  |  |  |  |
|  |  |  |  |  |  |  |
|  |  |  |  |  |  |  |
|  |  |  |  |  |  |  |
|  |  |  |  |  |  |  |
|  |  |  |  |  |  |  |
|  |  |  |  |  |  |  |
|  |  |  |  |  |  |  |
|  |  |  |  |  |  |  |
|  |  |  |  |  |  |  |
|  |  |  |  |  |  |  |
|  |  |  |  |  |  |  |
|  |  |  |  |  |  |  |
|  |  |  |  |  |  |  |
|  |  |  |  |  |  |  |
|  |  |  |  |  |  |  |
|  |  |  |  |  |  |  |
|  |  |  |  |  |  |  |
|  |  |  |  |  |  |  |
|  |  |  |  |  |  |  |
|  |  |  |  |  |  |  |
|  |  |  |  |  |  |  |
|  |  |  |  |  |  |  |
|  |  |  |  |  |  |  |
|  |  |  |  |  |  |  |
|  |  |  |  |  |  |  |
|  |  |  |  |  |  |  |
|  |  |  |  |  |  |  |
|  |  |  |  |  |  |  |
|  |  |  |  |  |  |  |
|  |  |  |  |  |  |  |
|  |  |  |  |  |  |  |
|  |  |  |  |  |  |  |
|  |  |  |  |  |  |  |
|  |  |  |  |  |  |  |
|  |  |  |  |  |  |  |
|  |  |  |  |  |  |  |
|  |  |  |  |  |  |  |
|  |  |  |  |  |  |  |
|  |  |  |  |  |  |  |
|  |  |  |  |  |  |  |
|  |  |  |  |  |  |  |
|  |  |  |  |  |  |  |
|  |  |  |  |  |  |  |
|  |  |  |  |  |  |  |
|  |  |  |  |  |  |  |
|  |  |  |  |  |  |  |
|  |  |  |  |  |  |  |
|  |  |  |  |  |  |  |
|  |  |  |  |  |  |  |
|  |  |  |  |  |  |  |
|  |  |  |  |  |  |  |
|  |  |  |  |  |  |  |
|  |  |  |  |  |  |  |
|  |  |  |  |  |  |  |
|  |  |  |  |  |  |  |
|  |  |  |  |  |  |  |
|  |  |  |  |  |  |  |

|  |  |  |  |  |  |
| --- | --- | --- | --- | --- | --- |
|  |  |  |  |  |  |
|  |  |  |  |  |  |
|  |  |  |  |  |  |
|  |  |  |  |  |  |
|  |  |  |  |  |  |
|  |  |  |  |  |  |
|  |  |  |  |  |  |
|  |  |  |  |  |  |
|  |  |  |  |  |  |
|  |  |  |  |  |  |
|  |  |  |  |  |  |
|  |  |  |  |  |  |
|  |  |  |  |  |  |
|  |  |  |  |  |  |
|  |  |  |  |  |  |
|  |  |  |  |  |  |
|  |  |  |  |  |  |
|  |  |  |  |  |  |
|  |  |  |  |  |  |
|  |  |  |  |  |  |
|  |  |  |  |  |  |
|  |  |  |  |  |  |
|  |  |  |  |  |  |
|  |  |  |  |  |  |
|  |  |  |  |  |  |
|  |  |  |  |  |  |
|  |  |  |  |  |  |
|  |  |  |  |  |  |
|  |  |  |  |  |  |
|  |  |  |  |  |  |
|  |  |  |  |  |  |
|  |  |  |  |  |  |
|  |  |  |  |  |  |
|  |  |  |  |  |  |
|  |  |  |  |  |  |
|  |  |  |  |  |  |
|  |  |  |  |  |  |
|  |  |  |  |  |  |
|  |  |  |  |  |  |
|  |  |  |  |  |  |
|  |  |  |  |  |  |
|  |  |  |  |  |  |
|  |  |  |  |  |  |
|  |  |  |  |  |  |
|  |  |  |  |  |  |
|  |  |  |  |  |  |
|  |  |  |  |  |  |
|  |  |  |  |  |  |
|  |  |  |  |  |  |
|  |  |  |  |  |  |
|  |  |  |  |  |  |
|  |  |  |  |  |  |
|  |  |  |  |  |  |
|  |  |  |  |  |  |
|  |  |  |  |  |  |
|  |  |  |  |  |  |
|  |  |  |  |  |  |
|  |  |  |  |  |  |
|  |  |  |  |  |  |
|  |  |  |  |  |  |
|  |  |  |  |  |  |
|  |  |  |  |  |  |
|  |  |  |  |  |  |
|  |  |  |  |  |  |
|  |  |  |  |  |  |
|  |  |  |  |  |  |
|  |  |  |  |  |  |
|  |  |  |  |  |  |
|  |  |  |  |  |  |
|  |  |  |  |  |  |
|  |  |  |  |  |  |
|  |  |  |  |  |  |
|  |  |  |  |  |  |
|  |  |  |  |  |  |
|  |  |  |  |  |  |
|  |  |  |  |  |  |
|  |  |  |  |  |  |
|  |  |  |  |  |  |
|  |  |  |  |  |  |
|  |  |  |  |  |  |

**References**

1. Papenfort K, Pfeiffer V, Mika F, Lucchini S, Hinton JC, et al. (2006) sigma(E)-dependent small RNAs of Salmonella respond to membrane stress by accelerating global omp mRNA decay. Mol Microbiol 62: 1674-1688.

2. Vogel J, Argaman L, Wagner EG, Altuvia S (2004) The small RNA IstR inhibits synthesis of an SOS-induced toxic peptide. Curr Biol 14: 2271-2276.

3. Chaudhuri RR, Khan AM, Pallen MJ (2004) coliBASE: an online database for Escherichia coli, Shigella and Salmonella comparative genomics. Nucleic Acids Res 32: D296-299.

4. Goto S, Bono H, Ogata H, Fujibuchi W, Nishioka T, et al. (1997) Organizing and computing metabolic pathway data in terms of binary relations. Pac Symp Biocomput: 175-186.

5. Sittka A, Pfeiffer V, Tedin K, Vogel J (2007) The RNA chaperone Hfq is essential for the virulence of Salmonella typhimurium. Mol Microbiol 63: 193-217.

6. Garcia-Vallve S, Guzman E, Montero MA, Romeu A (2003) HGT-DB: a database of putative horizontally transferred genes in prokaryotic complete genomes. Nucleic Acids Res 31: 187-189.

7. Papenfort K, Pfeiffer V, Lucchini S, Sonawane A, Hinton JC, et al. (2008) Systematic deletion of Salmonella small RNA genes identifies CyaR, a conserved CRP-dependent riboregulator of OmpX synthesis. Mol Microbiol in press.

8. Hershberg R, Altuvia S, Margalit H (2003) A survey of small RNA-encoding genes in Escherichia coli. Nucleic Acids Res 31: 1813-1820.

9. Padalon-Brauch G, Hershberg R, Elgrably-Weiss M, Baruch K, Rosenshine I, et al. (2008) Small RNAs encoded within genetic islands of Salmonella typhimurium show host-induced expression and role in virulence. Nucleic Acids Res.

10. Sharma CM, Darfeuille F, Plantinga TH, Vogel J (2007) A small RNA regulates multiple ABC transporter mRNAs by targeting C/A-rich elements inside and upstream of ribosome-binding sites. Genes Dev 21: 2804-2817.

11. Pfeiffer V, Sittka A, Tomer R, Tedin K, Brinkmann V, et al. (2007) A small non-coding RNA of the invasion gene island (SPI-1) represses outer membrane protein synthesis from the Salmonella core genome. Mol Microbiol 66: 1174-1191.

12. Figueroa-Bossi N, Lemire S, Maloriol D, Balbontin R, Casadesus J, et al. (2006) Loss of Hfq activates the sigma-dependent envelope stress response in Salmonella enterica. Mol Microbiol 62: 838-852.

13. Fortune DR, Suyemoto M, Altier C (2006) Identification of CsrC and characterization of its role in epithelial cell invasion in Salmonella enterica serovar Typhimurium. Infect Immun 74: 331-339.

14. Viegas SC, Pfeiffer V, Sittka A, Silva IJ, Vogel J, et al. (2007) Characterization of the role of ribonucleases in Salmonella small RNA decay. Nucleic Acids Res 35: 7651-7664.

15. Altier C, Suyemoto M, Ruiz AI, Burnham KD, Maurer R (2000) Characterization of two novel regulatory genes affecting Salmonella invasion gene expression. Mol Microbiol 35: 635-646.

16. Wassarman KM, Repoila F, Rosenow C, Storz G, Gottesman S (2001) Identification of novel small RNAs using comparative genomics and microarrays. Genes Dev 15: 1637-1651.
